# The efficacy of psychological prevention, and health promotion interventions targeting psychological health, wellbeing or resilience among forced migrant children and youth: a systematic review and meta-analysis

**DOI:** 10.1007/s00787-024-02424-8

**Published:** 2024-04-16

**Authors:** Clover Jack Giles, Maja Västhagen, Livia van Leuven, Anna Edenius, Ata Ghaderi, Pia Enebrink

**Affiliations:** 1 School of Behavioural, Social and Legal Sciences, Örebro University, Örebro, Sweden; 2 Department of Clinical Neuroscience, Karolinska Institutet, Stockholm, Sweden; 3 Department of Medicine, Karolinska Institutet, Stockholm, Sweden

**Keywords:** Forcibly displaced, Refugee, Promotion, Prevention, Psychological interventions, Children and youth

## Abstract

There are over 40 million displaced children and youth worldwide and there is a need promote their mental wellbeing. This study aimed to synthesize evidence regarding promotion interventions to increase wellbeing, resilience, and quality of life (primary outcomes), and prevention interventions to reduce internalizing and externalizing symptoms (secondary outcomes) in this population. The review protocol was registered with PROSPERO (CRD42022329978). Medline, PsycINFO, and Web of Science were searched. Inclusion criteria were: ≥ 10 participants, sample ≤ 18 years of age, no parental participation, explicated forced migrant populations, implementation in non-clinical context, and validated measures. Fifteen studies (*N* interventions = 18, *N* participants = 5741) were eligible. Two studies included outcomes related to wellbeing and quality of life. The remaining studies reported depression, PTSD, anxiety, internalizing and externalizing behaviours, and behavioural and emotional problems. There was only sufficient data to perform random-effects meta-analysis of depression scores. No significant effects were observed in comparison to control condition in randomized trials (*n* = 4994, *k* = 5) but a small significant positive trend was observed in within-group analyses (*n* = 537, *k* = 12). Cochrane’s risk of bias tools and the GRADE certainty of evidence tool were applied. No studies achieved low risk of bias and certainty of evidence was very low. In sum, there remains a dearth of rigorous intervention studies investigating the effects of promotive and preventative psychological interventions on the wellbeing, resilience, and quality of life of forcibly displaced minors. However, the few eligible studies in this review indicate promise.

**Supplementary Information:**

The online version contains supplementary material available at 10.1007/s00787-024-02424-8.

## Introduction

Under 18s account for 30% of the global population, but over 40% of forcibly displaced people. This amounts to over 41 million children and youth [1]. Forced displacement entails inherent pre-, peri-, and post-migration stressors [2] and necessitates adaptation to new environments [3]. Combined with the social determinants of health, these stressors can have additive effects, increasing the risk of mental health problems [4]. Many forcibly displaced children and youth have also experienced considerable traumas [5] and face ongoing hardships during transit [6] and resettlement [7, 8]. Around one in five refugee children and youth may be diagnosed with PTSD, 1 in 6 with anxiety disorders and 1 in 8 with depression [9]. However, there is also evidence suggesting that many forcibly displaced children and youth are adapting well in terms of their psychological wellbeing, acculturation, and education—especially among those resettling in high income countries [10–13] where there can be assumed to be greater political stability and considerably more resources. Similarly, it has repeatedly been found that most displaced children and youth do *not* suffer from clinically significant psychopathology [9, 14]. While it must be noted that most of this knowledge has been produced in high income countries with ample resources, it seems that populations of forcibly displaced children and youth may be deemed vulnerable, but not necessarily pathologically so.

Psychological wellbeing is a fundamental human right and is found among the United Nations Global Sustainable Development Goals [15, 16]. Wellbeing entails the presence of positive functions and subjective experiences that allow an individual to lead a fulfilling life [17], and includes both inter- and intrapersonal aspects, such as appraisals of own competence or development, and social belonging [18]. While psychological wellbeing and psychological distress are often placed on a continuum, they are not mutually exclusive [15, 19] meaning that wellbeing may be promoted even when distress is present [19]. Equally, as post migration stressors have been seen to pose a greater long-term risk for poor mental health than pre- and peri-migration stressors [20], it is important to ensure that children’s capacity to deal with hassles and potential traumas is maintained.

Psychological resilience is the capacity for positive development despite experiences of adversity [21] and is closely interrelated with wellbeing and quality of life [22]. Indeed, resilience has been identified in recent reviews of protective factors and individual outcomes among forcibly displaced children and youth [13, 23–26]. Factors such as safe play environments, social support, family contact, societal access, valuing education, and intercultural competence were identified. The importance of resilience for the psychosocial wellbeing and quality of life of forcibly displaced children and youth has been emphasised in both clinical and developmental psychology disciplines [3, 26]. However, internally displaced people, who comprise the largest forcibly displaced population, are under-represented in research samples [27], indicating that more work needs to be done.

Migrant populations may also experience numerous barriers to seeking psychiatric care [28], including stigma, mistrust of government service, and a lack of cultural and care system knowledge. Indeed, resettled forced migrant youth have been seen to access mental health care to a lower degree than their native-born counterparts [29]. Thus, interventions aiming to promote wellbeing and prevent mental health problems may be more accessible to migrant populations. Moreover, while there have been reports of iatrogenic effects of group prevention interventions for youth at risk of drug abuse [30] or delinquent behaviours [31]; the same has not been seen for interventions targeting mental health. Thus, promotive, and preventative interventions aiming to increase psychological wellbeing, that are implemented in community settings, may reduce stigma, require less care system knowledge, and potentially increase care equity for forcibly displaced children and youth [32](p. 20).

There is a growing body of literature on the resilience, wellbeing, and quality of life among forcibly displaced children and youth, but there have been fewer rigorous investigations of interventions aiming to promote and maintain them [5, 12, 27]. Promotional interventions aim to increase an individual’s control over, and ability to improve their own health [17]. Indeed, a recent meta-analysis of promotional positive psychology interventions [33] found significant small to medium effects on depression, anxiety, strengths (e.g., optimism, self-regulation, forgiveness, gratitude), and wellbeing in non-refugee populations, with larger effects observed in children and youth than other age groups. Moreover, results were partially sustained over time. Prevention interventions, on the other hand, aim to reduce the risk of clinical case development [32], and may be divided into universal (population wide), selective (for identified risk group) or indicated (for those with subclinical levels of symptoms or in nonclinical contexts) interventions. The boundaries between levels of intervention are however, to some extent arbitrary. For example, defining level of intervention with forced migrant populations is complicated by the fact that all members of the population have increased risk related to migration. Thus, some may argue that universal prevention is impossible with forced migrant populations in relation to the population at large. Indeed, level of prevention is seldom specified in research or review literature regarding interventions for displaced people [27]. In sum, mental health promotion, prevention, *and* treatments should be seen as complementary levels of intervention, and *all* can be necessary to ensure health and wellbeing at population level [32] (p. 592).

Much of the current review and meta-literature concerning forcibly displaced children and youth has aimed to synthesize evidence of the efficacy of psychological treatment*s* [34–37]. These studies conclude that psychosocial treatments such as cognitive behavioural therapy (CBT) and interpersonal therapy (IPT) may be effective at reducing psychological symptoms among forced migrant children and youth. However, they also conclude that there are very few methodologically robust intervention studies, and little to no knowledge about the long-term effects of these treatments. The two existing reviews of psychological interventions specifically for unaccompanied asylum seeking and refugee youth [38, 39] reached similar conclusions. Further, two meta-analyses [40, 41] and one review [42] have investigated the effects of prevention programs among broader populations, including both forced migrants and immigrants with unspecified migration history. All three concluded that psychological prevention programs may positively influence socio-emotional outcomes, especially when interventions are individualised or culturally tailored [40]. However, as numbers of forcibly displaced children and youth continue to grow, a better understanding of the efficacy of currently available preventative and promotive interventions is needed for the specific population.

### Objectives

A systematic description and meta-analysis of the current research concerning interventions aiming to increase wellbeing, resilience, and quality of life among forced migrant youth may inform much needed future research directions. This study therefore aimed to systematically identify and synthesize available knowledge regarding the efficacy of promotion and prevention interventions implemented in populations of forced migrant children and youth. In addition to wellbeing, resilience, and quality of life, this study investigated intervention effects on internalizing and externalizing behaviors and symptoms (e.g., depression, anxiety, PTSD, conduct problems) as these are common study outcomes. Further, this study aimed to describe how interventions were tailored for the population and which variables may predict outcomes, as this knowledge is currently lacking. This study had four objectives:

1. Evaluating immediate- and longer-term between-group changes in wellbeing, quality of life, and resilience (primary outcomes) and reductions in internalizing and externalizing behaviors and symptoms (secondary outcomes) at post-measurement and follow-ups (≥ 3-months).
2. Evaluating immediate- and longer-term within-group changes for participants in wellbeing, self-efficacy, resilience and reductions in internalizing and externalizing behaviors and symptoms at post-measurement and follow-ups (≥ 3-months).
3. Examining moderators and predictors (e.g., level of prevention, country of treatment, treatment components, cultural tailoring) related to reduced risk or symptomology and improved outcomes.
4. Describing theoretical underpinnings of interventions, intervention components and cultural tailoring.

## Methods

### Protocol and registration

This systematic review and meta-analysis was completed following the Prisma guidelines [43] (see Supplementary Information (SI) 1 and 2 for Prisma Checklists). The review protocol was published before the commencement of data-extraction on 2022-05-17 (PROSPERO 2022 CRD42022329978).

### Eligibility criteria

The study population was internally displaced, refugee and asylum-seekers ≤ 18 years of age, who participated in psychosocial interventions in non-clinical settings, without participation of parents or caregivers. Interventions including parents and/or caregivers were excluded as they were to be included in another ongoing meta-analysis (PROSPERO 2022 CRD42022330521). Moreover, parent/caregiver involvement was considered a potential confounder. We therefore chose to remove of studies including parents to increase applicability of review results for unaccompanied children and youth.

All modalities of guided mental health-promotion and prevention interventions programs (universal, selective, or indicated) delivered exclusively to children and youth in non-clinical settings, in any country, were eligible. Interventions offered to all forced migrants regardless of symptoms or experiences were categorised as universal. Interventions offered to participants with specific risk factors (e.g., poverty) were categorised as selected. Interventions that were offered to participants with symptoms of behavioural, emotional, internalizing or externalizing problems (including trauma, depression, anxiety) but without formal DSM or ICD diagnosis, and implemented in non-clinical community settings (including refugee camps/asylum accommodation) were categorized as indicated prevention. Interventions including only participants with clinical levels of symptoms/formal DSM/ICD-diagnosis or implemented in clinical settings (e.g., psychiatric open wards) were excluded.

Only quantitative or mixed methods intervention studies published in peer-reviewed journals, with ≥ 10 participants in the intervention group were eligible. Only randomized and cluster-randomized studies were eligible for study aim 1, while non-randomized designs (controlled and uncontrolled) were also eligible for study aim 2. All between-group conditions were allowed. Psychometric scales validated in any context, and explicitly measuring psychological wellbeing, quality of life, resilience, or internalizing and externalizing behaviours (including distinct psychological disorders) were necessary for inclusion. No limitations were placed on date or type of publication, and English, Danish, French, German, Norwegian, and Swedish language publications were considered for inclusion as these languages were spoken in the research team.

### Information sources and search strategy

Two search strategies were developed and implemented in collaboration with two librarians. Three databases were searched: MEDLINE, PsycINFO, and Web of Science Core Collection. Four major concepts were included in the initial strategy. These were: (1) refugee family populations including children and youth (2) mental health-related outcomes (3) psychosocial interventions (4) randomized controlled trials and uncontrolled study designs. The initial search was performed on the 10th of May 2021. The search was updated on the 11th of April 2022 with a broader search strategy to capture a greater variety of studies, and recently published studies. Both strategies are presented in full in SI 3. Both strategies were also used by Västhagen et al. (PROSPERO 2022 CRD42022330521) who conducted a review of interventions including parents and/or whole families.

Reference lists and the Web of Science citation index for all studies considered for inclusion, and of published reviews and meta-analysis within the subject areas were also reviewed by CJG and MV.

### Study selection

**Fig. 1:**
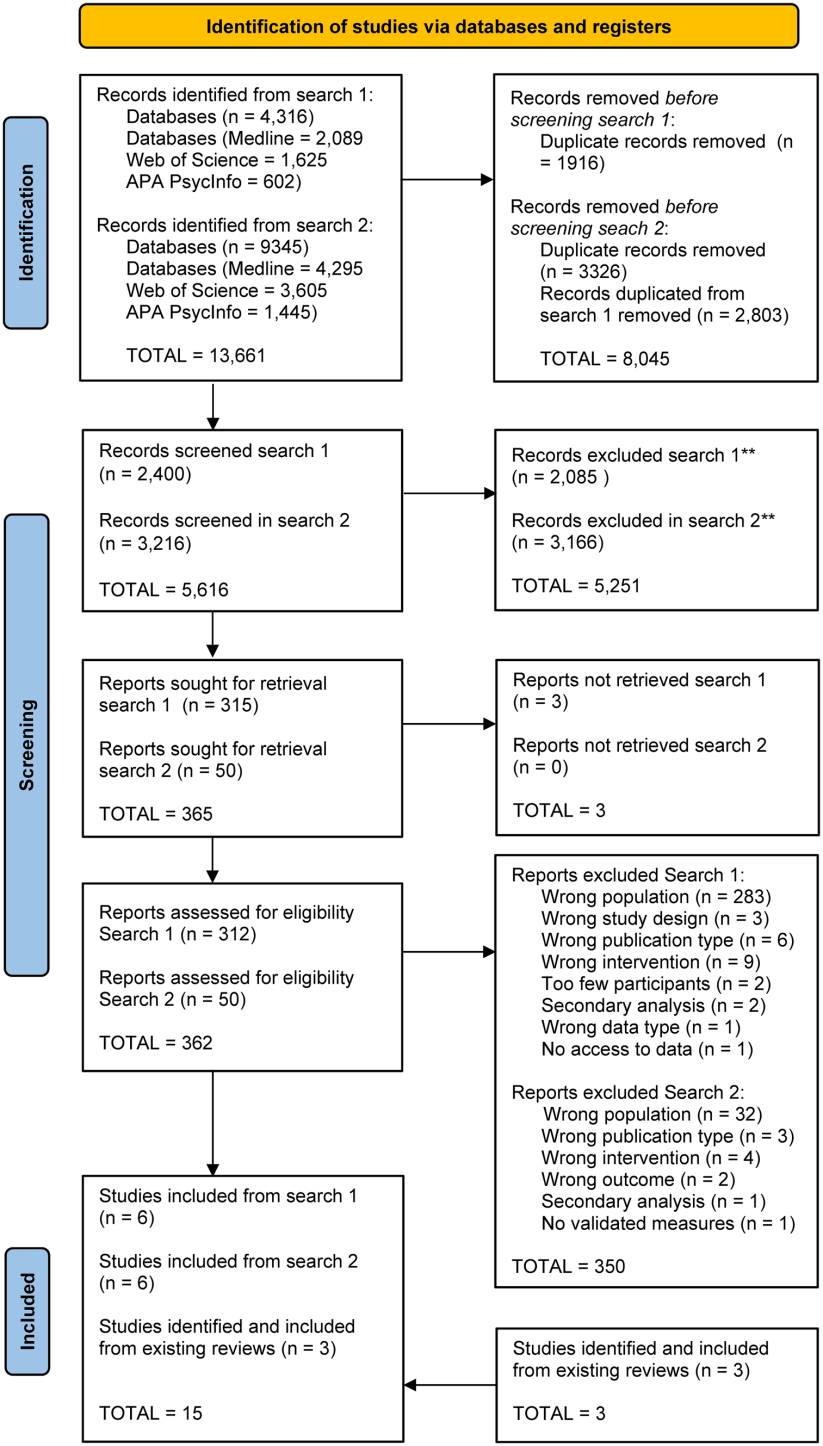
PRISMA 2020 flow diagram of inclusion process

The inclusion process is shown in the PRISMA flowchart (Fig. 1). Two pairs of independent reviewers (CJG & MV, AE & LVL) screened titles and abstracts found in search 1 using Rayyan [44]. Two independent reviewers (CGJ & MV) repeated the process after search 2. Potential studies identified by both reviewers were exported to EndNote X9 [45] and read independently in full-text. Inclusion decisions were checked in the aforementioned pairs and studies approved by both reviewers were included. A third part (PE) was consulted when mutual consensus could not be reached.

### Data collection process

All data was extracted to Excel [46] by CJG and MV. Nominal data was extracted collaboratively. Numerical data was extracted independently and checked collaboratively.

#### Data items

Data items included publication information, study characteristics, participant characteristics, intervention, county, intervention components, cultural adaptations, implementation setting, intervention length, intervention leader/s, recruitment procedure, outcome data/results including *N* at enrolment,* n* included in analysis, attrition, outcome variable, outcome measure, statistical analysis completed, means, standard deviations and effect sizes, length of follow-up, type of analysis. See SI 4 for a complete overview of data items.

### Risk of bias in individual studies

Risk of bias in the individual included studies was assessed using the Revised Cochrane Risk-of-Bias Tool for Randomized Trials [47], Cluster-Randomized Trials [48], and Non-randomized Studies of Interventions [49] assessment tools. The Cochrane tools assess several possible biases, including selection bias, performance bias, detection bias, attrition rate, reporting biases, etc. A pilot risk of bias assessment was conducted by three authors (CJG, MV & PE) to ensure interrater reliability. CJG and MV then collaboratively assessed of the remaining studies, while PE was consulted if consensus could not be reached. The overall result of the risk of bias assessment will be presented in the results as an aggregated variable.

### Summary of measures

The principal study measures were between-group differences in means at post intervention, or post intervention effect sizes reported in randomized controlled trials (RCT). Secondary study measures were differences in means between pre- and post- intervention measures reported in all study types. Pre- and post-intervention means and standard deviations, and/or effect sizes for all variables of interest were extracted.

### Synthesis of results

Results of individual studies for each included variable were summarized in tables, or in text when only one study reported the variable. Qualitative summaries were written for primary outcomes for which there were too few studies to perform meaningful statistical synthesis. All statistical analyses were completed using Comprehensive Meta-Analysis version 4 [50]. Pooled overall intervention-effects were estimated for eligible outcomes (outcomes present in ≥ 2 studies). Random effects meta-analyses were conducted using pooled post-intervention mean differences for outcomes present in ≥ 2 RCT studies (Study aim 1). Heterogeneity was assessed using *I*^*2*^- and *Q* statistics. Sensitivity analyses were conducted when outliers, or large residuals were identified, and when study weighting was very uneven. Within-group changes over time were compared for variables included in the between-group analyses, and tendencies were reported for other outcomes that occurred in ≥ 2 non-randomized studies (study aim 2).

### Additional analyses

Subgroup moderation analyses were performed in Comprehensive Meta-Analysis version 4 [50] when more than ten studies that reported the same outcome, in accordance with Cochrane recommendations [51].

### Risk of bias across studies

Publication bias was assessed in Comprehensive Meta-Analysis version 4 [50] using Egger’s test and funnel plots. Trim-and-fill methods were applied.

### Certainty of evidence

The GRADE system [52] was used to assess certainty of evidence for all statistical synthesis and reported in text as high (⨁⨁⨁⨁), moderate (⨁⨁⨁◯), low (⨁⨁◯◯), or very low (⨁◯◯◯). All assessments were completed collaboratively by CJG and MV. See SI 5 for the complete GRADE assessment.

## Results

### Study selection

The inclusion process is illustrated in Fig. 1 and an overview of the included studies, including results, is presented in Table 1. The two searches combined resulted in 13,661 hits. Thereafter, 8045 duplicates were removed, and 5616 reports proceeded to screening. After screening abstracts, 365 studies were read in full text, after which 25 studies were considered for inclusion. A further 4 studies were identified in the reference lists or the Web of Science Citation Index of the 25 considered studies and review literature. Fourteen [53–66] of these 29 studies were later excluded (see SI 6). Thus, 15 studies [67–81] were included. See SI 6 for a list of all studies excluded from full text review.

**Table 1:** Study characteristics, intervention, implementation location, sample, N, outcomes and results, overall risk of bias

Author and year	Study type	Intervention classification/s	Location	Participants, age (years), total % girls	N included (total = 5741)	Outcomes and results	Risk of bias
Bolton et al., 2007	RCT (3 arm)	IPT-G, Creative play therapy, Wait list control	Uganda	Internally displaced Ugandan Acholi youth (14–17, M = 15), 57%	314 (IPT-G = 105, creative play = 105, control = 104)	*Depression:* Difference in adjusted mean score change compared to control. IPT-G = 9.79, *SE* = 4.15, 95% CI [1.66–17.93]; creative play = − 2.51, *SE* = (NI) 95% CI [− 11.42 to 6.39]	Moderate
Cardeli et al., 2020	Pre-post	Trauma Systems Therapy for Refugees	USA	Bhutanese refugees (11–15, M = 12.94), 46%	35	*Depression: t*(30) = 0.108,* p* = 0.92, *d* = 0.02 *PTSD:* *t*(26) = 0.6.,* p* = 0.55, *d* = 0.11	Serious
Doumit et al., 2020	Pre-post	CBT	Lebanon	Syrian refugees (13–17, M = 14.22), 51.6%	40	*Quality of life:* *t*(28) = 2.09, *p* = 0.05, (*d* not reported) *Depression:* *t*(30) = 2.35,* p* = 0.025, *d* = 0.42 *Anxiety: t*(30) = 2.35, *p* = 0.025, *d* = 0.42	Serious
Ehntholt et al., 2005	RCT (quasi)	School based trauma focused CBT, Non-randomized wait list control	UK	Albanian (Kosovo, 11), Afghani (1), Kurdish (Türkiye, 3), Sierra Leonian (10), and Somali (1) refugees and asylum seekers, (11–15, M = 12.89), 35%	26 (CBT = 15, control = 11)	*Depression:* no significant differences between (ANCOVA) or within (t)groups were found *PTSD:* Between group ANCOVA, *F*(1,23) = 10.955, *p* = 0.003, within group intervention *t*(14) = 2.934, *p* = 0.011 control, *t*(10) = − 2.003, *p* = 0.073 *Anxiety:* Between group ANCOVA, *F*(1,23) = 6.495, *p* = 0.018, within group intervention *t*(14) = 1.581, *p* = 0.136, control *t*(10) = − 2.042,* p* = 0.068 *Teacher rated SDQ:* within group Wilcoxon signed rank test, intervention, *Z* = − 2.207, *p* = 0.027	Moderate
Foka et al., 2021	RCT (quasi)	Positive psychology intervention, Quasi randomized control	Greece	Afghani (13), Kurdistan (1), Lebanon (1), Stateless (2), Syrian (41) and unspecified nationality (2) refugees and displaced children and youth (7–14, M = 10.75), 63.90%	72 (intervention = 32, control = 36)	*Wellbeing: F*(1,46) = 42.99, *ηp*^*2*^ = .48, *p* < .001 *Depression:* Between group ANCOVA *F*(1,31) = 62.14, *ηp*^2^ = 0.67*, p* < 0.001, within group not reported	Moderate
Fox et al., 2005	Pre-post	School based CBT and skills training	USA	Vietnamese and Cambodian refugees, (6–16, M = 10) 65%	58	*Depression:* *t*(57) = 4.89, *p* = < 0.001	Serious
Garoff et al., 2018	Pre-post	Program inspired by trauma focused CBT	Finland	Afghani (7), Iraqi (2), and Unspecified nationality (9) refugees and asylum seekers, (9–17, M = 15.08) 11.11%	18	*PTSD:* *t*(9) = 1.30, *p* = 0.23, 95% CI [− 2.88, 10.68] *Caretaker rated SDQ:* *t*(11) = 0.23, *p* = 0.82, 95% CI [− 2.80, 3.46]	Serious
Gormez et al., 2017	Pre-post	CBT	Türkiye	Syrian refugees, (10–15, M = 12.41), 62.50%	32	*PTSD:* *t*(29) = 2.72, *p* = 0.011 *Anxiety:* *t*(31) = 3.73, *p* = 0.001 *Child rated SDQ:* *t*(30) = 2.44, *p* = 0.021	Moderate
Kalantari et al., 2012	RCT	Written trauma focused CBT	Iran	Afghani refugees, (12–18, M = 14.8), 55%	64 (intervention = 32, control = 32)	*PTSD:* between group ANCOVA *F*(60) = 12.97, *η*^2^ = 0.19, *p* = 0.001	Moderate
Ooi et al., 2016	RCT (cluster)	Trauma focused CBT, wait list control	Australia	Forcibly displaced people Africa, Asia, and the Middle East, (10–17, M = 12.59)	82 (intervention = 45, control = 37)	*Depression:* Between group Time × group ANOVA: *F*(1,155) = 5.20, *η*^2^ = 0.07, *p* = 0.024, *t*(155), 3.84, *p* = < 0.001, within group intervention *t*(155) = 3.84, *p* < 0.001, control *t*(155) = 0.47), *p* = 0.643 *PTSD:* Time × group ANOVA, *F*(1,154) = 3.09, partial *η*^2^ = 0.04, *p* = 0.081 *Parent rated SDQ:* Time × group ANOVA *F*(1,155) = 0.28, *ηp*^2^ = 0.00, *ns*	Moderate
Pfeiffer and Goldbeck, 2017	Pre-post	Trauma Focused CBT	Germany	Refugees from Afghani (14), Albani (2), Eritrean (3), Gambian (2), Ghanan (1), Iraqi (1), Nigerian (1), Pakistan (2), Somalia (1), Sudan (1) and Syria (1) (14–18, M = 16.7), 0%	36	*PTSD:* *t*(28) = 4.172, *p* = 0.001, 95% CI [3.68, 10.20], *d* = 0.97	Serious
Quinlan et al., 2016	Pre-post (inequivalent control)	Expressive arts therapy, inequivalent passive control	Australia	Refugees from Africa, East Asia and the Middle East, (13–17, M = 15.5), 59.5%	42 (arts = 22, control = 20)	*Depression:* between group, *t*(40) = − 0.32, *p* = 0.75, 95% CI [− 0.44 to 0.32], *d* = 0.1, within group mean difference, intervention -0.17 (0.71), control 0.23 (0.46) *Anxiety:* between group *t*(40) = − 0.53, *p* = 0.60, 95% CI [− 0.33,0.19], *d* = 0.17 *Teacher rated SDQ:* *t*(40) = 1.79, *p* = 0.08, 95% CI [− 0.31, 5.22], *d* = 0.57	Serious
Thabet et al., 2005	Cluster pre-post (3 arm, inc. control)	Psychological debriefing, Psychoeducation, Non-randomized wait list control	Gaza Strip	Palestinian refugees and internally displaced children and youth, (9–15, M = 12.10), 46.85%	111 (debriefing = 47, psychoeduc. = 22, control = 42)	*Depression:* Between group ANCOVA *F*(2, N = 111) = 1.45, *ns,* within group Wilcoxon test of change, Debriefing, *z* = 0.89, *ns,* psychoeducation, *z* = 0.34, *ns,* control, *z* = 01.2, *ns* *PTSD:* between group ANOVA *F*(2, N = 111) = 0.54, *ns. W*ithin group Wilcoxon test of change, Debriefing, *z* = 0.48, *ns,* Psychoeducation, *z* = 0.16, *ns,* control, *z* = 0.73, *ns*	Moderate
Tubbs Dolan et al., 2022	RCT cluster (3 arm)	Socio-emotional learning, Socio-emotional learning + Mindfulness, Treatment as usual	Lebanon	Syrian refugees, (5–15, M = 8.89), 49%	4784 (healing classroom = 1858, healing classroom + mindfulness = 1834, control = 1092)	*Depression:* Between group, unadjusted effect size healing classrooms ES = 0.0, *SE* = 0.01, *p* = 0.920; healing classrooms + mindfulness ES = 0.064, *SE* = 0.105, *p* = 0.540	Moderate
Ugurlu et al., 2016	Pre-post	Trauma therapy informed art therapy with developmental perspectives	Türkiye	Syrian refugees, (7–12, M = 9.16), 46%	63 (30 randomly chosen for post-test)	*Depression: t*(29) = 3.955, *p* = < 0.05, *g* = 0.72, 95% CI [0.20–1.24] *Parent rated PTSD:* *t*(24) = 5.45*, p* < 0.05,* g* = 1.00, 95% CI [0.45, 1.52] *Trait anxiety:* *t*(24) = 4.366, *p* < 0.05, *g* = 0.80, 95% CI [0.27, 1.32] *State anxiety:* *t*(25) = 1.010, p > 0.05	Serious

*RCT* randomized controlled trial, *IPT-G* Interpersonal Therapy-Group, *CBT* cognitive behavioural therapy, *ns* non-significant

### Study characteristics

Study characteristics are presented in full in SI 7. Of the 15 included studies, two were individually randomized RCTs [67, 75] ([67] = wait list control, [75] = passive control), two were cluster randomized RCTs [76, 80] ([76] = wait list control, [80] = treatment as usual), four were non-randomized controlled trials [70, 71, 78, 79] ([70, 71, 79]= wait list control, 78 = passive control), and the remainder were uncontrolled pre-post intervention studies. None included follow-up measures. The studies were published between 2005 and 2022. Three studies investigated the effects of two interventions [67, 79, 80]. Thus, the included studies assessed a total of 18 unique interventions.

### Risk of bias in individual studies

Overall risk of bias for each study is presented in Table 1. None of the included studies were assessed to have low risk of bias. Eight studies were assessed to have moderate risk of bias [67, 70, 71, 74–76, 79, 80] and seven were assessed to have high risk of bias [68, 69, 72, 73, 77, 78, 81]. All four RCTs were assessed to have moderate risk of bias. Some bias was expected as it is difficult to blind participants in studies of psychosocial interventions. Additionally, none of the studies had published a pre-specified data-analysis plan. Four of the non-randomized studies were assessed to have moderate risk of bias due to bias in measurement of results (i.e., inability to blind). The remainder were assessed to have high risk of bias as they had conducted no controls of baseline sample characteristics.

### Participant characteristics

A comprehensive overview of participants’ (*N* = 5741) characteristics is presented in SI 8. Of the included studies, 12 had < 100 participants, two has 100–350, and one had > 4500 participants. Participants were aged between 6 and 18 years, and 49.59% were girls. All studies reported gender as a binary variable and one study [77] included only boys. The participants were primarily classified as refugees (included in 13 studies). However, two studies each included internally displaced youth [67, 79], asylum seekers [70, 73], and forcibly displaced youth [71, 76]. Two studies included only unaccompanied youth [73, 77] and one study explicitly included both accompanied and unaccompanied youth [75]. Other studies made references to parents and/or caretakers. Most studies reported the participants’ ethnicity/country of origin, but two [76, 78] reported only geographical area (Africa, Middle East, and Asia). In total there were 24 distinct ethnicities/countries of origin reported, the studies were carried out in 11 different territories, including 8 in high income countries. The participants had been living in their current location from 2 months up to 7 years (4 studies did not report this variable [71, 72, 75, 79]).

### Primary outcomes: qualitative synthesis

Primary outcomes are presented in Table 2 and described qualitatively as only one study included validated measures of wellbeing [71], and quality of life [69], respectively. Although one study reported resilience [73] it did not use a validated measure.

**Table 2:** Summary of within and between group results for quality of life and wellbeing reported in individual studies

Author and year	Variable	n	Pre mean (SD)	Post mean (SD)	Statistical analysis	Results	Effect direction
Doumit et al., 2020	quality of life	29	82.20 (8.98)	88.77 (7.67)	*t*-tests (within group), *p*	*t*(28) = 2.09,* p* = 0.05	+
Foka et al., 2021 Intervention Wait list	wellbeing	33 39	41.67 (20.90) 40.77 (18.20)	88.27 (13.24) 47.89 (22.03)	ANCOVA (between group), *ηp2, p *	*F*(1,53) = 27.16,* ηp*^*2*^ = *0.34, p* < .001	+

Wellbeing was measured using the World Health Organisation Well-Being Index (WHO-5; [82]) by Foka et al. ([71], non-randomized controlled trial, moderate risk of bias). The intervention was a positive psychology intervention called *Strengths for the Journey* and consisted of six, daily 2-h group sessions for youth aged 6 to 17 (*M* age = 10; age matched groups were possible, 78.8% girls in intervention group, 52.6% girls in control group). The intervention was at the selected level and was developed and implemented in Greece, in transit camps for forcibly displaced people. The intervention manual is freely available at: https://www.qmul.ac.uk/sbbs/about-us/our-departments/psychology/strengths-for-the-journey-project/. The intervention was implemented in Arabic and Farsi languages by trained leaders and volunteer translators. No differences were found between the intervention and (non-randomized) control group in wellbeing scores at baseline (intervention *M* = 41.67, *SD* = 20.90; control *M* = 40.77, *SD* = 18.20; *t*(53) = − 0.17, *p* = 0.87) and scores were similar to previous established norms [82]. At post measurement, the intervention group reported significant and large increases in wellbeing compared to the control group (intervention *M* = 88.27, *SD* = 13.24; control *M* = 47.89, *SD* = 22.03; *F*(1,46) = 42.99, *ηp*^*2*^ = 0.48, *p* < 0.001) [71]. Sensitivity analyses were conducted to control for gender and baseline levels of self-esteem [71].

Quality of life was measured using the PedsQL Inventory-Version 4 for adolescents [83] by Doumit et al. ([69], pre-post study, assessed high risk of bias). The intervention was an existing Cognitive-Behavioural skill-building intervention called Creating Opportunities for Patient Empowerment program (COPE, [84]), and was intended to increase quality of life and promote positive mental health. The participants were 40 Syrian refugee youth (*M age* = 14.22, 48.4% girls) living with their families in Lebanon. The intervention was at the selected level of prevention and consisted of seven weekly hour-long sessions, implemented in a local community centre. No cultural tailoring was described, but the intervention was implemented by the study PI and Arabic speaking therapists who translated directly from the English language manual. The mean total score at pre-intervention was comparable with previously established general population means [83]. Significant improvements in total quality of life were observed from pre- to postintervention (pre *M* = 85.20, *SD* = 8.98; post *M* = 88.77, *SD* = 7.67; *t*(28) = 2.09, *p* = 0.05). However, the only subscale of the PedsQL [83] to exhibit significant improvements was the physical functioning scale.

### Secondary outcomes: description of studies

The most common secondary outcomes were child-rated symptoms of depression and PTSD. Child-rated depression was reported in 11 studies, which amounted to 14 unique interventions. Of these, 3 were RCTs [67, 76, 80] (5 interventions, all with moderate risk of bias) and 8 pre-post studies (3 with moderate risk of bias [70, 71, 79] and 5 with high risk of bias [68, 69, 72, 78, 81]). Child-rated PTSD was reported in 8 studies which amounted to 9 interventions (2 RCTs with moderate risk of bias [75, 76], 6 pre-post, 3 with moderate risk of bias [70, 74, 79] and 3 with high risk of bias [68, 73, 77]). However, the interventions and outcome measures reported in the two RCTs were not considered clinically similar. For example, one [75] was a written intervention implemented for traumatic grief symptoms over 3 days, while the other [76] was a group intervention for survivors of war or conflicts implemented over 8 weeks (see Table 1 for measures and SI 9 for a description of the interventions). PTSD was also parent-rated in one pre-post study [81] (serious risk of bias). Anxiety was reported in five non-randomized studies (5 interventions, 2 with moderate risk of bias [70, 74], 3 with high risk of bias [69, 78, 81]). A composite score of behavioural and emotional difficulties (measured using the child, teacher, or parent-rated Strengths and Difficulties Questionnaire; SDQ; [85], was reported in 5 studies (1 RCT with moderate bias [76], 4 pre-post, 2 with moderate risk of bias [70, 74], 2 with high risk of bias [73, 78]) which amounted to 5 interventions. Externalizing and Internalizing symptoms were reported in one study ([76], RCT, 1 intervention, moderate risk of bias). Statistics for secondary outcomes reported in individual studies are presented in SI 10.

### Results of syntheses

#### Between-group analysis (study aim 1)

**Fig. 2:**
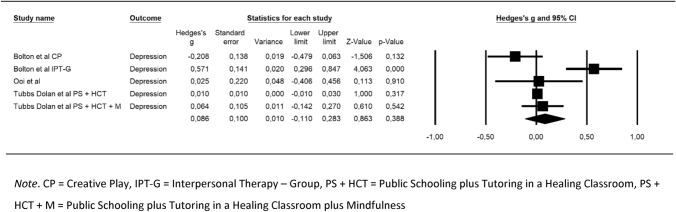
Forest plot for between-group effect sizes for depression

The only outcome possible to conduct a between-group meta-analysis of was depression (secondary outcome). Depression was measured in 3 RCT studies (5 interventions), all with moderate risk of bias. Two studies were at the indicated level of prevention [67, 76], while the remaining study [80] was at the universal/selected level. The results of the initial pooled random effects model (see Table 3) indicate a positive trend in favor of the intervention group but were not statically significant and showed serious levels of heterogeneity. The forest plot is displayed in Fig. 2. Analysis of the residuals identified one outlier (IPT-G [67]). A sensitivity analysis was performed but results remained even after removal of this intervention. Moreover, after removal this study [67] another intervention (Healing Classrooms + Mindfulness [80]) was seen to explain > 98% of the variance in the results, possibly due to the very large sample size. An additional sensitivity analysis, where both these interventions were excluded, indicated no problems with heterogeneity, residuals, or variance, but results remained non-significant and indicated a negative trend. There was not sufficient data to perform any sub-group analyses. No publication bias was detected and when using Trim and Fill values were unchanged. Certainty of evidence was very low (⨁◯◯◯).

**Table 3:** Between and within-group effect sizes (Hedge’s g) for depression

	*k*	*N*	*g *value	95% CI	*z* value	Q value	*I*^2^ (%)
Between-group effect	5	4994	0.09	− 0.11, 0.28	0.86	18.64	78.54
Within-group effect	12	537	0.51	0.26, 0.76	3.98*	73.60	85.05

*k* number of studies, *CI* confidence interval, *z* test of significance for *d*, *Q* statistical test of heterogeneity, *I*^2^ level of heterogeneity

*p < .001

#### Within group analyses (study aim 2)

*Depression* Within group comparisons of depression were possible for 10 studies (5 with moderate risk of bias, 5 with high risk of bias, 12 unique interventions, see SI 11). The pooled random effects model indicated significant improvements in depressive symptoms (*g* = 0.51, 95% CI [0.26, 0.76]). However, levels of heterogeneity were (see Table 3 and SI 11 for forest plot). A sensitivity analysis was conducted with one study removed due to large residuals ([71]; residual = 2.76). This analysis also indicated significant improvements in depressive symptoms, and levels of heterogeneity remained serious (*k* = 11, *g* = 0.44*,* 95% CI [0.20, 0.68],* z* = 3.59,* p* < 0.001, *Q* = 61.86, *I*^*2*^ = 83.84). No publication bias was detected and when using Trim and Fill values were unchanged. Certainty of evidence was very low (⨁◯◯◯).

#### Within group tendencies

Within group tendencies were investigated for PTSD, Anxiety, and Behavioral and Emotional Problems (measured using the SDQ [85]). See SI 7 for all study and outcome details and Table 1 for risk of bias assessments. Analyses indicated small positive trends for both child-rated symptoms of PTSD (*k* = 9) and anxiety, but levels of heterogeneity were serious. Behavioral and Emotional Problems (SDQ) were reported in 5 studies. The SDQ was child-rated in only one study [74] and therefore excluded from analysis. Analysis of the two teacher-rated SDQ scores [70, 78] indicated a small positive trend, however, analysis of the two 2 parent/caregiver rated SDQ scores [73, 76] indicated a small negative trend. Certainty of evidence was very low (⨁◯◯◯) for all investigated within-group tendencies except anxiety, where certainty of evidence was low (⨁⨁◯◯).

#### Subgroup analyses (study aim 3)

Five subgroup analyses of within-group changes in depression were performed based on the variables for which the most reliable data was available (see SI 12 for extended results and forest plots). The overall effect for age (*M* = < 12 vs. *M* ≥ 12) on depression was significant (*k* = 12, *g* = 0.53, 95% CI [0.32, 0.74], *z* = 4.91, *p* < 0.001, *Q*(1) = 10.59), indicating a medium effect size. A large effect size was observed for *M* age < 12 and small effect size for *M* age ≥ 12. The overall effect for gender (< 50% girls vs. > 50% girls) on depression was significant (*k* = 12, *g* = 0.45, 95% CI [0.22, 0.68], *z* = 3.86, p < 0.000, *Q*(1) = 0.18) indicating a small effect size. A medium effect size was observed for interventions with > 50% girls and small effect size for interventions with < 50% girls. The overall effect for context (intervention implemented in encampments vs. community settings) on depression was significant (*k* = 12, *g* = 0.50, 95% CI [0.26, 0.74],* z* = 4.10,* p* < 0.000, *Q*(1) = 0.13), indicating a medium effect size. A medium effect was observed for interventions implemented in camps and small effects for interventions implemented in community. The overall effect for participants being internally or externally displaced (including refugees and asylum seekers) on depression was significant (*k* = 12, *g* = 0.52, 95% CI [0.26, 0.78], *z* = 3.90, *p* < 0.000, *Q*(1) = 0.58), indicating a medium effect size. A small non-significant effect was observed for internally displaced participants and a medium effect for externally displaced participants. The overall effect of intervention level (universal/selected vs. indicated) on depression was significant (*k* = 12, *g* = 0.49, 95% CI [0.22, 0.75], *z* = 3.65,* p* < 0.000, *Q*(1) = 0.93), indicating a small effect size. A medium effect was observed for /universal/selected interventions and a small effect for indicated interventions.

### Intervention characteristics (study aim 4)

A summary of the intervention characteristics, including session content, is provided in SI 9. One of the 18 included interventions was operationalized as promotion [71], but also included elements of prevention. No interventions operationalized level of prevention, but 11 interventions (10 studies) were categorised at the indicated level, and 7 interventions at universal and/or selected level (including [71] that could be considered promotion). Two of the universal/selected interventions were part of a larger stepped care program. In subgroup analyses of depression, the promotive intervention [71] was included as universal/selective prevention. All interventions were implemented in group format (1–30 participants). Intervention length ranged from 1.5 [75] to 40 [80] hours, distributed over 3 days to 16 weeks (6–48 sessions).

There was considerable variation in how well theoretical underpinnings, intervention components, cultural tailoring and implementation language were described. The most common theoretical basis was cognitive behavioural therapy (8 interventions [69, 70, 72–77], of which 5 were trauma-focused [70, 73, 75–77]). Several other theoretical underpinnings were described, including socio-emotional learning theory [80], psychological debriefing [79], interpersonal therapy [67], and arts therapy programs with explicit psychosocial content [78, 81] (see SI 9 for remainder and details). Common intervention components were psychoeducation, skills training, exposure, and relaxation or mindfulness. Cultural tailoring was described in 11 studies (12 interventions) and ranged from providing gender segregated groups or live translation, to newly developed and fully tailored programs. One study [75] described no program tailoring but emphasised that the intervention allowed for cultural expression. Another study [81] emphasised that the non-verbal art-based modality allowed for individual (and it is assumed therewith also cultural) expression. The remaining 2 studies [70, 80] (3 interventions) described no cultural tailoring.

Most interventions were implemented in the participants’ mother tongue (either in their entirety or through translators). It was common for interventions to include bilingual facilitators, interpreters and/or cultural brokers, with interventions implemented in a combination of host country language and/or the participants’ mother tongue. Two of the included interventions were implemented exclusively in English [70, 76], and one in German [77]. Some studies did not explicate intervention language [67, 72, 73, 78, 79].

## Discussion

As numbers of displaced people continue to rise [1], the psychosocial wellbeing of forcibly displaced children and youth is an increasingly pressing issue. While it has been seen that many forcibly displaced children and youth do not suffer from clinical levels of mental health problems [9], their extraordinary life circumstances increase the risk of symptom debut. Moreover, migrants and forcibly displaced youth can experience multiple barriers to accessing psychiatric health care [28]. This indicates a considerable need for promotive and preventative mental health interventions implemented in community contexts. However, little is known about the efficacy of such interventions for the group. This systematic review and meta-analysis therefore aimed to synthesize knowledge regarding the effects of promotional and prevention interventions for forcibly displaced children and youth implemented in non-clinical settings; specifically, changes in wellbeing, quality of life, and resilience (primary outcomes) and reductions in a broad range of internalizing and externalizing behaviors (secondary outcomes). This study also aimed to examine moderators and predictors related to improved outcomes and to identify core intervention characteristics to guide best practice.

Fifteen studies were included. Of these, only one [71] included wellbeing as an outcome, and one [69] included quality of life. No other studies fulfilling the inclusion criteria included explicit, validated measures of our primary outcomes. One study [73] did include a composite measure of resilience, but this was not validated. Moreover, while almost half of the studies were assessed to have moderate risk of bias, none achieved low risk of bias, and all quantitative syntheses except within group improvements in anxiety were assessed to have very low certainty of evidence. Analyses of publication bias performed of the few possible analyses indicated that there were no missing studies. It can therefore be concluded that there is a dire lack of high quality studies regarding the effects of promotive and preventative psychosocial interventions for forcibly displaced children and youth, despite multiple calls for new intervention studies and meta-syntheses [5, 27, 34].

Nevertheless, both studies reporting wellbeing [71] and quality of life [69] reported post intervention improvements. Moreover, Foka et al. [71] included a (non-randomized) control group, and analyses indicated large intervention effects on wellbeing, even when gender and baseline self-esteem were controlled. The intervention was a newly developed positive psychology intervention, delivered in participants’ mother tongue, and specifically designed to increase psychological resources in forcibly displaced youth during transit. While strong conclusions cannot be drawn from single studies, these findings do suggest that promotive interventions aiming to increase the wellbeing and quality of life of forcibly displaced youth may have the potential to be efficacious.

All the included studies reported measures of mental health symptoms and/or dysfunctional behaviour. The most frequent outcome was depressive symptoms, followed by PTSD, anxiety, behavioural and emotional problems, and internalizing and externalizing behaviours. While the interventions were assessed to be preventative in nature, the lack of wellbeing related outcomes is disappointing, and endemic of overall tendencies in research where the absence of mental health problems is used as a proxy for wellbeing.

Meta-analysis of between group effects on depression reported in RCT studies indicated a small non-significant trend in favour of the intervention group. Within group analyses of intervention effects on depression indicated significant and positive medium effects. There were however serious heterogeneity problems in both analyses, which was unsurprising considering the inclusion criteria for this study. While heterogeneity naturally lowers the reliability of the cumulative evidence, these analyses do indicate that it may be possible to prevent or reverse the development of depressive symptoms among forcibly displaced children and youth.

Analyses of age and gender indicated that interventions with a higher proportion of young (< 12), or female participants were more successful at reducing depressive symptoms. This contradicts findings regarding the efficacy of group CBT for depression in other adolescent populations, where no effects of age or gender have been observed [86]. Our results may indicate that existing interventions are more acceptable for younger, and female participants. Alternatively, results of analyses of gender could possibly be explained by regression to the mean, as adolescent girls tend to report more depression that boys in refugee populations [6, 87]. The tendency observed for age is encouraging, as up to 50% of mental health problems debut before the age of 14, and 75% by 25 [32, 88]. Moreover, older forcibly displaced youth tend to report poorer overall mental health [7, 89] and fewer improvements over time [90]. The implementation of promotive and preventative interventions in early adolescence may thus have the potential to avert a potentially negative trajectory of symptom development, especially among girls.

Further subgroup analyses indicated that interventions were equally effective at reducing depression regardless of whether they were implemented in encampments, or permanent community settings. However, interventions including internally displaced participants (all of whom were in temporary encampments) had lesser effects than those including externally displaced participants. This may be understood to reflect the precarious living conditions of internally displaced people. However, caution must be taken when interpreting this result as the only positive psychology intervention [71] was implemented in a transit camp, and this study indicated the largest intervention effects on depression. Thus, the difference observed between camps vs. community, and internally vs. externally displaced may be better explained by intervention than participant or contextual characteristics. Nevertheless, examination of study weighting indicated that the positive psychology intervention [71] was not responsible for most of the effect in these analyses. However, there may be extraneous variables that our analyses cannot account for. As such, it may be cautiously concluded that promotive and preventative interventions for forcibly displaced youth are likely to be efficacious regardless of community or camp context but may be less efficacious in internal displacement camps.

Interestingly, subgroup analysis also indicated that selected and universal interventions may have greater effects on depression symptoms than indicated interventions. This contradicts previous findings regarding prevention of depression in broader populations [91]. It must be noted that the positive psychology intervention [71] which explicitly aimed to increase wellbeing (and had the largest positive effects on depression) was included in the universal/selected category. No subgroup analyses of theoretical underpinnings were conducted. However, visual analyses of forest plots of analysis of within group change in depression (SI 11) do not appear to indicate systematic variance based on theoretical background in this limited dataset. Nevertheless, the positive psychology intervention appears to show most promise.

Culturally relevant interventions, including both cultural awareness and cultural sensitivity, have been argued to improve acceptability and participant adherence [92], and both novel and culturally adapted interventions programs have been found to be more effective than unadapted intervention programs transported from other contexts [93]. The two studies reporting positive measures included in this analysis were examples of a novel and a transported program and mirrored these results. Strengths for the Journey [71] was developed in close contact with the target participants in the implementation setting. In contrast, COPE [69] was an unadapted intervention transported from a U.S. healthcare setting to Syrian refugee youth community settings in Lebanon. However, both intervention leaders in the COPE study spoke Arabic as their native language, meaning some degree of cultural adaptation was applied during the implementation. While conclusions cannot be drawn from single examples, it is interesting to note that Strengths for the Journey [71] was not only shown to increase wellbeing but also displayed the largest reductions in depressive symptoms of all the included studies.

A further aspect of cultural tailoring is implementation language. The included studies described several variations, including manuals developed in or translated to participants’ mother tongue, live translation of manuals by facilitators, implementation in host country language, implementation in mixed languages with bilingual facilitators, and implementation through interpreters. Additionally, some studies provided no details about implementation language. This heterogeneity (and imprecise description) meant we could not perform any predictive analysis of implementation language. However, it has previously been seen that professional interpreters can elevate the quality of clinical care for non-native speakers to similar levels as those receiving care in their native language [94]. This suggests that professional translators may be a valuable resource for reaching health care equity. Similarly, it has been emphasised that interpreters working with refugees in psychotherapy should have prior training specifically related to interpretation in therapy settings [95]. However, while professional translators would of course be desirable in all settings, it may be infeasible to include professional interpreters in some implementation settings for forcibly displaced populations. It must also be noted that these examples pertain to the use of interpreters in individual patient meetings between adults. Less is known about the role of translators in group interventions with youth participants. However, it has been seen that language (and associate cultural) barriers can be seen by both young refugees and care providers as a hinder to the therapeutic alliance, increasing distrust and potentially rendering talk based interventions “useless” [38]. Thus, implementation language deserves further attention in future studies for forcibly displaced children and youth. Future meta-analyses should aim to include studies published in a broader range of languages spoken in low- and middle-income countries to increase the likelihood that all available evidence is synthesized.

It was also noted that cultural tailoring was not reported in a systematic manner in any study. Therefore, categorical variables regarding tailoring could not be created to allow for sub-group analyses investigating the possible predictive effect of tailoring. Adherence to systematic reporting systems (such as the RECAPT guidelines [96]) in future studies could increase the validity of sub-group analyses in systematic reviews and meta-analyses aiming to guide best practice.

Notably, more than half of the included studies were conducted in high income countries despite the fact that high income countries host only a quarter of displaced populations [1]. This highlights the resource and care inequities faced by forcibly displaced people.

This study has several strengths and limitations. We have synthesized knowledge about a hard-to-reach and vulnerable population, which is important given the established difficulties in conducting conventional research with such groups [97]. We also implemented established guidelines regarding methodological quality, which not only increases the replicability of our study, but also the ability to compare results with meta-analyses of other populations or intervention using the same guidelines. Moreover, our stringent definition of the population possibly reduced causes of heterogeneity; the experiences of forcibly displaced and other migrant populations can differ considerably [90]. Thus, unclarities about whether the participants were forcibly displaced were a common reason for exclusion, especially among studies of Central and Latin American migrants in the U.S.A.. While this limited the number of studies applicable for this review and our results regarding the lack of high quality promotional and prevention intervention studies, it must be noted that this was somewhat expected. The lack of precise definition of forcibly displaced populations is understandable from both ethical, political, and practical standpoints, but it has been criticised as a weakness in existing research and meta-literature [26]. Moreover, none of the included studies defined level of prevention meaning we were necessitated to assign this variable for analysis based on our assessment of intervention descriptions and inclusion criteria. The precise definition of boundaries between universal and selective prevention for this particular population is a matter of debate. For example, whether interventions offered to refugees could be considered universal or must be designated as selected as refugee experience may be seen as a risk factor. Our own categorisations may therefore be considered arbitrary. This study once again serves to illustrate the complexity of developing and synthesizing specific empirical knowledge about promotion and prevention intervention efficacy for such populations. However, as Schenker et al. [97] so eloquently put it “the perfect (study) should not be the enemy of the good, particularly when the need […] is so critical (p. 20).”

This study only included explicit and validated measures of the target outcomes. While this may be seen as a sign of methodological rigour, we also acknowledge that there are weaknesses associated with this criterion. While all measures were validated, few were validated for refugees of the specific cultural group. Moreover, in choosing to include only explicitly measures of wellbeing, resilience, and quality of life, we excluded three studies that reported on social skills [53], adaptive skills [55], and social competence [56], and did not analyse the measure of school belongingness present in one of the included studies [68]. All four of these variables could be considered components of resilience. Thus, future reviews and meta-analyses should strive to include measures validated for refugees of the specific cultural group, and to operationalise resilience as an umbrella term to capture all relevant components reported in the existing literature.

Furthermore, assessing the methodological quality and risk of bias of psychological intervention studies is not without problems [34, 36]. While it is important to adhere to recommended methods of assessment, the Cochrane risk of bias instruments may be less suitable for clinical psychological research. All studies included in this analysis had an increased risk of bias due to the lack of blind measurements. However, very few psychological intervention studies can achieve true measurement blindness. It is therefore difficult to obtain low risk of bias in psychological interventions studies using the Cochrane tools. Thus, the lack of low risk assessments could be seen as a result of investigating the effects of psychosocial interventions rather than of research challenges related to the study of forcibly displaced populations.

## Conclusions

This systematic review and meta-analysis once again illuminates the lack of promotion and prevention studies measuring positive psychological outcomes among forcibly displaced children and youth that adhere to the gold standards of intervention research. The few analyses that were possible to conduct indicate that psychological promotion and prevention interventions may be efficacious at reducing symptoms of depression in forcibly displaced children and youth. Similar tendencies were observed for PTSD, anxiety, and emotional and behavioural problems. Caution must be taken when interpreting the cumulative evidence presented due to the small number of studies included, low certainty of evidence, lack of follow-up data, and considerable heterogeneity—both regarding challenges within this field of research and in the studies included in this analysis. However, several interesting and creative intervention initiatives were revealed during the review process, suggesting that there is hope for increased quality in future synthesis of knowledge. Nevertheless, to allow greater understanding of positive intervention outcomes among forcibly displaced children and youth there is a need for a paradigm shift in the measurement of intervention efficacy, where improved wellbeing, resilience, and quality of life are explicitly measured, and not merely assumed to be the biproduct of reduced symptomology.

## Supplementary Information

Below is the link to the electronic supplementary material.

Supplementary file1 (DOCX 31 KB)

Supplementary file2 (DOCX 18 KB)

Supplementary file3 (DOCX 47 KB)

Supplementary file4 (DOCX 24 KB)

Supplementary file5 (DOCX 56 KB)

Supplementary file6 (DOCX 78 KB)

Supplementary file7 (DOCX 28 KB)

Supplementary file8 (DOCX 26 KB)

Supplementary file9 (DOCX 29 KB)

Supplementary file10 (DOCX 51 KB)

Supplementary file11 (DOCX 143 KB)

Supplementary file12 (DOCX 745 KB)

## Data Availability

Please contact the first author.
