# Supplementary material for: The efficacy of psychological prevention, and health promotion interventions targeting psychological health, wellbeing or resilience among forced migrant children and youth: a systematic review and meta-analysis
Source: Eur Child Adolesc Psychiatry. 2024 Apr 16;34(1):123–40. doi: 10.1007/s00787-024-02424-8 (PMC11805832; doi:10.1007/s00787-024-02424-8)
Supplement: Supplementary file 3 — Supplementary file3 (DOCX 47 KB) [file 787_2024_2424_MOESM3_ESM.docx]

Supplementary Information 3

**The efficacy of psychological prevention, and health promotion interventions targeting psychological health, wellbeing or resilience among forced migrant children and youth: a systematic review and meta-analysis**

**European Child and Adolescent Psychiatry**

Clover Jack Giles ^1^, Maja Västhagen ^2^, Livia Van Leuven ^2^,

Anna Edenius^3^, Ata Ghaderi ^2^, Pia Enebrink ^2^

^1^ School of Behavioural, Social and Legal Sciences, Örebro University, Örebro, Sweden

^2^ Department of Clinical Neuroscience, Karolinska Institutet, Stockholm, Sweden

^3^ Department of Medicine, Karolinska Institutet, Stockholm, Sweden

*Corresponding author:*

Clover Jack Giles (CJG)

[clover.giles@oru.se](mailto:clover.giles@oru.se)

# Supplementary Information 3: Documentation of Search Strategy

Date: Search 1: May 2021 + Search 2: April 2022

Topic/research question: **The efficacy of promotion and prevention interventions targeting psychological well-being and symptoms of Depression, Anxiety, Stress and Trauma among involuntary migrants: a systematic review and meta-analysis**

Name of researchers: REMOVED FOR BLIND REVIEW

Databases:

1. Medline (OVID)
2. PsycInfo (OVID)
3. Web of Science (Clarivate)

Total number of hits:

- Before deduplication: search 1: 4,316 search 2: 9,345 **total: 13,661**
- After within search deduplication: search 1: 2,400 search 2: 6,029 **total: 8,429**
- After between search deduplication: search 2: 3226 **total 5,616**

### Search 1.

Medline

| Interface: Ovid MEDLINE(R) and Epub Ahead of Print, In-Process & Other Non-Indexed Citations and Daily  Date of Search: 7^th^ May 2021  Number of hits: 2,089  Comment: In Ovid, two or more words are automatically searched as phrases; i.e. no quotation marks are needed | Field labels   - exp/ = exploded MeSH term - / = non exploded MeSH term - .ti,ab,kf. = title, abstract and author keywords - adjx = within x words, regardless of order - * = truncation of word for alternate endings |
| --- | --- |
| 1. exp "Emigrants and Immigrants"/  2. exp Human Migration/ 3. Refugees/  4. "Transients and Migrants"/  5. (asylum seek* or diaspora or emigrat* or emigrant* or floating population or immigrat* or immigrant* or migrat* or migrant* or newcomer* or newly arrived or refugee*).ti,ab,kf.  6. (displac* adj2 (adolescen* or boy? or child* or famil* or father* or forced or girl? or internal* or mass or minor* or mother* or parent* or person* or people* or population* or youth*)).ti,ab,kf.  7. or/1-6  8. Adjustment Disorders/  9. Antisocial Personality Disorder/  10. Anxiety/  11. exp Anxiety Disorders/  12. Child Behavior Disorders/  13. Depression/  14. Depressive Disorder/  15. Emotional Adjustment/  16. Mental Health/  17. Psychological Trauma/  18. Quality of Life/  19. exp Stress Disorders, Traumatic/  20. Stress, Psychological/  21. (anxiety or agoraphobia or antisocial behavio* or anxious* or depression or PTSD or quality of life or social phobia or war neuros* or well-being).ti,ab,kf.  22. ((disorder* or symptom*) adj2 (adjustment or affective or antisocial or anxiety or child behavio* or depressive or dysthymic or mood* or panic or phobi* or stress)).ti,ab,kf.  23. (grief or grieving).ti,ab,kf.  24. (mental* adj (disease* or disorder* or health or ill* or well-being)).ti,ab,kf.  25. (psycho* adj (distress* or stress or trauma)).ti,ab,kf.  26. or/8-25  27. 7 and 26  28. Cognitive Behavioral Therapy/  29. exp Desensitization, Psychologic/  30. Health Promotion/  31. Psychotherapy, Brief/  32. Psychotherapy, Psychodynamic/  33. (CBT or TF-CBT or EMDR or ISTDP or PDT).ti,ab,kf.  34. (cognitive adj2 therapy).ti,ab,kf.  35. (desensitization adj2 (eye movement* or psychologic*)).ti,ab,kf.  36. ((exposure or flooding or implosive or narrative or prolonged exposure) adj therapy).ti,ab,kf.  37. (psychotherapy adj (brief or dynamic or psychodynamic or short-term)).ti,ab,kf.  38. (intervention* or prevent* or program* or rehabilitation or therap* or treat*).ti,ab,kf.  39. (prevention & control or psychology or rehabilitation or therapy).fs.  40. or/28-39  41. Randomized Controlled Trial.pt.  42. Controlled Clinical Trial.pt.  43. Clinical Trials as Topic.sh.  44. randomi?ed.ab.  45. randomly.ab.  46. rct.ti,ab.  47. trial.ti.  48. Observational Study.pt.  49. (observ* adj3 (study or studies)).ab,ti.  50. exp Cohort Studies/  51. (cohort adj (study or studies)).ab,ti.  52. (cohort analy* or longitudinal or prospective* or retrospective*).ab,ti.  53. (follow up adj (study or studies)).ab,ti.  54. or/41-53  55. 7 and 26 and 40 and 54  56. limit 55 to (danish or english or french or german or norwegian or swedish) | |

Web of Science Core Collection

| Interface: Clarivate Analytics  Date of Search: 7^th^ 2021  Number of hits: 1,625 | Field labels   - TS/Topic = title, abstract, author keywords and Keywords Plus - NEAR/x = within x words, regardless of order - * = truncation of word for alternate endings   Note: sometimes “quotation marks” are needed for single search terms to avoid automatic term mapping (lemmatization). |
| --- | --- |
| #1 (”asylum seek*” or diaspora or emigrat* or emigrant* or ”floating population” or immigrat* or immigrant* or migrat* or migrant* or newcomer* or ”newly arrived” or refugee*) OR (displac* NEAR/1 (adolescen* or boy? or child* or famil* or father* or forced or girl? or internal* or mass or minor* or mother* or parent* or person* or people* or population* or youth*))  #2 (anxiety or agoraphobia or ”antisocial behavio*” or anxious* or depression or PTSD or ”quality of life” or ”social phobia” or ”war neuros*” or ”well-being”) OR ((disorder* or symptom*) NEAR/1 (adjustment or affective or antisocial or anxiety or ”child behavio*” or depressive or dysthymic or mood* or panic or phobi* or stress)) OR (grief or grieving) OR (mental* NEAR (disease* or disorder* or health or ill* or ”well-being”)) OR (psycho* NEAR (distress* or stress or trauma))  #3 (cognitive NEAR/1 therapy) OR (desensitization NEAR/1 (”eye movement*” or psychologic*)) OR  ((exposure or flooding or implosive or narrative or ”prolonged exposure”) NEAR therapy) OR  (psychotherapy NEAR (brief or dynamic or psychodynamic or ”short-term”)) OR (intervention* or prevent* or program* or rehabilitation or therap* or treat*) OR (CBT or ”TF-CBT” or EMDR or ISTDP or PDT)  #4 (”clinical trial” or randomi?ed or randomly or RCT) OR (observ* NEAR/2 (study or studies)) OR  (cohort NEAR (study or studies)) OR (”cohort analy*” or longitudinal or prospective* or retrospective*) OR (”follow up” NEAR (study or studies))  #5 #1 AND #2 AND #3 AND #4 **Refined by:** **LANGUAGES:** ( ENGLISH OR FRENCH OR DANISH OR GERMAN ) AND **DOCUMENT TYPES:** ( ARTICLE OR EARLY ACCESS OR REVIEW OR RETRACTED PUBLICATION ) | |

Psychinfo

| Interface: Ovid  Date of Search: 7^th^ May  Number of hits: 602  Comment: In Ovid, two or more words are automatically searched as phrases; i.e. no quotation marks are needed | Field labels   - exp/ = exploded controlled term - / = non exploded controlled term - .ti,ab,id. = title, abstract and author keywords - adjx = within x words, regardless of order - * = truncation of word for alternate endings |
| --- | --- |
| 1. immigration/  2. exp human migration/  3. (asylum seek* or diaspora or emigrat* or emigrant* or floating population or immigrat* or immigrant* or migrat* or migrant* or newcomer* or newly arrived or refugee*).ti,ab,id.  4. (displac* adj2 (adolescen* or boy? or child* or famil* or father* or forced or girl? or internal* or mass or minor* or mother* or parent* or person* or people* or population* or youth*)).ti,ab,id.  5. or/1-4  6. antisocial personality disorder/  7. anxiety/  8. exp anxiety disorders/  9. child behavior/  10. "depression (emotion)"/  11. exp major depression/  12. exp emotional adjustment/  13. exp mental health/  14. emotional trauma/  15. "quality of life"/  16. exp "stress and trauma related disorders"/  17. psychological stress/  18. (anxiety or agoraphobia or antisocial behavio* or anxious* or depression or PTSD or quality of life or social phobia or war neuros* or well-being).ti,ab,id.  19. ((disorder* or symptom*) adj2 (adjustment or affective or antisocial or anxiety or child behavio* or depressive or dysthymic or mood* or panic or phobi* or stress)).ti,ab,id.  20. (grief or grieving).ti,ab,id.  21. (mental* adj (disease* or disorder* or health or ill* or well-being)).ti,ab,id.  22. (psycho* adj (distress* or stress or trauma)).ti,ab,id.  23. or/6-22  24. exp cognitive behavior therapy/  25. eye movement desensitization therapy/  26. health promotion/  27. brief psychotherapy/  28. psychodynamic psychotherapy/  29. (CBT or TF-CBT or EMDR or ISTDP or PDT).ti,ab,id.  30. (cognitive adj2 therapy).ti,ab,id.  31. (desensitization adj2 (eye movement* or psychologic*)).ti,ab,id.  32. ((exposure or flooding or implosive or narrative or prolonged exposure) adj therapy).ti,ab,id.  33. (psychotherapy adj (brief or dynamic or psychodynamic or short-term)).ti,ab,id.  34. (intervention* or prevent* or program* or rehabilitation or therap* or treat*).ti,ab,id.  35. or/24-34  36. 5 and 23 and 35  37. (clinical trial or randomi?ed or randomly or RCT).ti,ab.  38. (observ* adj3 (study or studies)).ab,ti.  39. (cohort adj (study or studies)).ab,ti.  40. (cohort analy* or longitudinal or prospective* or retrospective*).ab,ti.  41. (follow up adj (study or studies)).ab,ti.  42. or/37-41  43. 36 and 42  44. limit 43 to (danish or english or french or german or norwegian or swedish) | |

### Search 2:

Medline

| Interface: Ovid MEDLINE(R) and Epub Ahead of Print, In-Process & Other Non-Indexed Citations and Daily  Date of Search:11 April 2022  Number of hits: 4,295  Comment: In Ovid, two or more words are automatically searched as phrases; i.e. no quotation marks are needed | Field labels   - exp/ = exploded MeSH term - / = non exploded MeSH term - .ti,ab,kf. = title, abstract and author keywords - adjx = within x words, regardless of order - * = truncation of word for alternate endings |
| --- | --- |
| Database(s): **Ovid MEDLINE(R) and Epub Ahead of Print, In-Process, In-Data-Review & Other Non-Indexed Citations and Daily**1946 to April 08, 2022 Search Strategy:   \| **#** \| **Searches** \| **Results** \| \| --- \| --- \| --- \| \| 1 \| exp "Emigrants and Immigrants"/ \| 14627 \| \| 2 \| exp Human Migration/ \| 27391 \| \| 3 \| Refugees/ \| 12032 \| \| 4 \| "Transients and Migrants"/ \| 13287 \| \| 5 \| (asylum seek* or diaspora or emigrat* or emigrant* or floating population or immigrat* or immigrant* or migrat* or migrant* or newcomer* or newly arrived or refugee*).ti,ab,kf. \| 425918 \| \| 6 \| (displac* adj4 (adolescen* or boy? or child* or famil* or father* or forced or girl? or internal* or mass or minor* or mother* or parent* or person* or people* or population* or youth*)).ti,ab,kf. \| 6109 \| \| 7 \| or/1-6 \| 445502 \| \| 8 \| Adjustment Disorders/ \| 4285 \| \| 9 \| Antisocial Personality Disorder/ \| 10087 \| \| 10 \| Anxiety/ \| 97008 \| \| 11 \| exp Anxiety Disorders/ \| 86109 \| \| 12 \| Child Behavior Disorders/ \| 20723 \| \| 13 \| Depression/ \| 139418 \| \| 14 \| Depressive Disorder/ \| 74639 \| \| 15 \| Emotional Adjustment/ \| 949 \| \| 16 \| Mental Health/ \| 51894 \| \| 17 \| Psychological Trauma/ \| 1781 \| \| 18 \| Quality of Life/ \| 237751 \| \| 19 \| Resilience, Psychological/ \| 7765 \| \| 20 \| exp Stress Disorders, Traumatic/ \| 42315 \| \| 21 \| Stress, Psychological/ \| 130519 \| \| 22 \| (anxiety or agoraphobia or antisocial behavio* or anxious* or depression or PTSD or quality of life or social phobia or war neuros* or well-being).ti,ab,kf. \| 905500 \| \| 23 \| ((disorder* or symptom*) adj2 (adjustment or affective or antisocial or anxiety or child behavio* or depressive or dysthymic or mood* or panic or phobi* or stress)).ti,ab,kf. \| 223991 \| \| 24 \| (grief or grieving).ti,ab,kf. \| 9132 \| \| 25 \| (mental* adj (disease* or disorder* or health or ill* or well-being)).ti,ab,kf. \| 263928 \| \| 26 \| (psycho* adj (distress* or stress or trauma)).ti,ab,kf. \| 43064 \| \| 27 \| Parenting/ \| 19545 \| \| 28 \| exp Parent-Child Relations/ \| 59622 \| \| 29 \| Self Efficacy/ \| 23030 \| \| 30 \| ((adolescen* or boy? or child* or famil* or father* or girl? or minor* or mother* or parent* or youth*) adj3 (conflict* or interact* or relation*)).ti,ab,kf. \| 80921 \| \| 31 \| (competence or confidence or parenting or resilienc* or self-efficacy or skill*).ti,ab,kf. \| 940492 \| \| 32 \| or/8-31 \| 2294159 \| \| 33 \| Cognitive Behavioral Therapy/ \| 28675 \| \| 34 \| exp Desensitization, Psychologic/ \| 4154 \| \| 35 \| Health Promotion/ \| 79081 \| \| 36 \| Psychotherapy, Brief/ \| 3647 \| \| 37 \| Psychotherapy, Psychodynamic/ \| 708 \| \| 38 \| (CBT or TF-CBT or EMDR or ISTDP or PDT).ti,ab,kf. \| 30040 \| \| 39 \| (cognitive adj2 therap*).ti,ab,kf. \| 25912 \| \| 40 \| (desensitization adj2 (eye movement* or psychologic*)).ti,ab,kf. \| 635 \| \| 41 \| ((exposure or flooding or implosive or narrative or prolonged exposure) adj therapy).ti,ab,kf. \| 2336 \| \| 42 \| (psychotherapy adj (brief or dynamic or psychodynamic or short-term)).ti,ab,kf. \| 53 \| \| 43 \| (intervention* or prevent* or program* or rehabilitation or therap* or treat*).ti,ab,kf. \| 9822781 \| \| 44 \| (prevention & control or psychology or rehabilitation or therapy).fs. \| 4371370 \| \| 45 \| or/33-44 \| 11780422 \| \| 46 \| Randomized Controlled Trial.pt. \| 564125 \| \| 47 \| Controlled Clinical Trial.pt. \| 94806 \| \| 48 \| Clinical Trials as Topic.sh. \| 199674 \| \| 49 \| randomi?ed.ab. \| 665337 \| \| 50 \| randomly.ab. \| 379579 \| \| 51 \| rct.ti,ab. \| 27089 \| \| 52 \| trial.ti. \| 259833 \| \| 53 \| Observational Study.pt. \| 124812 \| \| 54 \| (observ* adj3 (study or studies)).ab,ti. \| 242202 \| \| 55 \| exp Cohort Studies/ \| 2326635 \| \| 56 \| (cohort adj (study or studies)).ab,ti. \| 267823 \| \| 57 \| (cohort analy* or longitudinal or prospective* or retrospective*).ab,ti. \| 1865937 \| \| 58 \| (follow up adj (study or studies)).ab,ti. \| 53295 \| \| 59 \| (before* adj2 after*).ti,ab. \| 308685 \| \| 60 \| (pre* adj3 post*).ti,ab. \| 368404 \| \| 61 \| or/46-60 \| 4653532 \| \| 62 \| 7 and 32 and 45 and 61 \| 4383 \| \| 63 \| limit 62 to (danish or english or french or german or norwegian or swedish) \| 4295 \| | |

Web of Science Core Collection

| Interface: Clarivate Analytics  Date of Search: 11 April 2022  Number of hits: 3,605 | Field labels   - TS/Topic = title, abstract, author keywords and Keywords Plus - NEAR/x = within x words, regardless of order - * = truncation of word for alternate endings   Note: sometimes “quotation marks” are needed for single search terms to avoid automatic term mapping (lemmatization). |
| --- | --- |
| #1 TS=(("asylum seek*" OR diaspora OR emigrat* OR emigrant* OR "floating population" OR immigrat* OR immigrant* OR migrat* OR migrant* OR newcomer* OR "newly arrived" OR refugee*) OR (displac* NEAR/3 (adolescen* OR boy$ OR child* OR famil* OR father* OR forced OR girl$ OR internal* OR mass OR minor* OR mother* OR parent* OR person* OR people* OR population* OR youth* )) )  #2 TS=(((((((anxiety OR agoraphobia OR "antisocial behavio*" OR anxious* OR depression OR PTSD OR "quality of life" OR "social phobia" OR "war neuros*" OR "well-being" ) OR ((disorder* OR symptom* ) NEAR/1 (adjustment OR affective OR antisocial OR anxiety OR "child behavio*" OR depressive OR dysthymic OR mood* OR panic OR phobi* OR stress )) OR (grief OR grieving ) OR (mental* NEAR/0 (disease* OR disorder* OR health OR ill* OR "well-being" )) OR (psycho* NEAR/0 (distress* OR stress OR trauma )) OR ((adolescen* OR boy$ OR child* OR famil* OR father* OR girl$ OR minor* OR mother* OR parent* OR youth* ) NEAR/2 (conflict* OR interact* OR relation* )) OR (competence OR confidence OR parenting OR resilienc* OR "self-efficacy" OR skill* ))))) ))  #3 TS=(((((((cognitive NEAR/1 therapy) OR (desensitization NEAR/1 ("eye movement*" OR psychologic*) ) OR ( (exposure OR flooding OR implosive OR narrative OR "prolonged exposure") NEAR/0 therapy)  OR (psychotherapy NEAR/0 (brief OR dynamic OR psychodynamic OR "short-term") ) OR (intervention* OR prevent* OR program* OR rehabilitation OR therap* or treat*) OR (CBT OR "TF-CBT" OR EMDR OR ISTDP OR PDT) ))))))  #4  TS=((((("clinical trial" or randomi?ed or randomly or RCT) OR (observ* NEAR/2 (study or studies) ) OR  (cohort NEAR/0 (study or studies) ) OR ("cohort analy*" or longitudinal or prospective* or retrospective*) OR ("follow up" NEAR/0 (study or studies) ))))) OR TI=((before* NEAR/1 after*)) OR AB=((before* NEAR/1 after*)) OR TI=(pre* NEAR/2 post*) OR AB=(pre* NEAR/2 post*)  #5 #1 AND #2 AND #3 AND #4 **Refined by:** **LANGUAGES:** ( ENGLISH OR FRENCH OR DANISH OR GERMAN ) | |

APA Psychinfo (EBSCO)

| Interface: EBSCO  Date of Search: 11 April 2022  Number of hits: 1,445 | Field labels   - DE = controlled term - / = non exploded controlled term - TI AB KW = title, abstract and author keywords - Nx = within x words, regardless of order - Wx= within x words, fixed word order - * = truncation of word for alternate endings |
| --- | --- |
| \| # \| Query \| Results \| \| --- \| --- \| --- \| \| S1 \| DE "Immigration" \| 24,947 \| \| S2 \| DE "Human Migration" OR DE "Geographical Mobility" OR DE "Refugees" \| 16,858 \| \| S3 \| TI ( ("asylum seek*" OR diaspora OR emigrat* OR emigrant* OR "floating population" OR immigrat* OR immigrant* OR migrat* OR migrant* OR newcomer* OR "newly arrived" OR refugee*) ) OR AB ( ("asylum seek*" OR diaspora OR emigrat* OR emigrant* OR "floating population" OR immigrat* OR immigrant* OR migrat* OR migrant* OR newcomer* OR "newly arrived" OR refugee*) ) OR KW ( ("asylum seek*" OR diaspora OR emigrat* OR emigrant* OR "floating population" OR immigrat* OR immigrant* OR migrat* OR migrant* OR newcomer* OR "newly arrived" OR refugee*) ) \| 71,606 \| \| S4 \| TI (displac* N3 (adolescen* OR boy# OR child* OR famil* OR father* OR forced OR girl# OR internal* OR mass OR minor* OR mother* OR parent* OR person* OR people* OR population* OR youth* )) OR AB (displac* N3 (adolescen* OR boy# OR child* OR famil* OR father* OR forced OR girl# OR internal* OR mass OR minor* OR mother* OR parent* OR person* OR people* OR population* OR youth* )) OR KW (displac* N3 (adolescen* OR boy# OR child* OR famil* OR father* OR forced OR girl# OR internal* OR mass OR minor* OR mother* OR parent* OR person* OR people* OR population* OR youth* )) \| 2,172 \| \| S5 \| S1 OR S2 OR S3 OR S4 \| 75,548 \| \| S6 \| DE "Antisocial Personality Disorder" \| 9,591 \| \| S7 \| DE "Anxiety" \| 89,938 \| \| S8 \| (DE "Anxiety Disorders" OR DE "Phobias" OR DE "Acrophobia" OR DE "Agoraphobia" OR DE "Claustrophobia" OR DE "Ophidiophobia" OR DE "School Phobia" OR DE "Social Phobia" OR DE "Obsessive Compulsive Disorder" OR DE "Hoarding Disorder" OR DE "Koro" OR DE "Hoarding Behavior" OR DE "Mental Disorders" OR DE "Castration Anxiety" OR DE "Generalized Anxiety Disorder" OR DE "Panic Attack" OR DE "Panic Disorder" OR DE "Separation Anxiety Disorder" OR DE "Trichotillomania") \| 200,654 \| \| S9 \| DE "Child Behavior" \| 8,570 \| \| S10 \| DE "Depression (Emotion)" \| 26,513 \| \| S11 \| DE "Major Depression" OR DE "Anaclitic Depression" OR DE "Dysthymic Disorder" OR DE "Endogenous Depression" OR DE "Late Life Depression" OR DE "Postpartum Depression" OR DE "Reactive Depression" OR DE "Recurrent Depression" OR DE "Treatment Resistant Depression" \| 147,162 \| \| S12 \| (DE "Emotional Adjustment" OR DE "Identity Crisis" OR DE "Emotional Control" OR DE "Anger Control") \| 23,028 \| \| S13 \| DE "Mental Health" OR DE "Athlete Mental Health" OR DE "Mental Health Disparities" OR DE "Mental Status" \| 86,962 \| \| S14 \| DE "Emotional Trauma" \| 15,950 \| \| S15 \| DE "Quality of Life" \| 62,001 \| \| S16 \| (DE "Stress and Trauma Related Disorders" OR DE "Acute Stress Disorder" OR DE "Adjustment Disorders" OR DE "Attachment Disorders" OR DE "Posttraumatic Stress Disorder" OR DE "Disinhibited Social Engagement Disorder" OR DE "Complex PTSD" OR DE "DESNOS") \| 40,955 \| \| S17 \| DE "Psychological Stress" \| 9,362 \| \| S18 \| TI ( (anxiety OR agoraphobia OR "antisocial behavio*" OR anxious* OR depression OR PTSD OR "quality of life" OR "social phobia" OR "war neuros*" OR "well-being" ) ) OR AB ( (anxiety OR agoraphobia OR "antisocial behavio*" OR anxious* OR depression OR PTSD OR "quality of life" OR "social phobia" OR "war neuros*" OR "well-being" ) ) OR KW ( (anxiety OR agoraphobia OR "antisocial behavio*" OR anxious* OR depression OR PTSD OR "quality of life" OR "social phobia" OR "war neuros*" OR "well-being" ) ) \| 595,861 \| \| S19 \| TI ( ((disorder* or symptom*) N1 (adjustment or affective or antisocial or anxiety or child behavio* or depressive or dysthymic or mood* or panic or phobi* or stress)) ) OR AB ( ((disorder* or symptom*) N1 (adjustment or affective or antisocial or anxiety or child behavio* or depressive or dysthymic or mood* or panic or phobi* or stress)) ) OR KW ( ((disorder* or symptom*) N1 (adjustment or affective or antisocial or anxiety or child behavio* or depressive or dysthymic or mood* or panic or phobi* or stress)) ) \| 224,877 \| \| S20 \| TI ( (grief OR grieving) ) OR AB ( (grief OR grieving) ) OR KW ( (grief OR grieving) ) \| 15,463 \| \| S21 \| TI ( (mental* W1 (disease* OR disorder* OR health OR ill* OR "well-being" )) ) OR AB ( (mental* W1 (disease* OR disorder* OR health OR ill* OR "well-being" )) ) OR KW ( (mental* W1 (disease* OR disorder* OR health OR ill* OR "well-being" )) ) \| 305,822 \| \| S22 \| TI ( (psycho* W1 (distress* OR stress OR trauma )) ) OR AB ( (psycho* W1 (distress* OR stress OR trauma )) ) OR KW ( (psycho* W1 (distress* OR stress OR trauma )) ) \| 36,438 \| \| S23 \| DE "Parenting" OR DE "Authoritarian Parenting" OR DE "Authoritative Parenting" OR DE "Childrearing Practices" OR DE "Coparenting" OR DE "Parent Child Communication" OR DE "Parent Child Relations" OR DE "Parental Involvement" OR DE "Parenthood Status" OR DE "Parenting Skills" OR DE "Parenting Style" OR DE "Permissive Parenting" OR DE "Child Care" OR DE "Child Care Workers" OR DE "Child Day Care" OR DE "Child Self Care" OR DE "Child Discipline" OR DE "Permissive Parenting" OR DE "Physical Discipline" OR DE "Toilet Training" OR DE "Weaning" OR DE "Father Child Communication" OR DE "Mother Child Communication" OR DE "Father Child Relations" OR DE "Mother Child Relations" OR DE "Parental Attitudes" OR DE "Parental Expectations" OR DE "Parent School Relationship") OR DE "Childlessness" OR DE "Authoritarian Parenting" OR DE "Authoritative Parenting" OR DE "Permissive Parenting" \| 123,674 \| \| S24 \| DE "Self-Efficacy" \| 26,050 \| \| S25 \| TI ( ((adolescen* OR boy# OR child* OR famil* OR father* OR girl# OR minor* OR mother* OR parent* OR youth* ) N2 (conflict* OR interact* OR relation* )) ) OR AB ( ((adolescen* OR boy# OR child* OR famil* OR father* OR girl# OR minor* OR mother* OR parent* OR youth* ) N2 (conflict* OR interact* OR relation* )) ) OR KW ( ((adolescen* OR boy# OR child* OR famil* OR father* OR girl# OR minor* OR mother* OR parent* OR youth* ) N2 (conflict* OR interact* OR relation* )) ) \| 142,265 \| \| S26 \| TI ( (competence OR confidence OR parenting OR resilienc* OR "self-efficacy" OR skill* ) ) OR AB ( (competence OR confidence OR parenting OR resilienc* OR "self-efficacy" OR skill* ) ) OR KW ( (competence OR confidence OR parenting OR resilienc* OR "self-efficacy" OR skill* ) ) \| 478,754 \| \| S27 \| DE "Resilience (Psychological)" \| 17,912 \| \| S28 \| S6 OR S7 OR S8 OR S9 OR S10 OR S11 OR S12 OR S13 OR S14 OR S15 OR S16 OR S17 OR S18 OR S19 OR S20 OR S21 OR S22 OR S23 OR S24 OR S25 OR S26 OR S27 \| 1,498,967 \| \| S29 \| DE "Cognitive Behavior Therapy" OR DE "Acceptance and Commitment Therapy" OR DE "Cognitive Processing Therapy" OR DE "Prolonged Exposure Therapy" \| 24,701 \| \| S30 \| DE "Eye Movement Desensitization Therapy" \| 1,742 \| \| S31 \| DE "Health Promotion" \| 36,222 \| \| S32 \| DE "Brief Psychotherapy" \| 5,913 \| \| S33 \| DE "Psychodynamic Psychotherapy" \| 3,820 \| \| S34 \| TI ( (CBT OR "TF-CBT" OR EMDR OR ISTDP OR PDT ) ) OR AB ( (CBT OR "TF-CBT" OR EMDR OR ISTDP OR PDT ) ) OR KW ( (CBT OR "TF-CBT" OR EMDR OR ISTDP OR PDT ) ) \| 18,197 \| \| S35 \| TI (cognitive N1 therap*) OR AB (cognitive N1 therap*) OR KW (cognitive N1 therap*) \| 36,614 \| \| S36 \| TI ( (desensitization N1 ("eye movement*" OR psychologic* )) ) OR AB ( (desensitization N1 ("eye movement*" OR psychologic* )) ) OR KW ( (desensitization N1 ("eye movement*" OR psychologic* )) ) \| 1,949 \| \| S37 \| TI ( ((exposure OR flooding OR implosive OR narrative OR "prolonged exposure" ) W1 therapy ) ) OR AB ( ((exposure OR flooding OR implosive OR narrative OR "prolonged exposure" ) W1 therapy ) ) OR KW ( ((exposure OR flooding OR implosive OR narrative OR "prolonged exposure" ) W1 therapy ) ) \| 4,872 \| \| S38 \| TI ( (psychotherapy W1 (brief OR dynamic OR psychodynamic OR "short-term" )) ) OR AB ( (psychotherapy W1 (brief OR dynamic OR psychodynamic OR "short-term" )) ) OR KW ( (psychotherapy W1 (brief OR dynamic OR psychodynamic OR "short-term" )) ) \| 467 \| \| S39 \| TI ( (intervention* OR prevent* OR program* OR rehabilitation OR therap* OR treat* ) ) OR AB ( (intervention* OR prevent* OR program* OR rehabilitation OR therap* OR treat* ) ) OR KW ( (intervention* OR prevent* OR program* OR rehabilitation OR therap* OR treat* ) ) \| 1,654,355 \| \| S40 \| S29 OR S30 OR S31 OR S32 OR S33 OR S34 OR S35 OR S36 OR S37 OR S38 OR S39 \| 1,666,525 \| \| S41 \| S5 AND S28 AND S40 \| 10,138 \| \| S42 \| TI ( ("clinical trial" OR randomi#ed OR randomly OR RCT ) ) OR AB ( ("clinical trial" OR randomi#ed OR randomly OR RCT ) ) \| 171,188 \| \| S43 \| TI ( (observ* N2 (study OR studies )) ) OR AB ( (observ* N2 (study OR studies )) ) \| 27,087 \| \| S44 \| TI ( (cohort W1 (study OR studies )) ) OR AB ( (cohort W1 (study OR studies )) ) \| 26,085 \| \| S45 \| TI ( ("cohort analy*" OR longitudinal OR prospective* OR retrospective* ) ) OR AB ( ("cohort analy*" OR longitudinal OR prospective* OR retrospective* ) ) \| 231,545 \| \| S46 \| TI ( ("follow up" W1 (study OR studies )) ) OR AB ( ("follow up" W1 (study OR studies )) ) \| 14,263 \| \| S47 \| TI (before* N1 after* ) OR AB (before* N1 after* ) \| 47,664 \| \| S48 \| TI (pre* N2 post*) OR AB (pre* N2 post*) \| 86,108 \| \| S49 \| S42 OR S43 OR S44 OR S45 OR S46 OR S47 OR S48 \| 528,137 \| \| S50 \| S41 AND S49 \| 1,475 \| \| S51 \| S50 - Limiters - Language: Danish, English, French, German, Norwegian, Swedish \| 1,445 \| | |
