# Supplementary material for: The efficacy of psychological prevention, and health promotion interventions targeting psychological health, wellbeing or resilience among forced migrant children and youth: a systematic review and meta-analysis
Source: Eur Child Adolesc Psychiatry. 2024 Apr 16;34(1):123–40. doi: 10.1007/s00787-024-02424-8 (PMC11805832; doi:10.1007/s00787-024-02424-8)
Supplement: Supplementary file 4 — Supplementary file4 (DOCX 24 KB) [file 787_2024_2424_MOESM4_ESM.docx]

Supplementary Information 4

**The efficacy of psychological prevention, and health promotion interventions targeting psychological health, wellbeing or resilience among forced migrant children and youth: a systematic review and meta-analysis**

**European Child and Adolescent Psychiatry**

Clover Jack Giles ^1^, Maja Västhagen ^2^, Livia Van Leuven ^2^,

Anna Edenius^3^, Ata Ghaderi ^2^, Pia Enebrink ^2^

^1^ School of Behavioural, Social and Legal Sciences, Örebro University, Örebro, Sweden

^2^ Department of Clinical Neuroscience, Karolinska Institutet, Stockholm, Sweden

^3^ Department of Medicine, Karolinska Institutet, Stockholm, Sweden

*Corresponding author:*

Clover Jack Giles (CJG)

[clover.giles@oru.se](mailto:clover.giles@oru.se)

# Supplementary Information 4: Data Items

The following items were extracted collaboratively by a pair of reviewers.

**Publication information**

- Author
- Publication year
- Article title
- Journal

**Study characteristics**

- Study design (RCT, quasi-randomized, cohort)
- Type of randomization (individual, cluster, etc.)
- types of comparators (none, waitlist control, treatment as usual)
- Recruitment procedure (description of where recruitment occurred, and by whom)
- Risk of bias assessment

**Participant characteristics**

- Age (range and mean)
- Gender distribution
- Ethnicity
- Participant’s mother tongue
- Described status (refugee, asylum seeker, internally displaced)
- Reason for migration (war, persecution)
- Unaccompanied or accompanied
- Time in refugee camp and/or host country
- School history (years of schooling)
- Current housing situation

**Intervention and setting**

- Intervention name
- Type and level of intervention (promotion, universal, selective or indicated prevention)
- Intervention format (individual, group)
  - Number of participants per group
- Description of the integration (free text)
- Reported components in the interventions (free text)
- Information about cultural adaptations (free text)
- Classification of intervention (e.g., CBT, IPT, psychoeducation)
- Country where the intervention took place
- Context where intervention took place (e.g., school, community centre)
- Duration of the intervention
  - weeks
  - sessions
  - hours
- Number and profession of intervention leaders (clinical professional/trained member of community/translator)
- Implementation language

**Outcome data/results**

- N at enrolment
- n included in analysis
- Total attrition
- Reasons for attrition
- Intervention drop-out
- Name of outcome measure
- Outcome variable
- Statistical analysis completed
- Type of analysis (intention to treat or study completer analysis)
- Time point (post or follow-up)
- Length of follow-up

The following items were extracted independently by a pair of reviewers and then checked collaboratively.

**Outcome data/results**

- Means and standard deviations at pre-, post-/follow-up measurements
- Effect size
