# Supplementary material for: The efficacy of psychological prevention, and health promotion interventions targeting psychological health, wellbeing or resilience among forced migrant children and youth: a systematic review and meta-analysis
Source: Eur Child Adolesc Psychiatry. 2024 Apr 16;34(1):123–40. doi: 10.1007/s00787-024-02424-8 (PMC11805832; doi:10.1007/s00787-024-02424-8)
Supplement: Supplementary file 5 — Supplementary file5 (DOCX 56 KB) [file 787_2024_2424_MOESM5_ESM.docx]

Supplementary Information 5

**The efficacy of psychological prevention, and health promotion interventions targeting psychological health, wellbeing or resilience among forced migrant children and youth: a systematic review and meta-analysis**

**European Child and Adolescent Psychiatry**

Clover Jack Giles ^1^, Maja Västhagen ^2^, Livia Van Leuven ^2^,

Anna Edenius^3^, Ata Ghaderi ^2^, Pia Enebrink ^2^

^1^ School of Behavioural, Social and Legal Sciences, Örebro University, Örebro, Sweden

^2^ Department of Clinical Neuroscience, Karolinska Institutet, Stockholm, Sweden

^3^ Department of Medicine, Karolinska Institutet, Stockholm, Sweden

*Corresponding author:*

Clover Jack Giles (CJG)

[clover.giles@oru.se](mailto:clover.giles@oru.se)

# Supplementary Information 5: Certainty of Evidence Using GRADE

**Author(s):** REMOVED FOR BLIND REVIEW

**Question:** Promotive and preventative psychosocial interventions compared to all comparators for prevention of psychological ill health and promotion of mental health and wellbeing among forced migrant youth

**Setting:** Non-clinical settings

| **Certainty assessment** | | | | | | | **Certainty** |
| --- | --- | --- | --- | --- | --- | --- | --- |
| **№ of studies** | **Study design** | **Risk of bias** | **Inconsistency** | **Indirectness** | **Imprecision** | **Other considerations** |  |
| **Child rated between-group depression (assessed with: APAI Acholi Psychosocial Assessment Instrument, MFQ Moods and Feelings Questionnaire, DSRS Birleson Depression Self-Rating Scale)**[1-3] | | | | | | | |
| 3 | randomised trials | serious^a^ | serious^b^ | not serious | serious^c^ | none | ⨁◯◯◯ Very low |
| **Child rated within-group depression (assessed with: APAI Acholi Psychosocial Assessment Instrument, DSRC-C Depression Self rating scale for children, HQ-9 Patient Health Questionnaire, DSRS Birleson Depression Self-Rating Scale, CES-DC Center for Epidemiological Studies Depression Scale for Children, CDI Children's Depression Inventory, HSCL-25 Hopkins symptom checklist Depression, MFQ Moods and feeling Questionnaire)** [1, 2, 4-11] | | | | | | | |
| 10 | observational studies | very serious^d^ | serious^e^ | not serious | not serious^f^ | none | ⨁◯◯◯ Very low |
| **Child rated within-group Post Traumatic Stress Syndrome (assessed with: UCLA-PTSD-RI, R-IES Revised impact of events scale, CPTS-RI The child post-traumatic stress – reaction index, TGIC Traumatic grief inventory for children, CRIES-13 Childrens Revised Impacts of Events Scale, CATS Child and Adolescent Trauma Scale, CPTSD-RI Child Post Traumatic Stress Reaction Index)** [2, 4, 6, 10, 12-15] | | | | | | | |
| 8 | observational studies | very serious^g^ | serious^h^ | not serious | not serious^i^ | none | ⨁◯◯◯ Very low |
| **Child rated within group Anxiety (assessed with: GAD-7 Generalized Anxiety Disorder-7, Revised children's Manifest Anxiety Scale, SCAS Spence children's anxiety scale, HSCL-25 Hopkins symptom checklist Anxiety subscales, State-Trait Anxiety Scale)** [5, 6, 9, 11, 13] | | | | | | | |
| 5 | observational studies | serious^j^ | serious^k^ | not serious | not serious^l^ | none | ⨁⨁◯◯ Low |
| **Parent rated within group Emotional and Behavioural Difficulties (assessed with: SDQ Strengths and Difficulties Questionnaire)** [2, 12] | | | | | | | |
| 2 | observational studies | serious^m^ | very serious^n^ | serious^o^ | very serious^p^ | none | ⨁◯◯◯ Very low |
| **Teacher rated within group Emotional and Behavioural Difficulties (assessed with: SDQ Strengths and Difficulties Questionnaire)** [6, 9] | | | | | | | |
| 2 | observational studies | serious^q^ | not serious | serious^r^ | serious^s^ | none | ⨁◯◯◯ Very low |

#### Explanations

a. Bolton et al. (2007), Ooi et al. (2016), Tubbs Dolan et al. (2022). All studies have some risk of bias due to non-blinded participants/assessors, and lack of pre-registered analysis plan. Ooi and Tubbs Dolan; the interventions were not implemented as planned because of practical challenges in the implementation context.

b. Point estimate for Bolton et al. (2007; Creative Play and Interpersonal Therapy – Group) overlaps only with the confidence interval for Ooi et al (2016). Considerable variation in confidence intervals for individual studies. Statistical measures of heterogeneity are substantial, although heterogeneity may be explained to some extent by differences in intervention characteristics.

c. The combined confidence interval is narrow, however it crosses 0, suggesting risk for both positive and negative effects. According to reported power calculations the sample sizes in Bolton et al. (2007; Creative Play and Interpersonal Therapy – Group) conformed to indications, while Ooi et al (2016) was slightly lower that indicated. Tubbs Dolan et al. (2022) had a very large sample size (n = 4784).

d. Several pre-post studies (Cardeli et al., 2020; Doumit et al., 2020; Fox et al., 2005; Quinlan et al., 2016; and Ugurlu et al., 2016) have serious risk of bias due to lack of control of any potentially confounding variables. All studies have some risk of bias due to non-blinded participants/assessors. Some studies also had increased risk of bias due to lack of drop-out analyses, lack of pre-registered analysis plan and variations in interventions implemented due to challenges the implementation context.

e. Four studies do not overlap with the remainder (Bolton et al., 2007, Interpersonal Therapy – Group; Foka et al., 2021; Fox et al., 2005; and Ugurlu et al., 2016). All four studies show larger positive effects than the remaining studies in the analysis which may be explained to some extent by intervention characteristics. There is variation in confidence intervals for individual studies and statistical measures of heterogeneity are substantial.

f. The combined confidence interval is narrow, and does not include 0, suggesting that small positive effects are likely. The sample sizes in the included studies vary considerably (R = 15 to 105) and power estimations were not reported in many cases. However, the small sample sizes may however be explained by the nature of the samples and intervention contexts.

g. Three studies (Cardeli et al., 2020; Garoff et al., 2018; and Pfeiffer and Goldbeck, 2017) have serious risk of bias due to lack of control of any potentially confounding variables. All studies have some risk of bias due to non-blinded participants/assessors.

h. Confidence intervals of most studies overlap. There is considerable variation in confidence intervals for individual studies and statistical measures of heterogeneity are substantial. One study (Garoff et al., 2018) may markedly contribute to heterogeneity as there is little overlap in its confidence intervals with the remaining studies and is the only study with a point estimate < 0.

i. The combined confidence interval is narrow, and does not include 0, suggesting that small positive effects are likely. The sample sizes in the included studies are small (*R* = 12 to 47) and power estimations were not reported in many cases. However, the small sample sizes may be explained by the nature of the samples and intervention contexts.

j. Three studies (Doumit et al., 2020; Quinlan et al., 2016, and Ugurlu et al., 2016) were assessed to have serious risk of bias due to lack of control of any potentially confounding variables. Doumit also has serious risk of bias due to lack of controlled for drop-out. All studies have some risk of bias due to non-blinded participants/assessors.

k. Point estimates overlap with the confidence intervals for most studies except Ugurlu et al. (2016), where the point estimate overlaps with the confidence interval of only one study. Measures of statistical heterogeneity were substantial.

l. The combined confidence interval is narrow, and does not include 0, suggesting that small positive effects are likely. The sample sizes in the included studies are small (*R* = 15 to 64) and power estimations were not reported in many cases. However, the small sample sizes may be explained by the nature of the samples and intervention contexts.

m. One study (Garoff et al., 2018) has serious risk of bias due to lack of control of any potentially confounding variables. Both studies have some risk of bias due to non-blinded participants/assessors.

n. There is little overlap between point estimates and confidence intervals between the two included studies, and measures of statistical heterogeneity are substantial.

o. The measures is a parent/caregiver report of child behaviour and symptoms.

p. The combined point estimate is broad and includes 0, and both studies have small sample sizes.

q. One study (Quinlan et al., 2016) has serious risk of bias due to lack of control of any potentially confounding variables. Both studies have some risk of bias due to non-blinded participants/assessors.

r. The measures is a teacher report of child behaviour and symptoms.

s. The combined point estimate is broad but does not include 0, and both studies have small sample sizes.

**References**

1. Bolton, P., et al., *Interventions for depression symptoms among adolescent survivors of war and displacement in northern Uganda: a randomized controlled trial.* Jama, 2007. **298**(5): p. 519-27.

2. Ooi, C.S., et al., *The Efficacy of a Group Cognitive Behavioral Therapy for War-Affected Young Migrants Living in Australia: A Cluster Randomized Controlled Trial.* Frontiers in psychology, 2016. **7**: p. 1641.

3. Tubbs Dolan, C., et al., *Supporting Syrian Refugee Children’s Academic and Social-Emotional Learning in National Education Systems: A Cluster Randomized Controlled Trial of Nonformal Remedial Support and Mindfulness Programs in Lebanon.* American Educational Research Journal, 2021. **59**(3): p. 419-460.

4. Cardeli, E., et al., *Bhutanese Refugee Youth: The Importance of Assessing and Addressing Psychosocial Needs in a School Setting.* The Journal of school health, 2020. **90**(9): p. 731-742.

5. Doumit, R., C. Kazandjian, and L.K. Militello, *COPE for Adolescent Syrian Refugees in Lebanon: A Brief Cognitive-Behavioral Skill-Building Intervention to Improve Quality of Life and Promote Positive Mental Health.* Clinical Nursing Research, 2020. **29**(4): p. 226-234.

6. Ehntholt, K.A., P.A. Smith, and W. Yule, *School-based Cognitive-Behavioural Therapy Group Intervention for Refugee Children who have Experienced War-related Trauma.* Clinical Child Psychology and Psychiatry, 2005. **10**: p. 235-250.

7. Foka, S., et al., *Promoting well-being in refugee children: An exploratory controlled trial of a positive psychology intervention delivered in Greek refugee camps.* Development and psychopathology, 2021. **33**(1): p. 87-95.

8. Fox, P.G., et al., *Southeast Asian refugee children: a school-based mental health intervention.* Int J Psychiatr Nurs Res, 2005. **11**(1): p. 1227-36.

9. Quinlan, R., et al., *Evaluation of a school-based creative arts therapy program for adolescents from refugee backgrounds.* The Arts in Psychotherapy, 2016. **47**: p. 72-78.

10. Thabet, A.A., P. Vostanis, and K. Karim, *Group crisis intervention for children during ongoing war conflict.* European Child and Adolescent Psychiatry, 2005. **14**(5): p. 262-269.

11. Ugurlu, N., L. Akca, and C. Acarturk, *An art therapy intervention for symptoms of post-traumatic stress, depression and anxiety among Syrian refugee children.* Vulnerable Children and Youth Studies, 2016. **11**(2): p. 89-102.

12. Garoff, F., S. Kangaslampi, and K. Peltonen, *Development and implementation of a group based mental health intervention for unaccompanied minors.* Scand J Psychol, 2019. **60**(1): p. 7-15.

13. Gormez, V., et al., *Evaluation of a school-based, teacher-delivered psychological intervention group program for trauma-affected Syrian refugee children in Istanbul, Turkey.* Psychiatry and Clinical Psychopharmacology, 2017. **27**(2): p. 125-131.

14. Kalantari, M., et al., *Efficacy of writing for recovery on traumatic grief symptoms of Afghani refugee bereaved adolescents: a randomized control trial.* Omega, 2012. **65**(2): p. 139-50.

15. Pfeiffer, E. and L. Goldbeck, *Evaluation of a Trauma-Focused Group Intervention for Unaccompanied Young Refugees: A Pilot Study.* Journal of traumatic stress, 2017. **30**(5): p. 531-536.
