# Supplementary material for: The efficacy of psychological prevention, and health promotion interventions targeting psychological health, wellbeing or resilience among forced migrant children and youth: a systematic review and meta-analysis
Source: Eur Child Adolesc Psychiatry. 2024 Apr 16;34(1):123–40. doi: 10.1007/s00787-024-02424-8 (PMC11805832; doi:10.1007/s00787-024-02424-8)
Supplement: Supplementary file 6 — Supplementary file6 (DOCX 78 KB) [file 787_2024_2424_MOESM6_ESM.docx]

Supplementary Information 6

**The efficacy of psychological prevention, and health promotion interventions targeting psychological health, wellbeing or resilience among forced migrant children and youth: a systematic review and meta-analysis**

**European Child and Adolescent Psychiatry**

Clover Jack Giles ^1^, Maja Västhagen ^2^, Livia Van Leuven ^2^,

Anna Edenius^3^, Ata Ghaderi ^2^, Pia Enebrink ^2^

^1^ School of Behavioural, Social and Legal Sciences, Örebro University, Örebro, Sweden

^2^ Department of Clinical Neuroscience, Karolinska Institutet, Stockholm, Sweden

^3^ Department of Medicine, Karolinska Institutet, Stockholm, Sweden

*Corresponding author:*

Clover Jack Giles (CJG)

[clover.giles@oru.se](mailto:clover.giles@oru.se)

# Supplementary Information 6: Reason for Exclusion

**Table 1**

*Studies Considered for Inclusion After Full Text Review but Later Excluded, N, search number, and Reason for Exclusion*

| Author and year | Search nr. | Reason for exclusion |
| --- | --- | --- |
| Arabacioglu & Bagceli Kahraman, 2020 | 2 | Outcome not eligible for this meta-analysis |
| Baggerly & Corbin, 2021 | 2 | Parents also included (wrong population) |
| Baker & Jones, 2006 | Reference list | Outcome not eligible for this meta-analysis |
| Erdemir, 2021 | 2 | Outcome not eligible for this meta-analysis |
| Gupta & Zimmer, 2008 | 1 | Incomplete data (no SD reported) |
| Im et al., 2018 | 2 | Included participants >18 (wrong population) |
| Khawaja & Ramirez, 2019 | 2 | Included participants >18 (wrong population) |
| Kneer et al., 2019 | 2 | No validated measures |
| Lawrence & Falaye, 2020 | 1 | Parents also included (wrong population) |
| Murray et al., 2018 | 1 | Parents also included (wrong population) |
| Pfeiffer et al., 2018 | 1 | Included participants >18 (wrong population) |
| Sarkadi et al., 2018 | 1 | Parents also included (wrong population) |
| Scheiber et al., 2019 | 1 | Non-parametric data |
| Staaehr, 2001 | 2 | Parents also included (wrong population) |

**Complete list of studies excluded from full text review**

In the spreadsheet used during the full text inclusion-exclusion process completed after reviewing abstracts in Rayyan, reason for exclusion was recorded from left to right in the following order: wrong population (not forced migrant children or youth), wrong intervention (not a psychological intervention on an universal or selective level), wrong study design (not a RCT or pre-post intervention study), wrong outcomes (none of the primary or secondary outcomes), too few participants (*n* < 10), secondary analysis, no access to data, no validated measures, wrong data type, and wrong publication (such as a thesis or study protocol). While the excluded studies may have been excluded for several reasons, they are ordered here by the first appearing in the spreadsheet sequence.

**Wrong population**

Abbott, A. (2016). The mental-health crisis among migrants. *Nature, 538*(7624), 158-160.

Abuelezam, N. N., El-Sayed, A. M., & Galea, S. (2018). The Health of Arab Americans in the United States: An Updated Comprehensive Literature Review. *Frontiers in public health, 6*, 262.

Abuelezam, N. N., & Fontenot, H. B. (2017). Depression Among Arab American and Arab Immigrant Women in the United States. *Nursing for women's health, 21*(5), 395-399.

Acarturk, C., Konuk, E., Cetinkaya, M., Senay, I., Sijbrandij, M., Cuijpers, P., & Aker, T. (2015). EMDR for Syrian refugees with posttraumatic stress disorder symptoms: results of a pilot randomized controlled trial. *European journal of psychotraumatology, 6*, 27414.

Acarturk, C., Konuk, E., Cetinkaya, M., Senay, I., Sijbrandij, M., Gulen, B., & Cuijpers, P. (2016). The efficacy of eye movement desensitization and reprocessing for post-traumatic stress disorder and depression among Syrian refugees: results of a randomized controlled trial. *Psychological Medicine, 46*(12), 2583-2593.

Acarturk, Z. C., Abuhamdeh, S., Jalal, B., Unaldi, N., Alyanak, B., Cetinkaya, M., . . . Hinton, D. (2019). Culturally Adapted Transdiagnostic CBT for SSRI-Resistant Turkish Adolescents: A Pilot Study. *American Journal of Orthopsychiatry, 89*(2), 222-227.

Adenauer, H., Catani, C., Gola, H., Keil, J., Ruf, M., Schauer, M., & Neuner, F. (2011). Narrative exposure therapy for PTSD increases top-down processing of aversive stimuli--evidence from a randomized controlled treatment trial. *BMC neuroscience, 12*, 127.

Akhtar, A., Malik, A., Ghatasheh, M., Aqel, I. S., Habashneh, R., Dawson, K. S., Watts, S., Jordans, M., Brown, F., Sijbrandij, M., Cujipers, P., Bryant, R. (2021). Feasibility trial of a brief scalable psychological intervention for Syrian refugee adolescents in Jordan. *Eur J Psychotraumatol, 12*(1). doi:10.1080/20008198.2021.1901408

Alegria, M., Falgas-Bague, I., Collazos, F., Carmona Camacho, R., Lapatin Markle, S., Wang, Y., . . . Shrout, P. E. (2019). Evaluation of the Integrated Intervention for Dual Problems and Early Action Among Latino Immigrants With Co-occurring Mental Health and Substance Misuse Symptoms: A Randomized Clinical Trial. *JAMA network open, 2*(1), e186927.

Alsheikh Ali, A. S. a. S. (2020). Efficiency of Intervention Counseling Program on the Enhanced Psychological Well-being and Reduced Post-traumatic Stress Disorder Symptoms Among Syrian Women Refugee Survivors. *Clinical practice and epidemiology in mental health : CP & EMH, 16*, 134-141.

Alsmadi, A. M., Tawalbeh, L. I., Gammoh, O. S., Shawagfeh, M. Q., Zalloum, W., Ashour, A., & Attarian, H. (2018). The effect of Ginkgo biloba and psycho-education on stress, anxiety and fatigue among refugees. *Proceedings of Singapore Healthcare, 27*(1), 26-32.

Annan, J., Sim, A., Puffer, E. S., Salhi, C., & Betancourt, T. S. (2017). Improving Mental Health Outcomes of Burmese Migrant and Displaced Children in Thailand: a Community-Based Randomized Controlled Trial of a Parenting and Family Skills Intervention. *Prevention science : the official journal of the Society for Prevention Research, 18*(7), 793-803.

Asghar, K., Mayevskaya, Y., Sommer, M., Razzaque, A., Laird, B., Khan, Y., . . . Stark, L. (2018). Promoting Adolescent Girls' Well-Being in Pakistan: a Mixed-Methods Study of Change Over Time, Feasibility, and Acceptability, of the COMPASS Program. *Prevention science : the official journal of the Society for Prevention Research, 19*(8), 1030-1042.

Askovic, M., Watters, A. J., Coello, M., Aroche, J., Harris, A. W. F., & Kropotov, J. (2020). Evaluation of Neurofeedback for Posttraumatic Stress Disorder Related to Refugee Experiences Using Self-Report and Cognitive ERP Measures. *Clinical EEG and neuroscience, 51*(2), 79-86.

Ayala, G. X., Elder, J. P., Campbell, N. R., Arredondo, E., Baquero, B., Crespo, N. C., & Slymen, D. J. (2010). Longitudinal intervention effects on parenting of the Aventuras para Ninos study. *American journal of preventive medicine, 38*(2), 154-162.

Ayoughi, S., Missmahl, I., Weierstall, R., & Elbert, T. (2012). Provision of mental health services in resource-poor settings: a randomised trial comparing counselling with routine medical treatment in North Afghanistan (Mazar-e-Sharif). *BMC psychiatry, 12*.

Ballard, J., Wieling, E., & Forgatch, M. (2018). Feasibility of Implementation of a Parenting Intervention with Karen Refugees Resettled from Burma. *Journal of marital and family therapy, 44*(2), 220-234. doi:[10.1111/jmft.12286](http://dx.doi.org/10.1111/jmft.12286)

Baggerly, J. N., & Corbin, T. (2021). Group counseling for southeast Asian refugee children with trauma symptoms: Pilot study results and practical guidelines. *Journal of Child and Adolescent Counseling, 7*(2), 87-99.

Barrett, P. M., Sonderegger, R., & Xenos, S. (2003). Using FRIENDS to combat anxiety and adjustment problems among young migrants to Australia: A national trial. *Clinical Child Psychology and Psychiatry, 8*(2), 241-260.

Barnett, M. L., Davis, E. M., Callejas, L. M., White, J. V., Acevedo-Polakovich, I. D., Niec, L. N., & Jent, J. F. (2016). The development and evaluation of a natural helpers' training program to increase the engagement of urban, Latina/o families in parent-child interaction therapy. *Children and Youth Services Review, 65*, 17-25.

Barwick, M., Urajnik, D., Sumner, L., Cohen, S., Reid, G., Engel, K., & Moore, J. E. (2013). Profiles and service utilization for children accessing a mental health walk-in clinic versus usual care. *Journal of evidence-based social work, 10*(4), 338-352.

Bauby, C., Dandres, A. M., & Lejeune, C. (2010). [Gennevilliers parents-babies unit: PMI-pedopsychiatrist partnership]. *Unite parents-bebes de Gennevilliers: partenariat PMI-pedopsychiatrie., 17*(6), 624-625.

Beck, B. D., Messel, C., Meyer, S. L., Cordtz, T. O., Sogaard, U., Simonsen, E., & Moe, T. (2018). Feasibility of trauma-focused guided imagery and music with adult refugees diagnosed with PTSD: A pilot study. *Nordic Journal of Music Therapy, 27*(1), 67-86.

Beeber, L. S., Lewis, V. S., Cooper, C., Maxwell, L., & Sandelowski, M. (2009). Meeting the "Now" Need: PMH-APRN-- Interpreter Teams Provide In-Home Mental Health Intervention for Depressed Latina Mothers With Limited English Proficiency. *Journal of the American Psychiatric Nurses Association, 15*(4), 249-259.

Bennett-Conroy, W. (2012). Engaging parents of eighth grade students in parent-teacher bidirectional communication. *The School Community Journal, 22*(2), 87-110.

Bentley, J. A., Feeny, N. C., Dolezal, M. L., Klein, A., Marks, L. H., Graham, B., & Zoellner, L. A. (2021). Islamic Trauma Healing: Integrating Faith and Empirically Supported Principles in a Community-Based Program. *Cognitive and Behavioral Practice, 28*(2), 167-192.

Bernardi, J., Dahiya, M., & Jobson, L. (2019). Culturally modified cognitive processing therapy for Karen refugees with posttraumatic stress disorder: A pilot study. *Clinical psychology & psychotherapy, 26*(5), 531-539.

Bernhardt, L. J., Lin, S., Swegman, C., Sellke, R., Vu, A., Solomon, B. S., & Cuneo, C. N. (2019). The Refugee Health Partnership: A Longitudinal Experiential Medical Student Curriculum in Refugee/Asylee Health. *Academic medicine : journal of the Association of American Medical Colleges, 94*(4), 544-549.

Bernstein, K., Park, S. Y., Hahm, S., Lee, Y. N., Seo, J. Y., & Nokes, K. M. (2016). Efficacy of a Culturally Tailored Therapeutic Intervention Program for Community Dwelling Depressed Korean American Women: A Non-Randomized Quasi-Experimental Design Study. *Archives of Psychiatric Nursing, 30*(1), 19-26.

Betancourt, T. S., Berent, J. M., Freeman, J., Frounfelker, R. L., Brennan, R. T., Abdi, S., Maalim, A., Abdi, A., Mishra, T., Gautam, B., Creswell, J. W., Beardslee, W. R. (2020). Family-Based Mental Health Promotion for Somali Bantu and Bhutanese Refugees: Feasibility and Acceptability Trial. *The Journal of adolescent health : official publication of the Society for Adolescent Medicine, 66*(3), 336-344. doi:[10.1016/j.jadohealth.2019.08.023](https://doi.org/10.1016/j.jadohealth.2019.08.023)

Betancourt, T. S., Newnham, E. A., Brennan, R. T., Verdeli, H., Borisova, I., Neugebauer, R., . . . Bolton, P. (2012). Moderators of treatment effectiveness for war-affected youth with depression in northern Uganda. *The Journal of adolescent health : official publication of the Society for Adolescent Medicine, 51*(6), 544-550.

Betancourt, T. S., Yudron, M., Wheaton, W., & Smith-Fawzi, M. C. (2012). Caregiver and adolescent mental health in Ethiopian Kunama refugees participating in an emergency education program. *The Journal of adolescent health : official publication of the Society for Adolescent Medicine, 51*(4), 357-365.

Bhavsar, V., Jannesari, S., McGuire, P., MacCabe, J. H., Das-Munshi, J., Bhugra, D., . . . Hatch, S. L. (2021). The association of migration and ethnicity with use of the Improving Access to Psychological Treatment (IAPT) programme: a general population cohort study. *Social psychiatry and psychiatric epidemiology*.

Birman, D., Beehler, S., Harris, E. M., Everson, M. L., Batia, K., Liautaud, J., . . . Cappella, E. (2008). International family, adult, and child enhancement services (FACES): A community-based comprehensive services model for refugee children in resettlement. *American Journal of Orthopsychiatry, 78*(1), 121-132.

Bjorknes, R., Kjobli, J., Manger, T., & Jakobsen, R. (2012). Parent training among ethnic minorities: Parenting practices as mediators of change in child conduct problems. *Family Relations: An Interdisciplinary Journal of Applied Family Studies, 61*(1), 101-114.

Bjorknes, R., Larsen, M., Gwanzura-Ottemoller, F., & Kjobli, J. (2015). Exploring mental distress among immigrant mothers participating in parent training. *Children and Youth Services Review, 51*, 10-17. doi:10.1016/j.childyouth.2015.01.018

Bjorknes, R., & Manger, T. (2013). Can parent training alter parent practice and reduce conduct problems in ethnic minority children? A randomized controlled trial. *Prevention science: the official journal of the Society for Prevention Research, 14*(1), 52-63. doi:[10.1007/s11121-012-0299-9](https://doi.org/10.1007/s11121-012-0299-9)

Björn, G. J., Bodén, C., Sydsjö, G., & Gustafsson, P. A. (2013). Brief family therapy for refugee children. *The Family Journal, 21*(3), 272-278.

Blanchet, K., Ramesh, A., Frison, S., Warren, E., Hossain, M., Smith, J., . . . Roberts, B. (2017). Evidence on public health interventions in humanitarian crises. *Lancet, 390*(10109), 2287-2296.

Blom, M. B. J., Hoek, H. W., Spinhoven, P., Hoencamp, E., Haffmans, P. M. J., & van Dyck, R. (2010). Treatment of Depression in Patients from Ethnic Minority Groups in the Netherlands. *Transcultural Psychiatry, 47*(3), 473-490.

Boehnlein, J. K., Kinzie, J. D., Sekiya, U., Riley, C., Pou, K., & Rosborough, B. (2004). A ten-year treatment outcome study of traumatized Cambodian refugees. *The Journal of nervous and mental disease, 192*(10), 658-663.

Boge, K., Karnouk, C., Hahn, E., Schneider, F., Habel, U., Banaschewski, T., . . . Bajbouj, M. (2020). Mental health in refugees and asylum seekers (MEHIRA): study design and methodology of a prospective multicentre randomized controlled trail investigating the effects of a stepped and collaborative care model. *European archives of psychiatry and clinical neuroscience, 270*(1), 95-106.

Bolton, P., Bass, J. K., Zangana, G. A. S., Kamal, T., Murray, S. M., Kaysen, D., . . . Rosenblum, M. (2014). A randomized controlled trial of mental health interventions for survivors of systematic violence in Kurdistan, Northern Iraq. *BMC psychiatry, 14*.

Bolton, P., Lee, C., Haroz, E. E., Murray, L., Dorsey, S., Robinson, C., . . . Bass, J. (2014). A transdiagnostic community-based mental health treatment for comorbid disorders: development and outcomes of a randomized controlled trial among Burmese refugees in Thailand. *PLoS medicine, 11*(11), e1001757.

Boyce, L. K., Innocenti, M. S., Roggman, L. A., Jump Norman, V. K., & Ortiz, E. (2010). Telling stories and making books: Evidence for an intervention to help parents in migrant Head Start families support their children's language and literacy. *Early Education and Development, 21*(3), 343-371.

Boyd, A. T., Cookson, S. T., Anderson, M., Bilukha, O. O., Brennan, M., Handzel, T., . . . Gerber, M. (2017). Centers for Disease Control and Prevention Public Health Response to Humanitarian Emergencies, 2007-2016. *Emerging Infectious Diseases, 23*, S196-S202.

Bradley, G. M., Couchman, G. M., Perlesz, A., Nguyen, A. T., Singh, B., & Riess, C. (2006). Multiple-family group treatment for English- and Vietnamese-speaking families living with schizophrenia. *Psychiatric services (Washington, D.C.), 57*(4), 521-530.

Brown, F. L., Carswell, K., Augustinavicius, J., Adaku, A., Leku, M. R., White, R. G., . . . Tol, W. A. (2018). Self Help Plus: study protocol for a cluster-randomised controlled trial of guided self-help with South Sudanese refugee women in Uganda. *Global mental health (Cambridge, England), 5*, e27.

Bruhn, M., Rees, S., Mohsin, M., Silove, D., & Carlsson, J. (2018). The Range and Impact of Postmigration Stressors During Treatment of Trauma-Affected Refugees. *Journal of Nervous and Mental Disease, 206*(1), 61-68.

Bruno, W., Kitamura, A., Najjar, S., Seita, A., & Al-Delaimy, W. K. (2019). Assessment of mental health and psycho-social support pilot program's effect on intended stigmatizing behavior at the Saftawi Health Center, Gaza: a cross-sectional study. *Journal of mental health (Abingdon, England), 28*(4), 436-442.

Bugental, D. B., & Schwartz, A. (2009). A cognitive approach to child mistreatment prevention among medically at-risk infants. *Developmental psychology, 45*(1), 284-288.

Buhmann, C., Andersen, I., Mortensen, E. L., Ryberg, J., Nordentoft, M., & Ekstrom, M. (2015). Cognitive behavioral psychotherapeutic treatment at a psychiatric trauma clinic for refugees: description and evaluation. *Torture : quarterly journal on rehabilitation of torture victims and prevention of torture, 25*(1), 17-32.

Buhmann, C., Mortensen, E. L., Nordentoft, M., Ryberg, J., & Ekstrom, M. (2015). Follow-up study of the treatment outcomes at a psychiatric trauma clinic for refugees. *Torture : quarterly journal on rehabilitation of torture victims and prevention of torture, 25*(1), 1-16.

Buhmann, C. B. (2014). Traumatized refugees: morbidity, treatment and predictors of outcome. *Danish medical journal, 61*(8), B4871.

Buhmann, C. B., Nordentoft, M., Ekstroem, M., Carlsson, J., & Mortensen, E. L. (2016). The effect of flexible cognitive-behavioural therapy and medical treatment, including antidepressants on post-traumatic stress disorder and depression in traumatised refugees: pragmatic randomised controlled clinical trial. *The British journal of psychiatry : the journal of mental science, 208*(3), 252-259.

Buhmann, C. B., Nordentoft, M., Ekstroem, M., Carlsson, J., & Mortensen, E. L. (2018). Long-term treatment effect of trauma-affected refugees with flexible cognitive behavioural therapy and antidepressants. *Psychiatry research, 264*, 217-223.

Burruss, N. C., Shaltout, Y., Hamilton, C. T., Oberti, D., Linton, J. M., & Brown, C. L. (2021). Arts-based therapy: a pilot program for immigrant and refugee children. *Vulnerable Children and Youth Studies, 16*(3), 253-258.

Carlsson, J., Sonne, C., Vindbjerg, E., & Mortensen, E. L. (2018). Stress management versus cognitive restructuring in trauma-affected refugees-A pragmatic randomised study. *Psychiatry research, 266*, 116-123.

Carlsson, J. M., Mortensen, E. L., & Kastrup, M. (2005). A follow-up study of mental health and health-related quality of life in tortured refugees in multidisciplinary treatment. *The Journal of nervous and mental disease, 193*(10), 651-657.

Carlsson, J. M., Olsen, D. R., Kastrup, M., & Mortensen, E. L. (2010). Late mental health changes in tortured refugees in multidisciplinary treatment. *The Journal of nervous and mental disease, 198*(11), 824-828.

Catani, C., Kohiladevy, M., Ruf, M., Schauer, E., Elbert, T., & Neuner, F. (2009). Treating children traumatized by war and Tsunami: a comparison between exposure therapy and meditation-relaxation in North-East Sri Lanka. *BMC psychiatry, 9*, 22.

Ceballos, P. L., & Bratton, S. C. (2010). Empowering Latino families: Effects of a culturally responsive intervention for low-income immigrant Latino parents on children's behaviors and parental stress. *Psychology in the Schools, 47*(8), 761-775.

Chiu, S. J., Lin, I. F., Chou, Y. T., & Chien, L. Y. (2020). Family quality of life among Taiwanese children with developmental delay before and after early intervention. *Journal of intellectual disability research : JIDR, 64*(8), 589-601.

Colombari Figueroa, S., Stafford, R. S., Heaney, C. A., & Rosas, L. G. (2018). The Effect of a Behavioral Weight-Loss Intervention on Depressive Symptoms Among Latino Immigrants in a Randomized Controlled Trial. *Journal of immigrant and minority health, 20*(5), 1182-1189.

Cowell, J. M., McNaughton, D., Ailey, S., Gross, D., & Fogg, L. (2009). Clinical Trail Outcomes of the Mexican American Problem Solving Program (MAPS). *Hispanic health care international : the official journal of the National Association of Hispanic Nurses, 7*(4), 179-189.

Culhane-Pera, K. A., Peterson, K. A., Crain, A. L., Center, B. A., Lee, M., Her, B., & Xiong, T. (2005). Group visits for Hmong adults with type 2 diabetes mellitus: A pre-post analysis. *Journal of Health Care for the Poor and Underserved, 16*(2), 315-327.

Cunningham, C. E., Bremner, R., & Boyle, M. (1995). Large group community-based parenting programs for families of preschoolers at risk for disruptive behaviour disorders: utilization, cost effectiveness, and outcome. *Journal of child psychology and psychiatry, and allied disciplines, 36*(7), 1141-1159.

d'Ardenne, P., Ruaro, L., Cestari, L., Fakhoury, W., & Priebe, S. (2007). Does interpreter-mediated CBT with traumatized refugee people work? A comparison of patient outcomes in East London. *Behavioural and Cognitive Psychotherapy, 35*(3), 293-301.

Daou, K., Daou, L., & Cousineau-Perusse, M. (2022). A family-based intervention for refugee children. *International Journal of Social Welfare, 31*(1), 56-65.

Davey, H. L., Tough, S. C., Adair, C. E., & Benzies, K. M. (2011). Risk Factors for Sub-Clinical and Major Postpartum Depression Among a Community Cohort of Canadian Women. *Maternal and Child Health Journal, 15*(7), 866-875.

de Graaff, A. M., Cuijpers, P., Acarturk, C., Bryant, R., Burchert, S., Fuhr, D. C., . . . Sijbrandij, M. (2020). Effectiveness of a peer-refugee delivered psychological intervention to reduce psychological distress among adult Syrian refugees in the Netherlands: study protocol. *European journal of psychotraumatology, 11*(1), 1694347.

de Graaff, A. M., Cuijpers, P., McDaid, D., Park, A., Woodward, A., Bryant, R. A., . . . Consortium, S. (2020). Peer-provided Problem Management Plus (PM plus ) for adult Syrian refugees: a pilot randomised controlled trial on effectiveness and cost-effectiveness. *Epidemiology and Psychiatric Sciences, 29*.

De La Rosa-Lopes, G. M. F. (2018). *A group intervention for children who have experienced immigration-related family separation: A mixed-methods investigation.* (79). ProQuest Information & Learning.

Demezier, D. (2021). *Biculturalism, familism, and parenting styles of Haitian parents with juvenile youth: Impact of a family-based intervention.* (82). ProQuest Information & Learning,

Drozdek, B., Kamperman, A. M., Bolwerk, N., Tol, W. A., & Kleber, R. J. (2012). Group therapy with male asylum seekers and refugees with posttraumatic stress disorder: a controlled comparison cohort study of three day-treatment programs. *The Journal of nervous and mental disease, 200*(9), 758-765.

Drozdek, B., Kamperman, A. M., Tol, W. A., Knipscheer, J. W., & Kleber, R. J. (2014). Seven-year follow-up study of symptoms in asylum seekers and refugees with PTSD treated with trauma-focused groups. *Journal of clinical psychology, 70*(4), 376-387.

Dumas, J. E., Arriaga, X. B., Begle, A. M., & Longoria, Z. N. (2011). Child and parental outcomes of a group parenting intervention for Latino families: A pilot study of the CANNE program. *Cultural diversity & ethnic minority psychology, 17*(1), 107-115.

Dybdahl, R. (2001). Children and mothers in war: an outcome study of a psychosocial intervention program. *Child development, 72*(4), 1214-1230. [10.1111/1467-8624.00343](https://doi.org/10.1111/1467-8624.00343)

Edelblute, H. B., Clark, S., Mann, L., McKenney, K. M., Bischof, J. J., & Kistler, C. (2014). Promotoras across the border: a pilot study addressing depression in Mexican women impacted by migration. *Journal of immigrant and minority health, 16*(3), 492-500.

Edwards, B., Smart, D., De Maio, J., Silbert, M., & Jenkinson, R. (2018). Cohort Profile: Building a New Life in Australia (BNLA): the longitudinal study of humanitarian migrants. *International journal of epidemiology, 47*(1), 20-20h.

Ekblad, S. (2020). To Increase Mental Health Literacy and Human Rights Among New-Coming, Low-Educated Mothers With Experience of War: A Culturally, Tailor-Made Group Health Promotion Intervention With Participatory Methodology Addressing Indirectly the Children. *Frontiers in Psychiatry, 11*, 611.

El-Khani, A., Cartwright, K., Ang, C., Henshaw, E., Tanveer, M., & Calam, R. (2018). Testing the feasibility of delivering and evaluating a child mental health recovery program enhanced with additional parenting sessions for families displaced by the Syrian conflict: A pilot study. Peace and Conflict: Journal of Peace Psychology, 24(2), 188–200. [https://doi.org/10.1037/pac0000287](https://psycnet.apa.org/doi/10.1037/pac0000287)

El-Khani, A., Maalouf, W., Baker, D. A., Zahra, N., Noubani, A., & Cartwright, K. (2020). Caregiving for children through conflict and displacement: a pilot study testing the feasibility of delivering and evaluating a light touch parenting intervention for caregivers in the West Bank. *International journal of psychology : Journal international de psychologie, 55*, 26-39.

El-Khani, A., Haar, K., Stojanovic, M., & Maalouf, W. (2021). Assessing the Feasibility of Providing a Family Skills Intervention, "Strong Families", for Refugee Families Residing in Reception Centers in Serbia. *International Journal of Environmental Research and Public Health, 18*(9). [doi:10.3390/ijerph18094530](https://doi.org/10.3390/ijerph18094530)Ellis, B. H., Miller, A. B., Abdi, S., Barrett, C., Blood, E. A., & Betancourt, T. S. (2013). Multi-tier mental health program for refugee youth. *Journal of consulting and clinical psychology, 81*(1), 129-140.

Ellis, B. H., Miller, A. B., Abdi, S., Barrett, C., Blood, E. A., & Betancourt, T. S. (2013). Multi-tier mental health program for refugee youth. *Journal of consulting and clinical psychology, 81*(1), 129-140.

Erickson, P. I. (1994). Lessons from a repeat pregnancy prevention program for Hispanic teenage mothers in east Los Angeles. *Family planning perspectives, 26*(4), 174-178.

Ertl, V., Pfeiffer, A., Schauer, E., Elbert, T., & Neuner, F. (2011). Community-implemented trauma therapy for former child soldiers in Northern Uganda: a randomized controlled trial. *JAMA, 306*(5), 503-512.

Esala, J. J., Vukovich, M. M., Hanbury, A., Kashyap, S., & Joscelyne, A. (2018). Collaborative care for refugees and torture survivors: Key findings from the literature. *Traumatology, 24*(3), 168-185.

Eylem, O., van Straten, A., de Wit, L., Rathod, S., Bhui, K., & Kerkhof, A. J. F. M. (2021). Reducing suicidal ideation among Turkish migrants in the Netherlands and in the UK: the feasibility of a randomised controlled trial of a guided online intervention. *Pilot and feasibility studies, 7*(1), 30.

Fabrizio, C. S., Stewart, S. M., Ip, A. K. Y., & Lam, T. H. (2014). Enhancing the Parent-Child Relationship: A Hong Kong Community-Based Randomized Controlled Trial. *Journal of Family Psychology, 28*(1), 42-53.

Falgas-Bague, I., Wang, Y., Banerjee, S., Ali, N., DiMarzio, K., Palao Vidal, D., & Alegria, M. (2019). Predictors of Adherence to Treatment in Behavioral Health Therapy for Latino Immigrants: The Importance of Trust. *Frontiers in psychiatry, 10*, 817.

Falkenstrom, F. (2010). Does psychotherapy for young adults in routine practice show similar results as therapy in randomized clinical trials? *Psychotherapy research : journal of the Society for Psychotherapy Research, 20*(2), 181-192.

Fazel, M., Doll, H., & Stein, A. (2009). A school-based mental health intervention for refugee children: an exploratory study. *Clinical Child Psychology and Psychiatry, 14*(2), 297-309.

Feddes, A. R., Mann, L., & Doosje, B. (2015). Increasing self-esteem and empathy to prevent violent radicalization: a longitudinal quantitative evaluation of a resilience training focused on adolescents with a dual identity. *Journal of Applied Social Psychology, 45*(7), 400-411.

Fischmann, T., Asseburg, L. K., Green, J., Hug, F., Neubert, V., Wan, M., & Leuzinger-Bohleber, M. (2020). Can Psychodynamically Oriented Early Prevention for "Children-at-Risk" in Urban Areas With High Social Problem Density Strengthen Their Developmental Potential? A Cluster Randomized Trial of Two Kindergarten-Based Prevention Programs. *Frontiers in psychology, 11*, 599477.

Flynn, A., Gonzalez, V., Mata, M., Salinas, L. A., & Atkins, A. (2020). Integrated care improves mental health in a medically underserved U.S.-Mexico border population. *Families, systems & health : the journal of collaborative family healthcare, 38*(2), 105-115.

Fuchs, C., Lee, J. K., Roemer, L., & Orsillo, S. M. (2013). Using Mindfulness- and Acceptance-Based Treatments With Clients From Nondominant Cultural and/or Marginalized Backgrounds: Clinical Considerations, Meta-Analysis Findings, and Introduction to the Special Series. *Cognitive and Behavioral Practice, 20*(1), 1-12.

Garcia-Huidobro, D., Diaspro-Higuera, M. O., Palma, D., Palma, R., Ortega, L., Shlafer, R., . . . Allen, M. L. (2019). Adaptive Recruitment and Parenting Interventions for Immigrant Latino Families with Adolescents. *Prevention science : the official journal of the Society for Prevention Research, 20*(1), 56-67.

Getanda, E. M., & Vostanis, P. (2020). Feasibility evaluation of psychosocial intervention for internally displaced youth in Kenya. *Journal of mental health (Abingdon, England)*, 1-9.

Goodkind, J. R., Bybee, D., Hess, J. M., Amer, S., Ndayisenga, M., Greene, R. N., . . . Pannah, M. (2020). Randomized Controlled Trial of a Multilevel Intervention to Address Social Determinants of Refugee Mental Health. *American journal of community psychology, 65*(3), 272-289.

Goodkind, J. R., Hess, J. M., Isakson, B., LaNoue, M., Githinji, A., Roche, N., . . . Parker, D. P. (2014). Reducing refugee mental health disparities: a community-based intervention to address postmigration stressors with African adults. *Psychological services, 11*(3), 333-346.

Goossens, F. X., Onrust, S. A., Monshouwer, K., & de Castro, B. O. (2016). Effectiveness of an empowerment program for adolescent second generation migrants: A cluster randomized controlled trial. *Children and Youth Services Review, 64*, 128-135.

Gordon, J. S., Staples, J. K., Blyta, A., Bytyqi, M., & Wilson, A. T. (2008). Treatment of posttraumatic stress disorder in postwar kosovar adolescents using mind-body skills groups: A randomized controlled trial. *Journal of Clinical Psychiatry, 69*(9), 1469-1476.

Greenfield, P. M., Espinoza, G., Monterroza-Brugger, M., Ruedas-Gracia, N., & Manago, A. M. (2020). Long-term parent–child separation through serial migration: Effects of a post-reunion intervention. *The School Community Journal, 30*(1), 267-298.

Grochtdreis, T., Rohr, S., Jung, F. U., Nagl, M., Renner, A., Kersting, A., . . . Dams, J. (2021). Health Care Services Utilization and Health-Related Quality of Life of Syrian Refugees with Post-Traumatic Stress Symptoms in Germany (the Sanadak Trial). *International journal of environmental research and public health, 18*(7).

Gurung, A., Subedi, P., Zhang, M., Li, C., Kelly, T., Kim, C., & Yun, K. (2020). Culturally-Appropriate Orientation Increases the Effectiveness of Mental Health First Aid Training for Bhutanese Refugees: Results from a Multi-state Program Evaluation. *Journal of immigrant and minority health, 22*(5), 957-964.

Haagen, J. F. G., Ter Heide, F. J. J., Mooren, T. M., Knipscheer, J. W., & Kleber, R. J. (2017). Predicting post-traumatic stress disorder treatment response in refugees: Multilevel analysis. *The British journal of clinical psychology, 56*(1), 69-83.

Haar, K., El-Khani, A., Molgaard, V., Maalouf, W., & Afghanistan field implementation, t. (2020). Strong families: a new family skills training programme for challenged and humanitarian settings: a single-arm intervention tested in Afghanistan. *BMC public health, 20*(1), 634.

Halvorsen, J. O., & Stenmark, H. (2010). Narrative exposure therapy for posttraumatic stress disorder in tortured refugees: a preliminary uncontrolled trial. *Scandinavian journal of psychology, 51*(6), 495-502.

Halvorsen, J. O., Stenmark, H., Neuner, F., & Nordahl, H. M. (2014). Does dissociation moderate treatment outcomes of narrative exposure therapy for PTSD? A secondary analysis from a randomized controlled clinical trial. *Behaviour Research and Therapy, 57*, 21-28.

Hasanovic, M., Srabovic, S., Rasidovic, M., Sehovic, M., Hasanbasic, E., Husanovic, J., & Hodzic, R. (2009). Psychosocial assistance to students with posttraumatic stress disorder in primary and secondary schools in post-war Bosnia Herzegovina. *Psychiatria Danubina, 21*(4), 463-473.

Heim, E., Ramia, J. A., Hana, R. A., Burchert, S., Carswell, K., Cornelisz, I., . . . Van't Hof, E. (2021). Step-by-step: Feasibility randomised controlled trial of a mobile-based intervention for depression among populations affected by adversity in Lebanon. *Internet interventions, 24*, 100380.

Hein, S., Bick, J., Issa, G., Aoude, L., Maalouf, C., Awar, A., . . . Ponguta, L. A. (2020). Maternal perceptions of father involvement among refugee and disadvantaged families in Beirut, Lebanon. *PloS one, 15*(3), e0229670.

Hendrickson, S. G. (2005). Reaching an underserved population with a randomly assigned home safety intervention. *Injury prevention : journal of the International Society for Child and Adolescent Injury Prevention, 11*(5), 313-317.

Hensel-Dittmann, D., Schauer, M., Ruf, M., Catani, C., Odenwald, M., Elbert, T., & Neuner, F. (2011). Treatment of traumatized victims of war and torture: a randomized controlled comparison of narrative exposure therapy and stress inoculation training. *Psychotherapy and psychosomatics, 80*(6), 345-352.

Hernandez, M. Y., & Organista, K. C. (2013). Entertainment-education? A fotonovela? A new strategy to improve depression literacy and help-seeking behaviors in at-risk immigrant Latinas. *American journal of community psychology, 52*(3), 224-235.

Hesselink, A. E., van Poppel, M. N., van Eijsden, M., Twisk, J. W. R., & van der Wal, M. F. (2012). The effectiveness of a perinatal education programme on smoking, infant care, and psychosocial health for ethnic Turkish women. *Midwifery, 28*(3), 306-313.

Hewage, K., Steel, Z., Mohsin, M., Tay, A. K., De Oliveira, J. C., Da Piedade, M., . . . Silove, D. (2018). A Wait-List Controlled Study of a Trauma-Focused Cognitive Behavioral Treatment for Intermittent Explosive Disorder in Timor-Leste. *American Journal of Orthopsychiatry, 88*(3), 282-294.

Hijazi, A. M., Lumley, M. A., Ziadni, M. S., Haddad, L., Rapport, L. J., & Arnetz, B. B. (2014). Brief narrative exposure therapy for posttraumatic stress in Iraqi refugees: a preliminary randomized clinical trial. *Journal of traumatic stress, 27*(3), 314-322.

Hinton, D. E., Chhean, D., Pich, V., Safren, S. A., Hofmann, S. G., & Pollack, M. H. (2005). A randomized controlled trial of cognitive-behavior therapy for Cambodian refugees with treatment-resistant PTSD and panic attacks: a cross-over design. *Journal of traumatic stress, 18*(6), 617-629.

Hinton, D. E., Hofmann, S. G., Pollack, M. H., & Otto, M. W. (2009). Mechanisms of efficacy of CBT for Cambodian refugees with PTSD: improvement in emotion regulation and orthostatic blood pressure response. *CNS neuroscience & therapeutics, 15*(3), 255-263.

Hinton, D. E., Hofmann, S. G., Rivera, E., Otto, M. W., & Pollack, M. H. (2011). Culturally adapted CBT (CA-CBT) for Latino women with treatment-resistant PTSD: A pilot study comparing CA-CBT to applied muscle relaxation. *Behaviour Research and Therapy, 49*(4), 275-280.

Holtrop, K., McNeil Smith, S., & Scott, J. C. (2015). Associations between positive parenting practices and child externalizing behavior in underserved Latino immigrant families. *Family process, 54*(2), 359-375.

Holzel, L. P., Ries, Z., Kriston, L., Dirmaier, J., Zill, J. M., Rummel-Kluge, C., . . . Harter, M. (2016). Effects of culture-sensitive adaptation of patient information material on usefulness in migrants: a multicentre, blinded randomised controlled trial. *BMJ open, 6*(11), e012008.

Hoskins, D., Duncan, L. G., Moskowitz, J. T., & Ordonez, A. E. (2018). Positive Adaptations for Trauma and Healing (PATH), a Pilot Study of Group Therapy With Latino Youth. *Psychological Trauma-Theory Research Practice and Policy, 10*(2), 163-172.

Hovey, J. D., Hurtado, G., & Seligman, L. D. (2014). Findings for a CBT Support Group for Latina Migrant Farmworkers in Western Colorado. *Current Psychology, 33*(3), 271-281.

Howes, C., Vu, J. A., & Hamilton, C. (2011). Mother-child attachment representation and relationships over time in Mexican-heritage families. *Journal of Research in Childhood Education, 25*(3), 228-247.

Hu, J., Wallace, D. C., McCoy, T. P., & Amirehsani, K. A. (2014). A family-based diabetes intervention for Hispanic adults and their family members. *The Diabetes educator, 40*(1), 48-59.

Huemer, J., Volkl-Kernstock, S., Yee, A., Bruckner, T., & Skala, K. (2016). "The Buoy": Utilization of a low-threshold ambulatory setting for traumatized children and adolescents in Austria. *Neuropsychiatrie : Klinik, Diagnostik, Therapie und Rehabilitation : Organ der Gesellschaft Osterreichischer Nervenarzte und Psychiater, 30*(1), 27-32.

Husby, S. R., Carlsson, J., Mathilde Scotte Jensen, A., Glahder Lindberg, L., & Sonne, C. (2020). Prevention of trauma-related mental health problems among refugees: A mixed-methods evaluation of the MindSpring group programme in Denmark. *Journal of community psychology, 48*(3), 1028-1039. doi: [10.1002/jcop.22323](https://doi.org/10.1002/jcop.22323)

Igreja, V., Kleijn, W. C., Schreuder, B. J. N., Van Dijk, J. A., & Verschuur, M. (2004). Testimony method to ameliorate post-traumatic stress symptoms - Community-based intervention study with Mozambican civil war survivors. *British Journal of Psychiatry, 184*, 251-257.

Im, H., Jettner, J. F., Warsame, A. H., Isse, M. M., Khoury, D., & Ross, A. I. (2018). Trauma-Informed Psychoeducation for Somali Refugee Youth in Urban Kenya: Effects on PTSD and Psychosocial Outcomes. *Journal of Child & Adolescent Trauma, 11*(4), 431-441.

Islam, N., Shapiro, E., Wyatt, L., Riley, L., Zanowiak, J., Ursua, R., & Trinh-Shevrin, C. (2017). Evaluating community health workers' attributes, roles, and pathways of action in immigrant communities. *Preventive medicine*, *103*, 1–7. https://doi.org/10.1016/j.ypmed.2017.07.020

Jalal, B., Kruger, Q., & Hinton, D. E. (2020). Culturally adapted CBT (CA-CBT) for traumatised indigenous South Africans (Sepedi): a randomised pilot trial comparing CA-CBT to applied muscle relaxation. *Intervention-International Journal of Mental Health Psychosocial Work and Counselling in Areas of Armed Conflict, 18*(1), 61-65. Retrieved from <Go to ISI>://WOS:000538043900008

Jang, Y., Chiriboga, D. A., Molinari, V., Roh, S., Park, Y., Kwon, S., & Cha, H. (2014). Telecounseling for the linguistically isolated: a pilot study with older Korean immigrants. *The Gerontologist, 54*(2), 290-296.

Javier, J. R., Reyes, A., Coffey, D. M., Schrager, S. M., Samson, A., Palinkas, L., . . . Miranda, J. (2019). Recruiting Filipino Immigrants in a Randomized Controlled Trial Promoting Enrollment in an Evidence-Based Parenting Intervention. *Journal of immigrant and minority health, 21*(2), 324-331.

Jin, Q., Mori, E., & Sakajo, A. (2020). Nursing intervention for preventing postpartum depressive symptoms among Chinese women in Japan. *Japan journal of nursing science : JJNS, 17*(4), e12336.

Jun, W. H., Hong, S. S., & Yang, S. (2014). Effects of a Psychological Adaptation Improvement Program for International Marriage Migrant Women in South Korea. *Asian Nursing Research, 8*(3), 232-238. Retrieved from <Go to ISI>://WOS:000343785800010

Kha, J., Rapee, R. M., & Bayer, J. K. (2022). Acceptability and Outcomes of the Cool Little Kids Parenting Group Program for Culturally and Linguistically Diverse Families Within an Australian Population-Based Study. *Child Psychiatry and Human Development*.

Khawaja, N. G., Kamo, R., & Ramirez, E. (2021). Building resilience in transcultural adults: investigating the effect of a strength-based programme. *Australian Psychologist, 56*(4), 324-334.

Khawaja, N. G., & Ramirez, E. (2019). Building Resilience in Transcultural Adolescents: an Evaluation of a Group Program. *Journal of Child and Family Studies, 28*(11), 2977-2987.

Kaltman, S., Hurtado de Mendoza, A., Serrano, A., & Gonzales, F. A. (2016). A mental health intervention strategy for low-income, trauma-exposed Latina immigrants in primary care: A preliminary study. *The American journal of orthopsychiatry, 86*(3), 345-354.

Kaltman, S., Serrano, A., Talisman, N., Magee, M. F., Cabassa, L. J., Pulgar-Vidal, O., & Peraza, D. (2016). Type 2 Diabetes and Depression: A Pilot Trial of an Integrated Self-management Intervention for Latino Immigrants. *The Diabetes educator, 42*(1), 87-95.

Kaltman, S., Watson, M. R., Campoli, M., Serrano, A., Talisman, N., Kirkpatrick, L., . . . Green, B. L. (2019). Treatment of depression and PTSD in primary care clinics serving uninsured low-income mostly Latina/o immigrants: A naturalistic prospective evaluation. *Cultural diversity & ethnic minority psychology, 25*(4), 579-589.

Kananian, S., Soltani, Y., Hinton, D., & Stangier, U. (2020). Culturally Adapted Cognitive Behavioral Therapy Plus Problem Management (CA-CBT+) With Afghan Refugees: A Randomized Controlled Pilot Study. *Journal of traumatic stress, 33*(6), 928-938.

Kangaslampi, S., & Peltonen, K. (2020). Changes in Traumatic Memories and Posttraumatic Cognitions Associate with PTSD Symptom Improvement in Treatment of Multiply Traumatized Children and Adolescents. *Journal of child & adolescent trauma, 13*(1), 103-112.

Kayrouz, R., Dear, B. F., Kayrouz, B., Karin, E., Gandy, M., & Titov, N. (2018). Meta-analysis of the efficacy and acceptability of cognitive-behavioural therapy for Arab adult populations experiencing anxiety, depression or post-traumatic stress disorder. *Cognitive Behaviour Therapy, 47*(5), 412-430.

Kelly, U. A., & Pich, K. (2014). Community-based PTSD treatment for ethnically diverse women who experienced intimate partner violence: a feasibility study. *Issues in mental health nursing, 35*(12), 906-913.

Kinzie, J. D., Kinzie, J. M., Sedighi, B., Woticha, A., Mohamed, H., & Riley, C. (2012). Prospective one-year treatment outcomes of tortured refugees: a psychiatric approach. *Torture : quarterly journal on rehabilitation of torture victims and prevention of torture, 22*(1), 1-10.

Kiropoulos, L. A., Griffiths, K. M., & Blashki, G. (2011). Effects of a multilingual information website intervention on the levels of depression literacy and depression-related stigma in Greek-born and Italian-born immigrants living in Australia: a randomized controlled trial. *Journal of medical Internet research, 13*(2), e34.

Kitchener, B. A., & Jorm, A. F. (2008). Mental Health First Aid: an international programme for early intervention. *Early intervention in psychiatry, 2*(1), 55-61.

Knefel, M., Kantor, V., Nicholson, A. A., Schiess-Jokanovic, J., Weindl, D., Schafer, I., & Lueger-Schuster, B. (2020). A brief transdiagnostic psychological intervention for Afghan asylum seekers and refugees in Austria: a randomized controlled trial. *Trials, 21*(1), 57.

Knox, L., Guerra, N. G., Williams, K. R., & Toro, R. (2011). Preventing children's aggression in immigrant Latino families: a mixed methods evaluation of the Families and Schools Together program. *American journal of community psychology, 48*(1), 65-76.

Kobel, S., Lammle, C., Wartha, O., Kesztyus, D., Wirt, T., & Steinacker, J. M. (2017). Effects of a Randomised Controlled School-Based Health Promotion Intervention on Obesity Related Behavioural Outcomes of Children with Migration Background. *Journal of immigrant and minority health, 19*(2), 254-262.

Kobel, S., Wirt, T., Schreiber, A., Kesztyus, D., Kettner, S., Erkelenz, N., . . . Steinacker, J. M. (2014). Intervention effects of a school-based health promotion programme on obesity related behavioural outcomes. *Journal of obesity, 2014*, 476230.

Koch, T., Ehring, T., & Liedl, A. (2020). Effectiveness of a transdiagnostic group intervention to enhance emotion regulation in young Afghan refugees: A pilot randomized controlled study. *Behaviour Research and Therapy, 132*, 103689.

Kocken, P. L., Zwanenburg, E. J.-v., & de Hoop, T. (2008). Effects of health education for migrant females with psychosomatic complaints treated by general practitioners. A randomised controlled evaluation study. *Patient education and counseling, 70*(1), 25-30.

Kodish, T., Weiss, B., Duong, J., Rodriguez, A., Anderson, G., Nguyen, H., . . . Lau, A. S. (2021). Interpersonal Psychotherapy-Adolescent Skills Training With Youth From Asian American and Immigrant Families: Cultural Considerations and Intervention Process. *Cognitive and Behavioral Practice, 28*(2), 147-166.

Kruse, J., Joksimovic, L., Cavka, M., Woller, W., & Schmitz, N. (2009). Effects of trauma-focused psychotherapy upon war refugees. *Journal of traumatic stress, 22*(6), 585-592.

Kwong, K., Chung, H., Cheal, K., Chou, J. C., & Chen, T. (2013). Depression care management for Chinese Americans in primary care: a feasibility pilot study. *Community mental health journal, 49*(2), 157-165.

Lachal, J., Moro, M. R., Carretier, E., Simon, A., Barry, C., Falissard, B., & Rouquette, A. (2020). Assessment of transcultural psychotherapy to treat resistant major depressive disorder in children and adolescents from migrant families: Protocol for a randomized controlled trial using mixed method and Bayesian approaches. *International journal of methods in psychiatric research, 29*(4), 1-10.

Lakkis, N. A., Osman, M. H., Aoude, L. C., Maalouf, C. J., Issa, H. G., & Issa, G. M. (2020). A Pilot Intervention to Promote Positive Parenting in Refugees from Syria in Lebanon and Jordan. *Frontiers in psychiatry, 11*, 257. doi.[10.3389/fpsyt.2020.00257](https://doi.org/10.3389/fpsyt.2020.00257)

Lancaster, S. L., & Gaede, C. (2020). A test of a resilience based intervention for mental health problems in Iraqi internally displaced person camps. *Anxiety, stress, and coping, 33*(6), 698-705.

Lau, A. S., Fung, J. J., Ho, L. Y., Liu, L. L., & Gudino, O. G. (2011). Parent training with high-risk immigrant chinese families: a pilot group randomized trial yielding practice-based evidence. *Behavior therapy, 42*(3), 413-426.

Lawrence, K. C., & Falaye, A. O. (2020). Trauma-focused counselling and social effectiveness skills training interventions on impaired psychological functioning of internally displaced adolescents in Nigeria. *Journal of Community & Applied Social Psychology, 30*(6), 616-627.

Le, H.-N., Perry, D. F., & Stuart, E. A. (2011). Randomized controlled trial of a preventive intervention for perinatal depression in high-risk Latinas. *Journal of consulting and clinical psychology, 79*(2), 135-141.

Le, H.-N., Perry, D. F., Villamil Grest, C., Genovez, M., Lieberman, K., Ortiz-Hernandez, S., & Serafini, C. (2020). A mixed methods evaluation of an intervention to prevent perinatal depression among Latina immigrants. *Journal of reproductive and infant psychology*, 1-13.

Lebiger-Vogel, J., Rickmeyer, C., Busse, A., Fritzemeyer, K., Ruger, B., & Leuzinger-Bohleber, M. (2015). FIRST STEPS - a randomized controlled trial on the evaluation of the implementation and effectiveness of two early prevention programs for promoting the social integration and a healthy development of children with an immigrant background from 0-3. *BMC psychology, 3*(1), 21.

Lecerof, S. S., Stafstrom, M., Emmelin, M., Westerling, R., & Ostergen, P. O. (2017). Findings from a prospective cohort study evaluating the effects of International Health Advisors' work on recently settled migrants' health. *BMC public health, 17*.

Lee, P.-I., Lai, H.-R., Lin, P.-C., Kuo, S.-Y., Lin, Y.-K., Chen, S.-R., & Lee, P.-H. (2020). Effects of a parenting sexual education program for immigrant parents: A cluster randomized trial. *Patient education and counseling, 103*(2), 343-349.

Lee, E. J. (2015). The effect of positive group psychotherapy on self-esteem and state anger among adolescents at Korean immigrant churches. *Archives of Psychiatric Nursing, 29*(2), 108-113.

Lee, M.-K. (2003). *Filial therapy with immigrant Korean parents in the United States.* (63). ProQuest Information & Learning,

Leidy, M. S., Guerra, N. G., & Toro, R. I. (2010). Positive Parenting, Family Cohesion, and Child Social Competence Among Immigrant Latino Families. *Journal of Family Psychology, 24*(3), 252-260.

Leijten, P., Raaijmakers, M. A. J., Orobio de Castro, B., & Matthys, W. (2016). Ethnic differences in problem perception: Immigrant mothers in a parenting intervention to reduce disruptive child behavior. *The American journal of orthopsychiatry, 86*(3), 323-331.

Leiler, A., Wasteson, E., Holmberg, J., & Bjarta, A. (2020). A Pilot Study of a Psychoeducational Group Intervention Delivered at Asylum Accommodation Centers-A Mixed Methods Approach. *International journal of environmental research and public health, 17*(23).

Lenglet, A., Lopes-Cardozo, B., Shanks, L., Blanton, C., Feo, C., Tsatsaeva, Z., . . . Pintaldi, G. (2018). Outcomes of an individual counselling programme in Grozny, Chechnya: a randomised controlled study. *BMJ open, 8*(8).

Leung, C., Tsang, S., & Dean, S. (2011). Outcome evaluation of the Hands-on Parent Empowerment (HOPE) program. *Research on Social Work Practice, 21*(5), 549-561.

Leung, C., Tsang, S., & Lo, C. (2017). Evaluation of Parent and Child Enhancement (PACE) program: Randomized controlled trial. *Research on Social Work Practice, 27*(1), 19-35.

Liedl, A., Muller, J., Morina, N., Karl, A., Denke, C., & Knaevelsrud, C. (2011). Physical activity within a CBT intervention improves coping with pain in traumatized refugees: results of a randomized controlled design. *Pain medicine (Malden, Mass.), 12*(2), 234-245.

Lindegaard, T., Seaton, F., Halaj, A., Berg, M., Kashoush, F., Barchini, R., . . . Andersson, G. (2021). Internet-based cognitive behavioural therapy for depression and anxiety among Arabic-speaking individuals in Sweden: *a pilot randomized controlled trial. Cogn Behav Ther, 50*(1), 47-66.

Litrownik, A.J., Elder, J.P., Campbell, N. R., Ayala, G. X., Slymen, D. J., Parra-Medina, D., . . .Lovato, C.Y. (2000). Evaluation of a tobacco and alcohol use prevention program for Hispanic migrant adolescents: promoting the protective factor of parent-child communication. Prev Med, 31(2 Pt 1), 124-133.

Löfvander, M., Engström, A., Theander, H., & Furhoff, A. K. (1997). Rehabilitation of young immigrants in primary care. A comparison between two treatment models. *Scand J Prim Health Care, 15*(3), 123-128. doi:10.3109/02813439709018501

Lopez-Maya, E., Olmstead, R., & Irwin, M. R. (2019). Mindfulness meditation and improvement in depressive symptoms among Spanish- and English speaking adults: A randomized, controlled, comparative efficacy trial. *PloS one, 14*(7), e0219425.

Lou, N. M., & Noels, K. A. (2020). Breaking the vicious cycle of language anxiety: Growth language mindsets improve lower-competence ESL students' intercultural interactions. *Contemporary Educational Psychology, 61*. Retrieved from <Go to ISI>://WOS:000552133600005

Luelmo, P., Kasari, C., & Fiesta Educativa, I. (2021). Randomized pilot study of a special education advocacy program for Latinx/minority parents of children with autism spectrum disorder. *Autism*. Retrieved from <Go to ISI>://WOS:000644627100001

Lutenbacher, M., Elkins, T., Dietrich, M. S., & Riggs, A. (2018). The Efficacy of Using Peer Mentors to Improve Maternal and Infant Health Outcomes in Hispanic Families: Findings from a Randomized Clinical Trial. *Maternal and child health journal, 22*, 92-104.

Magana, S., Hughes, M. T., Salkas, K., Gonzales, W., Nunez, G., Morales, M., . . . Moreno-Angarita, M. (2021) Implementing a Parent Education Intervention in Colombia: Assessing Parent Outcomes and Perceptions Across Delivery Modes. *Focus on Autism and Other Developmental Disabilities*. Retrieved from <Go to ISI>://WOS:000618515200001

Marksteiner, T., Janke, S., & Dickhauser, O. (2019). Effects of a brief psychological intervention on students' sense of belonging and educational outcomes: The role of students' migration and educational background. *Journal of school psychology, 75*, 41-57.

Marsiglia, F. F., Ayers, S. L., Baldwin-White, A., & Booth, J. (2016). Changing Latino Adolescents' Substance Use Norms and Behaviors: the Effects of Synchronized Youth and Parent Drug Use Prevention Interventions. *Prevention science : the official journal of the Society for Prevention Research, 17*(1), 1-12.

Marsiglia, F. F., Bermudez-Parsai, M., & Coonrod, D. (2010). Familias Sanas: an intervention designed to increase rates of postpartum visits among Latinas. *Journal of Health Care for the Poor and Underserved, 21*(3), 119-131.

Martinez, C. R., Jr., Eddy, J. M., McClure, H. H., & Cobb, C. L. (2022). Promoting Strong Latino Families Within an Emerging Immigration Context: Results of a Replication and Extension Trial of a Culturally Adapted Preventive Intervention. *Prevention science : the official journal of the Society for Prevention Research, 23*(2), 283-294.

Mateos-Fernandez, R., & Saavedra, J. (2020). Designing and assessing of an art-based intervention for undocumented migrants. *Arts & health*, 1-14.

McCabe, B. E., Mitchell, E. M., Gonzalez-Guarda, R. M., Peragallo, N., & Mitrani, V. B. (2017). Transnational Motherhood: Health of Hispanic Mothers in the United States Who Are Separated From Children. *Journal of transcultural nursing : official journal of the Transcultural Nursing Society, 28*(3), 243-250.

McDonald, L., Miller, H., & Sandler, J. (2015). A social ecological, relationship-based strategy for parent involvement: Families And Schools Together (FAST). *Journal of Childrens Services, 10*(3), 218-230. Retrieved from <Go to ISI>://WOS:000217782600004

McNaughton, D. B., Cowell, J. M., & Fogg, L. (2015). Efficacy of a Latino mother-child communication intervention in elementary schools. *The Journal of school nursing : the official publication of the National Association of School Nurses*, *31*(2), 126–134. https://doi.org/10.1177/1059840514526997

Meffert, S. M., Abdo, A. O., Alla, O. A. A., Elmakki, Y. O. M., Omer, A. A., Yousif, S., . . . Marmar, C. R. (2014). A pilot randomized controlled trial of interpersonal psychotherapy for Sudanese refugees in Cairo, Egypt. *Psychological Trauma: Theory, Research, Practice, and Policy, 6*(3), 240-249.

Mehrabi, T., Musavi, T., Ghazavi, Z., Zandieh, Z., & Zamani, A. (2011). The impact of group therapy training on social communications of Afghan immigrants. *Iranian journal of nursing and midwifery research, 16*(2), 148-152.

Meir, Y., Slone, M., & Levis, M. (2014). A randomized controlled study of a group intervention program to enhance mental health of children of illegal migrant workers. *Child & Youth Care Forum, 43*(2), 165-180.

Mendelsohn, A.L., Brockmeyer, C.A., Dreyer, B.P., Fierman, A.H., Berkule-Silberman, S.B. and Tomopoulos, S. (2010), Do verbal interactions with infants during electronic media exposure mitigate adverse impacts on their language development as toddlers?. Inf. Child Develop., 19: 577-593. <https://doi.org/10.1002/icd.711>

Metayer, N., Boulos, R., Tovar, A., Gervis, J., Abreu, J., Hval, E., . . . Economos, C. D. (2018). Recruitment of New Immigrants Into a Randomized Controlled Prevention Trial: The Live Well Experience. *The journal of primary prevention, 39*(5), 453-468.

Miller, K. E., Koppenol-Gonzalez, G., Jawad, A., Steen, F., Sassine, M., & Jordans, M. (2020). A Randomised Controlled Trial of the I-Deal Life Skills Intervention with Syrian Refugee Adolescents in Northern Lebanon. *Intervention-International Journal of Mental Health Psychosocial Work and Counselling in Areas of Armed Conflict, 18*(2), 119-128. Retrieved from <Go to ISI>://WOS:000596156000004

Miller, K. E., Koppenol-Gonzalez, G. V., Arnous, M., Tossyeh, F., Chen, A., Nahas, N., & Jordans, M. J. D. (2020). Supporting Syrian families displaced by armed conflict: A pilot randomized controlled trial of the Caregiver Support Intervention. *Child Abuse & Neglect, 106*, 104512. doi:[10.1016/j.chiabu.2020.104512](https://doi.org/10.1016/j.chiabu.2020.104512)

Morina, N., Ewers, S. M., Passardi, S., Schnyder, U., Knaevelsrud, C., Muller, J., . . . Schick, M. (2017). Mental health assessments in refugees and asylum seekers: evaluation of a tablet-assisted screening software. *Conflict and Health, 11*, 18.

Morris, J., Jones, L., Berrino, A., Jordans, M. J., Okema, L., & Crow, C. (2012). Does combining infant stimulation with emergency feeding improve psychosocial outcomes for displaced mothers and babies? A controlled evaluation from northern Uganda. *Am J Orthopsychiatry, 82*(3), 349-357. doi:10.1111/j.1939-0025.2012.01168.x

Morville, A.-L., Erlandsson, L.-K., Danneskiold-Samsoe, B., Amris, K., & Eklund, M. (2015). Satisfaction with daily occupations amongst asylum seekers in Denmark. *Scandinavian journal of occupational therapy, 22*(3), 207-215.

Mucic, D. (2010). Transcultural telepsychiatry and its impact on patient satisfaction. *Journal of Telemedicine and Telecare, 16*(5), 237-242.

Murray, L. K., Hall, B. J., Dorsey, S., Ugueto, A. M., Puffer, E. S., Sim, A., . . . Bolton, P. A. (2018). An evaluation of a common elements treatment approach for youth in Somali refugee camps. *Global mental health (Cambridge, England), 5*, e16.

Naeem, F., Phiri, P., Munshi, T., Rathod, S., Ayub, M., Gobbi, M., & Kingdon, D. (2015). Using cognitive behaviour therapy with South Asian Muslims: Findings from the culturally sensitive CBT project. *International Review of Psychiatry, 27*(3), 233-246.

Neuner, F., Kurreck, S., Ruf, M., Odenwald, M., Elbert, T., & Schauer, M. (2010). Can asylum-seekers with posttraumatic stress disorder be successfully treated? A randomized controlled pilot study. *Cognitive behaviour therapy, 39*(2), 81-91.

Neuner, F., Onyut, P. L., Ertl, V., Odenwald, M., Schauer, E., & Elbert, T. (2008). Treatment of posttraumatic stress disorder by trained lay counselors in an African refugee settlement: a randomized controlled trial. *Journal of consulting and clinical psychology, 76*(4), 686-694.

Neuner, F., Schauer, M., Klaschik, C., Karunakara, U., & Elbert, T. (2004). A comparison of narrative exposure therapy, supportive counseling, and psychoeducation for treating posttraumatic stress disorder in an african refugee settlement. *Journal of consulting and clinical psychology, 72*(4), 579-587.

Nickerson, A., Byrow, Y., Pajak, R., McMahon, T., Bryant, R. A., Christensen, H., & Liddell, B. J. (2020). 'Tell Your Story': a randomized controlled trial of an online intervention to reduce mental health stigma and increase help-seeking in refugee men with posttraumatic stress. *Psychological Medicine, 50*(5), 781-792.

Niederer, I., Kriemler, S., Zahner, L., Burgi, F., Ebenegger, V., Hartmann, T., . . . Puder, J. J. (2009). Influence of a lifestyle intervention in preschool children on physiological and psychological parameters (Ballabeina): study design of a cluster randomized controlled trial. *BMC public health, 9*, 94.

Nordbrandt, M. S., Sonne, C., Mortensen, E. L., & Carlsson, J. (2020). Trauma-affected refugees treated with basic body awareness therapy or mixed physical activity as augmentation to treatment as usual-A pragmatic randomised controlled trial. *PloS one, 15*(3), e0230300.

Northwood, A. K., Vukovich, M. M., Beckman, A., Walter, J. P., Josiah, N., Hudak, L., . . . Danner, C. C. (2020). Intensive psychotherapy and case management for Karen refugees with major depression in primary care: a pragmatic randomized control trial. *BMC family practice, 21*(1), 17.

Nygren, T., Brohede, D., Koshnaw, K., Osman, S. S., Johansson, R., & Andersson, G. (2019). Internet-based treatment of depressive symptoms in a Kurdish population: A randomized controlled trial. *Journal of clinical psychology, 75*(6), 985-998.

Opaas, M., & Hartmann, E. (2013). Rorschach assessment of traumatized refugees: an exploratory factor analysis. *Journal of personality assessment, 95*(5), 457-470.

Opaas, M., Wentzel-Larsen, T., & Varvin, S. (2020). The 10-year course of mental health, quality of life, and exile life functioning in traumatized refugees from treatment start. *PloS one, 15*(12), e0244730.

Osman, F., Flacking, R., Schön, U. K., & Klingberg-Allvin, M. (2017). A Support Program for Somali-born Parents on Children's Behavioral Problems. *Pediatrics, 139*(3). doi:10.1542/peds.2016-2764

Osman, F., Salari, R., Klingberg-Allvin, M., Schön, U.-K., & Flacking, R. (2017). Effects of a culturally tailored parenting support programme in Somali-born parents’ mental health and sense of competence in parenting: a randomised controlled trial. *BMJ open, 7*(12), e017600. doi:10.1136/bmjopen-2017-017600

Otto, M. W., Hinton, D., Korbly, N. B., Chea, A., Ba, P., Gershuny, B. S., & Pollack, M. H. (2003). Treatment of pharmacotherapy-refractory posttraumatic stress disorder among Cambodian refugees: a pilot study of combination treatment with cognitive-behavior therapy vs sertraline alone. *Behaviour research and therapy, 41*(11), 1271-1276.

Page-Reeves, J., Murray-Krezan, C., Regino, L., Perez, J., Bleecker, M., Perez, D., . . . Willging, C. E. (2021). A randomized control trial to test a peer support group approach for reducing social isolation and depression among female Mexican immigrants. *BMC public health, 21*(1), 119.

Panter-Brick, C., Dajani, R., Eggerman, M., Hermosilla, S., Sancilio, A., & Ager, A. (2018). Insecurity, distress and mental health: experimental and randomized controlled trials of a psychosocial intervention for youth affected by the Syrian crisis. *Journal of child psychology and psychiatry, and allied disciplines, 59*(5), 523-541.

Pantin, H., Coatsworth, J. D., Feaster, D. J., Newman, F. L., Briones, E., Prado, G., . . . Szapocznik, J. (2003). Familias Unidas: the efficacy of an intervention to promote parental investment in Hispanic immigrant families. *Prevention science : the official journal of the Society for Prevention Research, 4*(3), 189-201.

Parra-Cardona, J. R., Bybee, D., Sullivan, C. M., Rodriguez, M. M. D., Dates, B., Tams, L., & Bernal, G. (2017). Examining the impact of differential cultural adaptation with Latina/o immigrants exposed to adapted parent training interventions. *Journal of consulting and clinical psychology, 85*(1), 58-71.

Parra-Cardona, R., Fuentes-Balderrama, J., Vanderziel, A., Lopez-Zeron, G., Domenech Rodriguez, M. M., DeGarmo, D. S., & Anthony, J. C. (2022). A Culturally Adapted Parenting Intervention for Mexican-Origin Immigrant Families with Adolescents: Integrating Science, Culture, and a Focus on Immigration-Related Adversity. *Prevention science : the official journal of the Society for Prevention Research, 23*(2), 271-282.

Paunovic, N., & Ost, L. G. (2001). Cognitive-behavior therapy vs exposure therapy in the treatment of PTSD in refugees. *Behaviour research and therapy, 39*(10), 1183-1197.

Peltonen, K., & Kangaslampi, S. (2019). Treating children and adolescents with multiple traumas: a randomized clinical trial of narrative exposure therapy. *European Journal of Psychotraumatology, 10*(1), 1558708.

Pfeiffer, E., Sachser, C., Rohlmann, F., & Goldbeck, L. (2018). Effectiveness of a trauma-focused group intervention for young refugees: a randomized controlled trial. *Journal of child psychology and psychiatry, and allied disciplines, 59*(11), 1171-1179.

Piedra, L. M., & Byoun, S. J. (2012). Vida Alegre: Preliminary Findings of a Depression Intervention for Immigrant Latino Mothers. *Research on Social Work Practice, 22*(2), 138-150.

Pokhariyal, G. P., Rono, R., & Munywoki, S. (2013). Analysis of treatment methods for victims of torture in Kenya and East Africa Region. *Traumatology, 19*(2), 107-117.

Ponguta, L. A., Issa, G., Aoudeh, L., Maalouf, C., Hein, S. D., Zonderman, A. L., . . . Leckman, J. F. (2020). Effects of the Mother-Child Education Program on Parenting Stress and Disciplinary Practices Among Refugee and Other Marginalized Communities in Lebanon: A Pilot Randomized Controlled Trial. *Journal of the American Academy of Child and Adolescent Psychiatry, 59*(6), 727-738.

Poudel-Tandukar, K., Jacelon, C. S., Poudel, K. C., Bertone-Johnson, E. R., Rai, S., Ramdam, P., & Hollon, S. D. (2021). Mental health promotion among resettled Bhutanese adults in Massachusetts: Results of a peer-led family-centred Social and Emotional Well-being (SEW) intervention study. *Health & social care in the community*.

Priebe, S., Gavrilovic, J. J., Matanov, A., Franciskovic, T., Knezevic, G., Ljubotina, D., . . . Schutzwohl, M. (2010). Treatment Outcomes and Costs at Specialized Centers for the Treatment of PTSD After the War in Former Yugoslavia. *Psychiatric Services, 61*(6), 598-604.

Puffer, E. S., Annan, J., Sim, A. L., Salhi, C., & Betancourt, T. S. (2017). The impact of a family skills training intervention among Burmese migrant families in Thailand: A randomized controlled trial. *PloS one, 12*(3), e0172611. doi:10.1371/journal.pone.0172611

Qouta, S. R., Peltonen, K., Diab, S. Y., Anttila, S., Palosaari, E., & Punamaki, R. L. (2016). Psychosocial Intervention and Dreaming Among War-Affected Palestinian Children. *Dreaming, 26*(2), 95-118.

Ramos, G., Blizzard, A. M., Barroso, N. E., & Bagner, D. M. (2018). Parent Training and Skill Acquisition and Utilization Among Spanish- and English-Speaking Latino Families. *Journal of Child and Family Studies, 27*(1), 268-279.

Rees, B., Travis, F., Shapiro, D., & Chant, R. (2013). Reduction in posttraumatic stress symptoms in Congolese refugees practicing transcendental meditation. *Journal of traumatic stress, 26*(2), 295-298.

Rees, B., Travis, F., Shapiro, D., & Chant, R. (2014). Significant reductions in posttraumatic stress symptoms in Congolese refugees within 10 days of Transcendental Meditation practice. *Journal of traumatic stress, 27*(1), 112-115.

Reijneveld, S. A., Westhoff, M. H., & Hopman-Rock, M. (2003). Promotion of health and physical activity improves the mental health of elderly immigrants: results of a group randomised controlled trial among Turkish immigrants in the Netherlands aged 45 and over. *Journal of epidemiology and community health, 57*(6), 405-411.

Renner, W. (2009). The effectiveness of psychotherapy with refugees and asylum seekers: preliminary results from an Austrian study. *Journal of immigrant and minority health*, *11*(1), 41–45. https://doi.org/10.1007/s10903-007-9095-1

Renner, W., Banninger-Huber, E., & Peltzer, K. (2011). Culture-Sensitive and Resource Oriented Peer (CROP)-Groups as a community based intervention for trauma survivors: A randomized controlled pilot study with refugees and asylum seekers from Chechnya. *Australasian Journal of Disaster and Trauma Studies, 2011*(1), 1-13.

Renner, W., & Berry, J. W. (2011). Group Interventions were not Effective for Female Turkish Migrants with Recurrent Depression - Recommendations from a Randomized Controlled Study. Social behavior and personality, 39 9, 1217-1234.

Renner, W., Laireiter, A.-R., & Maier, M. J. (2012). Social Support from Sponsorships as a Moderator of Acculturative Stress: Predictors of Effects on Refugees and Asylum Seekers. *Social Behavior and Personality, 40*(1), 129-146.

Renzaho, A., & Vignjevic, S. (2011). The impact of a parenting intervention in Australia among migrants and refugees from Liberia, Sierra Leone, Congo, and Burundi: Results from the African Migrant Parenting Program. *Journal of Family Studies, 17*, 71-79. doi:10.5172/jfs.2011.17.1.71

Riesch, S. K., Brown, R. L., Anderson, L. S., Wang, K., Canty-Mitchell, J., & Johnson, D. L. (2012). Strengthening Families Program (10-14): Effects on the Family Environment. *Western Journal of Nursing Research, 34*(3), 340-376.

Robjant, K., Roberts, J., & Katona, C. (2017). Treating Posttraumatic Stress Disorder in Female Victims of Trafficking Using Narrative Exposure Therapy: A Retrospective Audit. *Frontiers in Psychiatry, 8*.

Robl, M., de Souza, M., Schiel, R., Gellhaus, I., Zwiauer, K., Holl, R. W., & Wiegand, S. (2013). The key role of psychosocial risk on therapeutic outcome in obese children and adolescents. Results from a longitudinal multicenter study. *Obesity facts, 6*(3), 297-305.

Rohr, S., Jung, F. U., Pabst, A., Grochtdreis, T., Dams, J., Nagl, M., . . . Riedel-Heller, S. G. (2021). A Self-Help App for Syrian Refugees With Posttraumatic Stress (Sanadak): Randomized Controlled Trial. *JMIR mHealth and uHealth, 9*(1), e24807.

Rondung, E., Leiler, A., Sarkadi, A., Bjarta, A., Lampa, E., Lofving, S. G., . . . Warner, G. (2022). Feasibility of a randomised trial of Teaching Recovery Techniques (TRT) with refugee youth: results from a pilot of the Swedish UnaccomPanied yOuth Refugee Trial (SUPpORT). *Pilot and Feasibility Studies, 8*(1), 40.

Rosser, R. L. (1986). Reality Therapy with the Khmer refugee resettled in the United States. Journal of Reality Therapy, 6(1), 21-29.

Rousseau, C., Beauregard, C., Daignault, K., Petrakos, H., Thombs, B. D., Steele, R., . . . Hechtman, L. (2014). A cluster randomized-controlled trial of a classroom-based drama workshop program to improve mental health outcomes among immigrant and refugee youth in special classes. PloS one, 9(8), e104704.

Rousseau, C., Benoit, M., Gauthier, M.-F., Lacroix, L., Alain, N., Rojas, M. V., . . . Bourassa, D. (2007). Classroom drama therapy program for immigrant and refugee adolescents: A pilot study. Clinical Child Psychology and Psychiatry, 12(3), 451-465.

Rousseau, C., Benoit, M., Lacroix, L., & Gauthier, M.-F. (2009). Evaluation of a sandplay program for preschoolers in a multiethnic neighborhood. *Journal of child psychology and psychiatry, and allied disciplines, 50*(6), 743-750.

Rousseau, C., Drapeau, A., Lacroix, L., Bagilishya, D., & Heusch, N. (2005). Evaluation of a classroom program of creative expression workshops for refugee and immigrant children. *Journal of child psychology and psychiatry, and allied disciplines, 46*(2), 180-185.

Salihu, D., Wong, E. M. L., & Kwan, R. Y. C. (2021). Effects of an African Circle Dance Programme on Internally Displaced Persons with Depressive Symptoms: A Quasi-Experimental Study. *International journal of environmental research and public health, 18*(2).

Sanchez-Aragon, A., Belzunegui-Eraso, A., & Prieto-Flores, O. (2020). Results of Mentoring in the Psychosocial Well-Being of Young Immigrants and Refugees in Spain. *Healthcare (Basel, Switzerland), 9*(1).

Sandahl, H., Carlsson, J., Sonne, C., Mortensen, E. L., Jennum, P., & Baandrup, L. (2021). Investigating the link between subjective sleep quality, symptoms of PTSD and level of functioning in a sample of trauma-affected refugees. *Sleep*.

Sandahl, H., Jennum, P., Baandrup, L., Lykke Mortensen, E., & Carlsson, J. (2021). Imagery rehearsal therapy and/or mianserin in treatment of refugees diagnosed with PTSD: Results from a randomized controlled trial. *Journal of sleep research*, e13276.

Sander, R., Laugesen, H., Skammeritz, S., Mortensen, E. L., & Carlsson, J. (2019). Interpreter-mediated psychotherapy with trauma-affected refugees - A retrospective cohort study. *Psychiatry research, 271*, 684-692.

Sarkadi, A., Adahl, K., Stenvall, E., Ssegonja, R., Batti, H., Gavra, P., . . . Salari, R. (2018). Teaching Recovery Techniques: evaluation of a group intervention for unaccompanied refugee minors with symptoms of PTSD in Sweden. *European Child & Adolescent Psychiatry, 27*(4), 467-479.

Schick, M., Morina, N., Mistridis, P., Schnyder, U., Bryant, R. A., & Nickerson, A. (2018). Changes in Post-migration Living Difficulties Predict Treatment Outcome in Traumatized Refugees. *Frontiers in Psychiatry, 9*, 476.

Schneider, A., Pfeiffer, A., Conrad, D., Elbert, T., Kolassa, I. T., & Wilker, S. (2020). Does cumulative exposure to traumatic stressors predict treatment outcome of community-implemented exposure-based therapy for PTSD? *European Journal of Psychotraumatology, 11*(1).

Schnur, E., Koffler, R., Wimpenny, N., Giller, H., & et al. (1995). Family child care and new immigrants: Cultural bridge and support. *Special Issue: Child day care, 74*(6), 1237-1248.

Schulz, W., Bothe, T., & Hahlweg, K. (2018). Prevention of psychological disorders and behavioral problems in children and adolescents with migration background and their mothers: Results of a 10-year follow-up. *Pravention psychischer Probleme und Verhaltensauffalligkeiten von Kindern und Jugendlichen mit Migrationshintergrund und deren Muttern: Ergebnisse eines 10-Jahres-Follow-up., 28*(2), 82-92.

Schytt, E., Wahlberg, A., Eltayb, A., Small, R., Tsekhmestruk, N., & Lindgren, H. (2020). Community-based doula support for migrant women during labour and birth: study protocol for a randomised controlled trial in Stockholm, Sweden (NCT03461640). *BMJ open, 10*(2), e031290.

Shattell, M. M., Quinlan-Colwell, A., Villalba, J., Ivers, N. N., & Mails, M. (2010). A cognitive-behavioral group therapy intervention with depressed Spanish-speaking Mexican women living in an emerging immigrant community in the United States. *ANS. Advances in nursing science, 33*(2), 158-169.

Shaw, S. A., Ward, K. P., Pillai, V., & Hinton, D. E. (2019). A group mental health randomized controlled trial for female refugees in Malaysia. *The American journal of orthopsychiatry, 89*(6), 665-674.

Shaw, S. A., Ward, K. P., Pillai, V., Ali, L. M., & Karim, H. (2021). A Randomized Clinical Trial Testing a Parenting Intervention Among Afghan and Rohingya Refugees in Malaysia. *Fam Process, 60*(3), 788-805. doi:10.1111/famp.12592

Sherman, D. K., Hartson, K. A., Binning, K. R., Purdie-Vaughns, V., Garcia, J., Taborsky-Barba, S., . . . Cohen, G. L. (2013). Deflecting the trajectory and changing the narrative: how self-affirmation affects academic performance and motivation under identity threat. *Journal of personality and social psychology, 104*(4), 591-618.

Siddiqui, F., Lindblad, U., Nilsson, P. M., & Bennet, L. (2019). Effects of a randomized, culturally adapted, lifestyle intervention on mental health among Middle-Eastern immigrants. *European journal of public health, 29*(5), 888-894.

Sim, A. L., Bowes, L., Maignant, S., Magber, S., & Gardner, F. (2020). Acceptability and Preliminary Outcomes of a Parenting Intervention for Syrian Refugees. *Research on Social Work Practice, 31*(1), 14-25. doi:10.1177/1049731520953627

Slewa-Younan, S., McKenzie, M., Thomson, R., Smith, M., Mohammad, Y., & Mond, J. (2020). Improving the mental wellbeing of Arabic speaking refugees: an evaluation of a mental health promotion program. *BMC psychiatry, 20*(1).

Small, E., Kim, Y. K., Praetorius, R. T., & Mitschke, D. B. (2016). Mental health treatment for resettled refugees: A comparison of three approaches. *Social Work in Mental Health, 14*(4), 342-359.

Smokowski, P. R., & Bacallao, M. (2009a). Entre Dos Mundos/Between Two Worlds youth violence prevention: Comparing psychodramatic and support group delivery formats. *Small Group Research, 40*(1), 3-27.

Smokowski, P. R., & Bacallao, M. (2009b). Entre dos mundos/between two worlds: Youth violence prevention for acculturating Latino families. *Special Issue: Intervention outcome research with Latinos: Social work's contributions., 19*(2), 165-178.

Somasundaram, D. (2010). Using cultural relaxation methods in post-trauma care among refugees in Australia. *International Journal of Culture and Mental Health, 3*(1), 16-24.

Sonderegger, R., Rombouts, S., Ocen, B., & McKeever, R. S. (2011). Trauma rehabilitation for war-affected persons in northern Uganda: a pilot evaluation of the EMPOWER programme. *The British journal of clinical psychology, 50*(3), 234-249.

Sonne, C., Mortensen, E. L., Silove, D., Palic, S., & Carlsson, J. (2021). Predictors of treatment outcomes for trauma-affected refugees - results from two randomised trials. *Journal of affective disorders, 282*, 194-202.

Staæhr, M. A. (2001). Psykoedukation med Kosovo Albanske flygtningebørn: En effektundersøgelse af et program til forebyggelse af alvorlige psykiske belastningsreaktioner = Psychoeducation with Kosovo Albanian refugee children. *Psyke & Logos, 22*(1), 127-146.

Stade, K., Skammeritz, S., Hjortkjaer, C., & Carlsson, J. (2015). "After all the traumas my body has been through, I feel good that it is still working."--Basic Body Awareness Therapy for traumatised refugees. *Torture : quarterly journal on rehabilitation of torture victims and prevention of torture, 25*(1), 33-50.

Stanford, M. S., Elverson, T. M., Padilla, J. I., & Rogers, E. B. (2014). Feasibility and efficacy of a peer-led recovery group program for war-related trauma in Libya. *South African Journal of Psychology, 44*(1), 97-105.

Steinert, C., Bumke, P. J., Hollekamp, R. L., Larisch, A., Leichsenring, F., Matthess, H., . . . Kruse, J. (2017). Resource activation for treating post-traumatic stress disorder, co-morbid symptoms and impaired functioning: a randomized controlled trial in Cambodia. *Psychological Medicine, 47*(3), 553-564.

Stenmark, H., Catani, C., Neuner, F., Elbert, T., & Holen, A. (2013). Treating PTSD in refugees and asylum seekers within the general health care system. A randomized controlled multicenter study. *Behaviour research and therapy, 51*(10), 641-647.

Sternberg, R. M., Napoles, A. M., Gregorich, S., & Stewart, A. L. (2019). Mentes Positivas en Accion: Feasibility Study of a Promotor-Delivered Cognitive Behavioral Stress Management Program for Low-Income Spanish-Speaking Latinas. *Health Equity, 3*(1), 155-161.

Sternberg, R. M., Stewart, A. L., & Napoles, A. M. (2021). Mentes Positivas en Accion: A Randomized Feasibility Study of a Promotor-Delivered Cognitive Behavioral Stress Management Program for Low-Income Spanish-Speaking Latinos. *Health Equity, 5*(1), 218-226.

Tay, A. K., Mung, H. K., Miah, M. A. A., Balasundaram, S., Ventevogel, P., Badrudduza, M., . . . Silove, D. (2020). An Integrative Adapt Therapy for common mental health symptoms and adaptive stress amongst Rohingya, Chin, and Kachin refugees living in Malaysia: A randomized controlled trial. *PLoS medicine, 17*(3), e1003073.

Ter Heide, F. J. J., Mooren, T. M., Kleijn, W., de Jongh, A., & Kleber, R. J. (2011). EMDR versus stabilisation in traumatised asylum seekers and refugees: results of a pilot study. *European Journal of Psychotraumatology, 2*.

Ter Heide, F. J. J., Mooren, T. M., van de Schoot, R., de Jongh, A., & Kleber, R. J. (2016). Eye movement desensitisation and reprocessing therapy v. stabilisation as usual for refugees: randomised controlled trial. *The British journal of psychiatry : the journal of mental science, 209*(4), 311-318.

Tol, W. A., Leku, M. R., Lakin, D. P., Carswell, K., Augustinavicius, J., Adaku, A., . . . van Ommeren, M. (2020). Guided self-help to reduce psychological distress in South Sudanese female refugees in Uganda: a cluster randomised trial. *The Lancet. Global health, 8*(2), e254-e263.

Tucker, C., Schieffer, K., Lenz, S., & Smith, S. (2021). Sunshine Circles: Randomized controlled trial of an attachment-based play group with preschool students who are at-risk. *Journal of Child and Adolescent Counseling, 7*(3), 161-175.

Unger, J. B., Cabassa, L. J., Molina, G. B., Contreras, S., & Baron, M. (2013). Evaluation of a fotonovela to increase depression knowledge and reduce stigma among Hispanic adults. *Journal of immigrant and minority health, 15*(2), 398-406.

Unlu Ince, B., Cuijpers, P., van 't Hof, E., van Ballegooijen, W., Christensen, H., & Riper, H. (2013). Internet-based, culturally sensitive, problem-solving therapy for Turkish migrants with depression: randomized controlled trial. *Journal of medical Internet research, 15*(10), e227.

van Heemstra, H. E., Scholte, W. F., Haagen, J. F. G., & Boelen, P. A. (2019). 7ROSES, a transdiagnostic intervention for promoting self-efficacy in traumatized refugees: a first quantitative evaluation. *European Journal of Psychotraumatology, 10*(1), 1673062.

van Loon, A., van Schaik, D. J. F., Dekker, J. J., & Beekman, A. T. F. (2011). Effectiveness of an intercultural module added to the treatment guidelines for Moroccan and Turkish patients with depressive and anxiety disorders. *BMC psychiatry, 11*, 13.

van Wyk, S., Schweitzer, R., Brough, M., Vromans, L., & Murray, K. (2012). A longitudinal study of mental health in refugees from Burma: the impact of therapeutic interventions. *The Australian and New Zealand journal of psychiatry, 46*(10), 995-1003.

Vijayakumar, L., Mohanraj, R., Kumar, S., Jeyaseelan, V., Sriram, S., & Shanmugam, M. (2017). CASP - An intervention by community volunteers to reduce suicidal behaviour among refugees. *The International journal of social psychiatry, 63*(7), 589-597.

Wade-Bohleber, L., Hofer, A., Ottiger, M., von Wyl, A., Stulz, A., & Rumpel, S. (2022). [Can the GroupTherapy "Arriving" Support Refugee Mothers with their Young Children? Results from a Longitudinal Pilot Study]. *Aacho" - ein niederschwelliges gruppentherapeutisches Angebot fur gefluchtete Mutter mit Kleinkindern: Ergebnisse einer evaluativen Pilotstudie., 71*(2), 119-140.

Wagner, J., Kong, S., Kuoch, T., Scully, M. F., Tan, H. K., & Bermudez-Millan, A. (2015). Patient reported outcomes of Eat, Walk, Sleep: A cardiometabolic lifestyle program for Cambodian Americans delivered by community health workers. *Journal of Health Care for the Poor and Underserved, 26*(2), 441-452.

Wang, S. J., Bytyci, A., Izeti, S., Kallaba, M., Rushiti, F., Montgomery, E., & Modvig, J. (2017). A novel bio-psycho-social approach for rehabilitation of traumatized victims of torture and war in the post-conflict context: a pilot randomized controlled trial in Kosovo. *Conflict and Health, 10*.

Weine, S., Kulauzovic, Y., Klebic, A., Besic, S., Mujagic, A., Muzurovic, J., . . . Rolland, J. (2008). Evaluating a multiple-family group access intervention for refugees with PTSD. *Journal of marital and family therapy, 34*(2), 149-164.

Weine, S. M., Kulenovic, A. D., Pavkovic, I., & Gibbons, R. (1998). Testimony psychotherapy in Bosnian refugees: a pilot study. *The American journal of psychiatry, 155*(12), 1720-1726.

Weinstein, N., Khabbaz, F., & Legate, N. (2016). Enhancing need satisfaction to reduce psychological distress in Syrian refugees. *Journal of consulting and clinical psychology, 84*(7), 645-650.

Weisleder, A., Cates, C. B., Dreyer, B. P., Berkule Johnson, S., Huberman, H. S., Seery, A. M., . . . Mendelsohn, A. L. (2016). Promotion of Positive Parenting and Prevention of Socioemotional Disparities. *Pediatrics, 137*(2), e20153239.

Weiss, W. M., Murray, L. K., Zangana, G. A. S., Mahmooth, Z., Kaysen, D., Dorsey, S., . . . Bolton, P. (2015). Community-based mental health treatments for survivors of torture and militant attacks in Southern Iraq: a randomized control trial. *BMC psychiatry, 15*.

Williamson, A. A., Knox, L., Guerra, N. G., & Williams, K. R. (2014). A pilot randomized trial of community-based parent training for immigrant Latina mothers. *American journal of community psychology, 53*(1), 47-59.

Yagmur, S., Mesman, J., Malda, M., Bakermans-Kranenburg, M. J., & Ekmekci, H. (2014). Video-feedback intervention increases sensitive parenting in ethnic minority mothers: a randomized control trial. *Attachment & human development, 16*(4), 371-386.

Yankey, T., & Biswas, U. N. (2019). Impact of life skills training on psychosocial well-being of Tibetan refugee adolescents. *International Journal of Migration Health and Social Care, 15*(4), 272-284.

Yeung, A., Martinson, M. A., Baer, L., Chen, J., Clain, A., Williams, A., . . . Fava, M. (2016). The Effectiveness of Telepsychiatry-Based Culturally Sensitive Collaborative Treatment for Depressed Chinese American Immigrants: A Randomized Controlled Trial. *The Journal of clinical psychiatry, 77*(8), e996-e1002.

Yeung, A., Shyu, I., Fisher, L., Wu, S., Yang, H., & Fava, M. (2010). Culturally sensitive collaborative treatment for depressed chinese americans in primary care. *American journal of public health*, *100*(12), 2397–2402.

Young, M., Salerno, J., Rockhill, S., Hernandez, A., & DeMaria, R. (2021). Evaluation of the Impact of a Healthy Relationship Program Among US Refugees. *Family relations, 70*(5), 1643-1656.

Yu, N. X., Lam, T. H., Liu, I. K. F., & Stewart, S. M. (2015). Mediation of Short and Longer Term Effects of an Intervention Program to Enhance Resilience in Immigrants from Mainland China to Hong Kong. *Frontiers in psychology, 6*.

Yu, X. N., Stewart, S. M., Chui, J. P. L., Ho, J. L. Y., Li, A. C. H., & Lam, T. H. (2014). A Pilot Randomized Controlled Trial to Decrease Adaptation Difficulties in Chinese New Immigrants to Hong Kong. *Behavior Therapy, 45*(1), 137-152.

Yurtsever, A., Konuk, E., Akyuz, T., Zat, Z., Tukel, F., Cetinkaya, M., . . . Shapiro, E. (2018). An Eye Movement Desensitization and Reprocessing (EMDR) Group Intervention for Syrian Refugees With Post-traumatic Stress Symptoms: Results of a Randomized Controlled Trial. *Frontiers in psychology, 9*, 493.

Zehetmair, C., Kaufmann, C., Tegeler, I., Kindermann, D., Junne, F., Zipfel, S., . . . Nikendei, C. (2018). Psychotherapeutic Group Intervention for Traumatized Male Refugees Using Imaginative Stabilization Techniques-A Pilot Study in a German Reception Center. *Frontiers in Psychiatry, 9*.

**Wrong intervention**

Folkes, C. E. (2002). Thought field therapy and trauma recovery. *International journal of emergency mental health, 4*(2), 99-103.

Hamid, S., Dashash, M., & Latifeh, Y. (2021). A short-term approach for promoting oral health of internally displaced children with PTSD: the key is improving mental health-results from a quasi-randomized trial. *BMC oral health, 21*(1), 58.

Lempertz, D., Wichmann, M., Enderle, E., Stellermann-Strehlow, K., Pawils, S., & Metzner, F. (2020). Pre-post study to assess EMDR-based group therapy for traumatized refugee preschoolers. *Journal of EMDR Practice and Research, 14*(1), 31-45.

Metzler, J., Diaconu, K., Hermosilla, S., Kaijuka, R., Ebulu, G., Savage, K., & Ager, A. (2019). Short- and longer-term impacts of Child Friendly Space Interventions in Rwamwanja Refugee Settlement, Uganda. *Journal of child psychology and psychiatry, and allied disciplines, 60*(11), 1152-1163.

Metzler, J., Jonfa, M., Savage, K., & Ager, A. (2021). Educational, psychosocial, and protection outcomes of child- and youth-focused programming with Somali refugees in Dollo Ado, Ethiopia. *Disasters, 45*(1), 67-85.

Momartin, S., Coello, M., Pittaway, E., Downham, R., & Aroche, J. (2019). Capoeira Angola: An alternative intervention program for traumatized adolescent refugees from war-torn countries. *Torture : quarterly journal on rehabilitation of torture victims and prevention of torture, 29*(1), 85-96.

Oras, R., de Ezpeleta, S. C., & Ahmad, A. (2004). Treatment of traumatized refugee children with Eye Movement Desensitization and Reprocessing in a psychodynamic context. *Nordic journal of psychiatry, 58*(3), 199-203.

O'Leary, P., Hutchinson, A., & Squire, J. (2015). Community-based child protection with Palestinian refugees in South Lebanon: Engendering hope and safety. *International Social Work, 58*(5), 717-731.

Ruf, M., Schauer, M., Neuner, F., Catani, C., Schauer, E., & Elbert, T. (2010). Narrative exposure therapy for 7- to 16-year-olds: a randomized controlled trial with traumatized refugee children. *Journal of traumatic stress, 23*(4), 437-445.

Schottelkorb, A. A., Doumas, D. M., & Garcia, R. (2012). Treatment for childhood refugee trauma: A randomized, controlled trial. *International Journal of Play Therapy, 21*(2), 57-73.

Unterhitzenberger, J., Wintersohl, S., Lang, M., Konig, J., & Rosner, R. (2019). Providing manualized individual trauma-focused CBT to unaccompanied refugee minors with uncertain residence status: a pilot study. *Child and Adolescent Psychiatry and Mental Health, 13*.

Walg, M., Angern, J. S., Michalak, J., & Hapfelmeier, G. (2020). [Effectiveness of stabilization training for adolescent refugees with trauma-induced disorders: A randomized controlled trial]. *Wirksamkeit des Stabilisierungstrainings fur jugendliche Fluchtlinge mit Traumafolgestorungen: Eine randomisierte Kontrollgruppenstudie., 48*(5), 369-379.

Zapata, G. P., & Hargreaves, D. J. (2018). The effects of musical activities on the self-esteem of displaced children in Colombia. *Psychology of Music, 46*(4), 540-550.

**Wrong study design**

Anders, M., & Christiansen, H. (2016). Unaccompanied Refugee Minors: A Systematic Review of Psychological Interventions. *Kindheit Und Entwicklung, 25*(4), 216-230.

Barenbaum, J., Ruchkin, V., & Schwab-Stone, M. (2004). The psychosocial aspects of children exposed to war: practice and policy initiatives. *Journal of Child Psychology and Psychiatry, 45*(1), 41-62.

Brown, R. C., Witt, A., Fegert, J. M., Keller, F., Rassenhofer, M., & Plener, P. L. (2017). Psychosocial interventions for children and adolescents after man-made and natural disasters: a meta-analysis and systematic review. *Psychological Medicine, 47*(11), 1893-1905.

**Wrong outcome**

Arabacioglu, B., & Bagceli Kahraman, P. (2020). The Effect of Social Skills Education on 60-69 Months Old Syrian Children's Social Skills, Problem Solving and Adaptation. *Cukurova University Faculty of Education Journal, 49*(2), 734-768.

Erdemir, E. (2022). Summer Preschools for Syrian Refugee and Host Community Children in Turkey: A Model of Contextually Sensitive Early Intervention. *Early Education and Development*, 27.

**Too few participants**

Park, J. K., Park, J., Elbert, T., & Kim, S. J. (2020). Effects of Narrative Exposure Therapy on Posttraumatic Stress Disorder, Depression, and Insomnia in Traumatized North Korean Refugee Youth. *Journal of traumatic stress, 33*(3), 353-359.

Su, S.-H., & Tsai, M.-H. (2016). Group play therapy with children of new immigrants in Taiwan who are exhibiting relationship difficulties. *Special Issue: International Journal of Play Therapy's 25th Anniversary: Play Therapy in Schools, 25*(2), 91-101.

**Secondary analysis**

Dajani, R., Hadfield, K., van Uum, S., Greff, M., & Panter-Brick, C. (2018). Hair cortisol concentrations in war-affected adolescents: A prospective intervention trial. Psychoneuroendocrinology, 89, 138-146.

Kazandjian, C., Militello, L. K., & Doumit, R. (2020). Sex Differences on Quality of Life and Mental Health Outcomes When Using a Brief Cognitive-Behavioral Skill Building Intervention with Adolescent Syrian Refugees: A Secondary Analysis. *Community Mental Health Journal, 56*(1), 157-164.

Pfeiffer, E., Sachser, C., Tutus, D., Fegert, J. M., & Plener, P. L. (2019). Trauma-focused group intervention for unaccompanied young refugees: "Mein Weg"-predictors of treatment outcomes and sustainability of treatment effects. *Child and Adolescent Psychiatry and Mental Health, 13*, 18.

**No access to data**

Gupta, L., & Zimmer, C. (2008). Psychosocial intervention for war-affected children in Sierra Leone. *The British journal of psychiatry : the journal of mental science, 192*(3), 212-216.

**No validated measures**

Kneer, J., van Eldik, A. K., Jansz, J., Eischeid, S., & Usta, M. (2019). With a Little Help from My Friends: Peer Coaching for Refugee Adolescents and the Role of Social Media. *Media and Communication, 7*(2), 264-274.

**Wrong data type**

Scheiber, B., Greinz, G., Hillebrand, J. B., Wilhelm, F. H., & Blechert, J. (2019). Resilience training for unaccompanied refugee minors: A randomized controlled pilot study. *Resilienztraining fur unbegleitete minderjahrige Fluchtlinge: Eine randomisiert-kontrollierte Pilotstudie., 28*(3), 173-181.

**Wrong publication type**

Ceballos, P. (2009). *School-based child parent relationship therapy (CPRT) with low income first generation immigrant Hispanic parents: Effects on child behavior and parent-child relationship stress.* (69). ProQuest Information & Learning,

Dunne, J. E. (1993). Cambodian follow-up. *Journal of the American Academy of Child and Adolescent Psychiatry, 32*(6), 1305-1306.

Durbeej, N., McDiarmid, S., Sarkadi, A., Feldman, I., Punamaki, R.-L., Kankaanpaa, R., . . . Osman, F. (2021). Evaluation of a school-based intervention to promote mental health of refugee youth in Sweden (The RefugeesWellSchool Trial): study protocol for a cluster randomized controlled trial. *Trials, 22*(1), 98.

Ekblad, S. (1994). [Importance of follow-up of refugee children. Risk factors are changing during the different phases of the crisis]. *Viktigt folja upp asylsokande barn. Riskfaktorerna andras under krisfaserna., 91*(44), 4012-4017.

Falb, K. L., Tanner, S., Ward, L., Erksine, D., Noble, E., Assazenew, A., . . . Stark, L. (2016). Creating opportunities through mentorship, parental involvement, and safe spaces (COMPASS) program: multi-country study protocol to protect girls from violence in humanitarian settings. *BMC public health, 16*, 231.

Leckman, J. F. (2020).Effects of the Mother-Child Education Program on Parenting Stress and Disciplinary Practices among refugee and other marginalized communities in Lebanon: A Pilot Randomized Controlled Trial. *Journal of the American Academy of Child and Adolescent Psychiatry, 59*(10), S118-S119.

Nathan, S., Bunde-Birouste, A., Evers, C., Kemp, L., MacKenzie, J., & Henley, R. (2010). Social cohesion through football: a quasi-experimental mixed methods design to evaluate a complex health promotion program. *BMC public health, 10*, 587.

Onyut, L. P., Neuner, F., Schauer, E., Ertl, V., Odenwald, M., Schauer, M., & Elbert, T. (2004). The Nakivale Camp Mental Health Project: Building local competency for psychological assistance to traumatised refugees. *Intervention: International Journal of Mental Health, Psychosocial Work & Counselling in Areas of Armed Conflict, 2*(2), 90-107.

Puccinelli, M. (2018). *Treatment and moderator effects in a randomized controlled trial of culturally informed and flexible family treatment for adolescents (CIFFTA): An investigation of the relationships between stress, acculturation, and parenting practices.* (79). ProQuest Information & Learning.

**Not retrieved**

de Kom, A. A., & Bleeker, J. A. (1991). Acute reactive psychosis among immigrants in Amsterdam. *Lancet (London, England), 337*(8734), 185-186.

Fox, P. G., Cowell, J. M., Montgomery, A. C., & Willgerodt, M. A. (1998). Southeast Asian refugee women and depression: a nursing intervention. *The international journal of psychiatric nursing research, 4*(1), 423-432.

Silove, D., Chang, R., & Manicavasagar, V. (1995). Impact of recounting trauma stories on the emotional state of Cambodian refugees. *Psychiatric services (Washington, D.C.), 46*(12), 1287-1288.

**Studies included from reference lists of other meta-literature but later excluded**

One study was included from the reference list of an existing review but later excluded as it did not include an appropriate outcome.

Baker, F., & Jones, C. (2006). The effect of music therapy services on classroom behaviours of newly arrived refugee students in Australia-a pilot study. *Emotional and Behavioural Difficulties*, *11*(4), 249–260. https://doi.org/10.1080/13632750601022170
