# Supplementary material for: The efficacy of psychological prevention, and health promotion interventions targeting psychological health, wellbeing or resilience among forced migrant children and youth: a systematic review and meta-analysis
Source: Eur Child Adolesc Psychiatry. 2024 Apr 16;34(1):123–40. doi: 10.1007/s00787-024-02424-8 (PMC11805832; doi:10.1007/s00787-024-02424-8)
Supplement: Supplementary file 7 — Supplementary file7 (DOCX 28 KB) [file 787_2024_2424_MOESM7_ESM.docx]

Supplementary Information 7

**The efficacy of psychological prevention, and health promotion interventions targeting psychological health, wellbeing or resilience among forced migrant children and youth: a systematic review and meta-analysis**

**European Child and Adolescent Psychiatry**

Clover Jack Giles ^1^, Maja Västhagen ^2^, Livia Van Leuven ^2^,

Anna Edenius^3^, Ata Ghaderi ^2^, Pia Enebrink ^2^

^1^ School of Behavioural, Social and Legal Sciences, Örebro University, Örebro, Sweden

^2^ Department of Clinical Neuroscience, Karolinska Institutet, Stockholm, Sweden

^3^ Department of Medicine, Karolinska Institutet, Stockholm, Sweden

*Corresponding author:*

Clover Jack Giles (CJG)

[clover.giles@oru.se](mailto:clover.giles@oru.se)

# Supplementary Information 7: Study Characteristics, Outcomes, Measures, and Overall Risk of Bias

| Author and year | Journal | Title | Study type | N included  (Total = 5741) | Outcomes within scope of meta-analysis | Outcomes within scope of review | Measures (validation) | RoB |
| --- | --- | --- | --- | --- | --- | --- | --- | --- |
| Bolton et al., 2007 | JAMA | Interventions for Depression Symptoms Among Adolescent Survivors of War and Displacement in Northern Uganda: A Randomized Controlled Trial | RCT  (3 arm) | 314  (IPT-G= 105, Creative Play = 105, Control = 104) | Depression | NA | APAI Acholi Psychosocial Assessment Instrument (developed and validated for this study) | Mod |
| Cardeli et al., 2020 | J. of School Health | Bhutanese Refugee Youth: The Importance of Assessing and Addressing Psychosocial Needs in a School Setting | Pre-post | 35 | Depression | PTSD | DSRC-C Depression Self rating scale for children (previously validated for refugee and East Asian children), UCLA-PTSD-Reaction Index (good convergent validity for refugee youth) | Ser |
| Doumit et al., 2020 | Clin. Nurs. Res. | COPE for Adolescent Syrian Refugees in Lebanon: A Brief Cognitive-Behavioural Skill-Building Intervention to Improve Quality of Life and Promote Positive Mental Health. | Pre-post | 40 | Depression | Anxiety, Quality of Life | PHQ-9 Patient Health Questionnaire (validated in Arabic, non-refugee adults), GAD-7 Generalized Anxiety Disorder-7 (validated in Arabic, non-refugee children and youth), PedQol-4 Paediatric Quality of Life Inventory (validated in Arabic, non-refugee children and youth) | Ser |
| Ehntholt et al., 2005 | Clin. Child Psychol. And Psychiatry | School-based Cognitive-Behavioural Therapy Group Intervention for Refugee Children who have Experienced War-related Trauma | RCT  (quasi) | 26  (CBT = 15  Control = 11) | Depression | Anxiety, Teacher rated Emotional and behavioural problems, PTSD | DSRS Birleson Depression Self-Rating Scale (not specifically validated for population), R-IES Revised impact of events scale (developed for war effected children), Teacher version SDQ Strengths and difficulties questionnaire (English version not specifically validated for refugee populations), RCMAS Revised children’s Manifest Anxiety Scale (not specifically validated for population) | Mod |
| Foka et al., 2021 | Dev. And Psychopathol. | Promoting well-being in refugee children: An exploratory controlled trial of a positive psychology intervention delivered in Greek refugee camps | RCT (quasi) | 72  (Intervention = 32  Control = 36) | Depression | Wellbeing | CES-DC Center for Epidemiological Studies Depression Scale for Children (back-translated and validated for use with refugee children), WHO-5 World Health Organisation Well-being index (back-translated, not specifically validated for refugees) | Mod |
| Fox et al., 2005 | The Int. J. of Psychiatr. Nurs. | Southeast Asian Refugee Children: A School-Based Mental Health Intervention | Pre-post | 58 | Depression | NA | CDI Children’s Depression Inventory (translated by bilingual teachers and items assessed for cultural appropriateness) | Ser |
| Garoff et al., 2018 | Scand. J. of Psychol. | Development and implementation of a group based mental health intervention for unaccompanied minors | Pre-post | 18 | NA | PTSD, Caretaker rated Emotional and behavioural problems, | CRIES – The Child Impact of Event Scale 13 (developed for war effected children), Parent version SDQ Strengths and Difficulties Questionnaire (Finnish version not specifically validated for refugee populations), | Ser |
| Gormez et al., 2017 | Psychiatry and Clin. Psychopharmacol. | Evaluation of a school-based, teacher-delivered psychological intervention group program for trauma-affected Syrian refugee children in Istanbul, Turkey | Pre-post | 32 | NA | Anxiety, Child rated Emotional and behavioural problems, PTSD | CPTS-RI The child post-traumatic stress – reaction index (previously validated among war affected Palestinian children), SDQ Strengths and Difficulties Questionnaire (Arabic version previously validated among war affected Palestinian children), SCAS Spence children’s anxiety scale (validated in Arabic but not for refugee children) | Mod |
| Kalantari et al., 2012 | Omega – J. of Death and Dying | Efficacy of Writing for Recover on Traumatic Grief Symptoms of Afghani Refugee Bereaved Adolescents: A Randomized Controlled Trial | RCT | 64  (Intervention = 32  Control = 32) | NA | PTSD | TGIC Traumatic grief inventory for children (validated in Farsi, but nor for refugee children) | Mod |
| Ooi et al., 2016 | Front. In Psychol. | The efficacy of a group cognitive behavioural therapy for war-affected young migrants living in Australia: A cluster randomized controlled trial | RCT (cluster) | 82  (Intervention = 45  Control = 37) | Depression | Parent rated Emotional and behavioural problems, PTSD | DSRS Birleson Depression Self-Rating Scale (widely used to assess refugee children), CRIES-13 Childrens Revised Impacts of Events Scale (developed for war effected children), Parent version SDQ Strengths and Difficulties Questionnaire (English version not specifically validated for refugee populations) | Mod |
| Pfeiffer & Goldbeck, 2017 | Int. Soc. For Trauma. Stud. | Evaluation of trauma-focused group intervention for unaccompanied young refugees: a pilot study | Pre-post | 36 | NA | PTSD | CATS Child and Adolescent Trauma Scale (widely used in refugee populations but translated versions seen to have lower reliability) | Ser |
| Quinlan et al, 2016 | The Arts in Psychother. | Evaluation of a school-based creative arts therapy programme for adolescents from refugee backgrounds | Pre-post (inequivalent control) | 42  (Arts = 22  Control = 20) | Depression | Anxiety, Teacher rated Emotional and behavioural problems | HSCL-25 Hopkins symptom checklist Depression and Anxiety subscales (widely used to assess refugee children and validated for use in several cultures), Teacher version SDQ Strengths and difficulties questionnaire (English version not specifically validated for refugee populations) | Ser |
| Thabet et al., 2005 | Eur. Child and Adoles. Psychiatry | Group crisis intervention for children during ongoing war conflict | Cluster pre-post (3 arm, inc. control) | 111  (Debriefing = 47, Psychoeduc. = 22  Control = 42) | Depression | PTSD | CDI Child Depression Inventory (validated Arabic version, but not specifically for refugees), CPTSD-RI Child Post Traumatic Stress Reaction Index (Arabic version previously validated among war affected Palestinian children), | Mod |
| Tubbs Dolan et al., 2022 | Am. Educ. Res. J. | Supporting Syrian Refugee Children’s Academic and Socio-Emotional Learning in National Education Systems: A Cluster Randomized Controlled Trial of Nonformal Remedial Support and Mindfulness Programs in Lebanon | RCT cluster (3 arm) | 4784  (Healing Classroom = 1,858  Healing Classroom + Mindfulness = 1,834  Control = 1092) | Depression | NA | MFQ Moods and feeling Questionnaire (validated Arabic version, but not specifically for refugees) | Mod |
| Ugurlu et al., 2016 | Vulnerable Child. And Youth Stud. | An art therapy for symptoms of post-traumatic stress, depression and anxiety among Syrian refugee children | Pre-post | 63  (30 randomly chosen for post-test) | Depression | Anxiety, PTSD | CDI Child Depression Inventory (validated Arabic version, but not specifically for refugees), State-Trait Anxiety Scale (validated Arabic version, but not specifically for refugees), UCLA PTSD Index Parent report (good convergent validity for refugee youth, Arabic version) | Ser |

*Note.* Journal names are abbreviated according to ISSN List of Title Word Abbreviations, NA = not applicable, IPT-G = Interpersonal Therapy – Group, RoB = aggregate risk of bias for individual studies assessment according to Cochrane tools, Ser = Serious, Mod = Moderate, CBT = Cognitive Behavioural Therapy, Psychoeduc. = psychoeducation
