# Supplementary material for: The efficacy of psychological prevention, and health promotion interventions targeting psychological health, wellbeing or resilience among forced migrant children and youth: a systematic review and meta-analysis
Source: Eur Child Adolesc Psychiatry. 2024 Apr 16;34(1):123–40. doi: 10.1007/s00787-024-02424-8 (PMC11805832; doi:10.1007/s00787-024-02424-8)
Supplement: Supplementary file 8 — Supplementary file8 (DOCX 26 KB) [file 787_2024_2424_MOESM8_ESM.docx]

Supplementary Information 8

**The efficacy of psychological prevention, and health promotion interventions targeting psychological health, wellbeing or resilience among forced migrant children and youth: a systematic review and meta-analysis**

**European Child and Adolescent Psychiatry**

Clover Jack Giles ^1^, Maja Västhagen ^2^, Livia Van Leuven ^2^,

Anna Edenius^3^, Ata Ghaderi ^2^, Pia Enebrink ^2^

^1^ School of Behavioural, Social and Legal Sciences, Örebro University, Örebro, Sweden

^2^ Department of Clinical Neuroscience, Karolinska Institutet, Stockholm, Sweden

^3^ Department of Medicine, Karolinska Institutet, Stockholm, Sweden

*Corresponding author:*

Clover Jack Giles (CJG)

[clover.giles@oru.se](mailto:clover.giles@oru.se)

# Supplementary Information 8: Participant Characteristics

**Table 2**

*Participant Characteristics*

| Author, year | Population | | Ethnicity/country of origin | Mother-tongue | | | Current location (Mean time) and country income level * | With parents | Age (Mean) | Gender (% girls)** |
| --- | --- | --- | --- | --- | --- | --- | --- | --- | --- | --- |
| Bolton et al., 2007 | Internally displaced | | Ugandan Acholi | Acholi | | | Encampments in Uganda (*M* = 5.20 years), Low | NI | 14-17 (15) | Total = 57.00, IPT- Group = 57.00, Creative play = 58.00, Control = 57.00 |
| Cardeli et al., 2020 | Refugees | | Bhutanese | Nepali | | | New England, USA (*M* = 2.55, *R* = 0-6 years), High | NI | 11-15 (12.94) | Total = 46.00 |
| Doumit et al., 2020 | Refugees | | Syrian | Arabic | | | Suburban district in Lebanon (< 5 years), Lower middle | NI | 13-17  (14.22) | Total = 51.60 |
| Ehntholt et al., 2005 | Refugees and asylum seekers | Albanian (Kosovo, 11), Afghani (1), Kurdish (Türkiye, 3), Sierra Leonian (10), Somali (1) | | | Albani, Farsi, Krio, Kurdish, Somali | London, UK (ca. 2 years), High | | Yes | 11-15  (12.89) | Total = 35.00, CBT = 33.33, Control = 36.36 |
| Foka et al., 2021 | Refugees and displaced | Afghani (13), Kurdistan (1), Lebanon (1), Sateless (2), Syrian (41), Unspec. (2) | | | Arabic and Farsi | Encampments in Greece (no duration of stay reported), High | | Yes | 7-14 (10.75) | Total = 63.90, Strengths for the Journey = 78.8, Control = 52.6 |
| Fox et al., 2005 | Refugees | Vietnamese, Cambodian | | | Vietnamese and Cambodian | USA (no duration of stay reported), High | | Yes | 6-16  (10) | Total = 56.00 |
| Garoff et al., 2018 | Refugees and asylum seekers | Afghani (7), Iraqi (2), Unspecified (9) | | | Arabic and Farsi | Asylum accommodations in Finland, High  (*R* = 2-42 months). | | No | 9-17  (15.08) | Total = 11.11 |
| Gormez et al., 2017 | Refugees | Syrian | | | Arabic and Turkish | Istanbul, Türkiye (< 2 years), Upper middle | | Yes | 10-15  (12.41) | Total = 62.50 |
| Kalantari et al., 2012 | Refugees | Afghani | | | Farsi | Iran (no duration of stay reported), Lower middle | | Both | 12-18  (14.80) | Total = 55.00, Writing = 55.17, Control = 50.00 |
| Ooi et al., 2016 | Forcibly Displaced | Africa, Asia, and the Middle East | | | Most spoke Arabic, Farsi, Kirundi, Karen, or Burmese | Australia (*M* = 2.36 years, *R* = 1-7 years), High | | NI | 10-17  (12.59) | Total = 35.37, Teaching Recovery Techniques = 27.00, Control = 46.00 |
| Pfeiffer & Goldbeck, 2017 | Refugees | Afghani (14), Albani (2), Eritrean (3), Gambian (2), Ghanan (1), Iraqi (1), Nigerian (1), Pakistan (2), Somalia (1), Sudan (1), Syria (1) | | | NI | Child welfare accommodation in Germany (*M* = 23 months, *R* = 2 to 23), High | | No | 14-18 (16.7) | 0 |
| Quinlan et al, 2016 | Refugees | Africa, East Asia and the Middle East | | | NI | Australia (*M* = 11 months), High | | Yes | Ca. 13-17 (15.5) | Total = 59.50, Arts = 63.64, Control = 55.00 |
| Thabet et al., 2005 | Refugees and internally displaced | Palestinian | | | Arabic | Encampments on the Gaza Strip (no duration of stay), Upper Middle | | Yes | 9-15 (12.10) | Total = 46.85, Debriefing, 31.91, Psychoeducation 100,  Control, 33.33 |
| Tubbs Dolan et al., 2022 | Refugees | Syrian | | | Arabic | Lebanon (42% in tented encampments, remainder in houses) (*M* = 2,6 years, *R* = 0 – 6 years), Lower middle | | NI | 5-15 (8.89) | Total = 49.00, NI for each condition |
| Ugurlu et al., 2016 | Refugees | | Syrian | Arabic | | | Working-class district in Istanbul, Türkiye. (76% ≤ 1 years), Upper middle | Yes | 7-12 (9.16) | Total = 46.00 |

*Note.*  NI = no information reported in study, CBT = Cognitive Behavioural Therapy

*Income level according to the World Bank member countries list of economies, 2022-2023

**Total girls n=2847 (49.59%).
