# Supplementary material for: The efficacy of psychological prevention, and health promotion interventions targeting psychological health, wellbeing or resilience among forced migrant children and youth: a systematic review and meta-analysis
Source: Eur Child Adolesc Psychiatry. 2024 Apr 16;34(1):123–40. doi: 10.1007/s00787-024-02424-8 (PMC11805832; doi:10.1007/s00787-024-02424-8)
Supplement: Supplementary file 9 — Supplementary file9 (DOCX 29 KB) [file 787_2024_2424_MOESM9_ESM.docx]

Supplementary Information 1

**The efficacy of psychological prevention, and health promotion interventions targeting psychological health, wellbeing or resilience among forced migrant children and youth: a systematic review and meta-analysis**

**European Child and Adolescent Psychiatry**

Clover Jack Giles ^1^, Maja Västhagen ^2^, Livia Van Leuven ^2^,

Anna Edenius^3^, Ata Ghaderi ^2^, Pia Enebrink ^2^

^1^ School of Behavioural, Social and Legal Sciences, Örebro University, Örebro, Sweden

^2^ Department of Clinical Neuroscience, Karolinska Institutet, Stockholm, Sweden

^3^ Department of Medicine, Karolinska Institutet, Stockholm, Sweden

*Corresponding author:*

Clover Jack Giles (CJG)

[clover.giles@oru.se](mailto:clover.giles@oru.se)

# Supplementary Information 9: Intervention Characteristics

| Author and year | Level of prevention | Intervention | Classification | Content | Cultural tailoring | Group size | Length | Language | Leader | Setting |
| --- | --- | --- | --- | --- | --- | --- | --- | --- | --- | --- |
| Bolton et al., 2007 | Indicated: non-clinical setting, no diagnosis | Creative Play | Play therapy | Strengthening resilience and emotional expression through creative activities, such as songs, arts, role plays, music, sports, games and led discussions and debates. | The manual was adapted for local use. Mixed gender groups | 25-30 | 16 weeks, 16 sessions, 24-32 hours | NI (Acholi) | 1 male and 1 female facilitator from War Child Holland | Refugee camp |
| Bolton et al., 2007 | Indicated: non-clinical setting, no diagnosis | Group Interpersonal Therapy | Interpersonal Therapy | Identifying interpersonal problems and practising appropriate interpersonal skills. | Same gender groups | 6-8 | 16 weeks, 16 sessions, 24-32 hours | NI  (Acholi) | 1 x facilitator with 2 weeks onsite training | Refugee camp |
| Cardeli et al., 2020 | Selected: part of a larger stepped care program | TST-R Trauma Systems Therapy for Refugees | Trauma systems therapy | Learning through games, role-plays, and experiential exercises, cross cultural discussions, emotional regulation, building self-esteem. | A cultural broker mediates cross-cultural differences as they occur. | NI | 12 weeks, 12 sessions, 12 hours | Eng. And Nepal | Mental health clinician plus cultural broker | After-school activity |
| Doumit et al., 2020 | Universal/ Selected | COPE Creating Opportunities for Patient Empowerment | CBT | Sessions were held in mornings with breakfast served afterwards. Sessions included discussions, skills training and psychoeducation on thoughts, emotions and behaviour, stress and coping, problem solving, and communication. | NI except live translation from Eng. Manual. The program was developed and has been demonstrated to be efficacious for depressed adolescents and adolescents in the general population. |  | 7 weeks, 7 session, 7 hours 7 homework | Arabic | Study PI and a trained therapist | Community centre |
| Ehntholt et al., 2005 | Indicated: participants had been exposed to trauma and identified by teachers as in need of intervention | School based trauma focused CBT | Trauma focused CBT | Sessions included exposure, psychoeducation, cognitive restructuring, relaxation techniques, sleep hygiene, behavioural activation. | NI | 7-8 | 6 weeks, 6 sessions, 6 hours | Eng. | Trainee clinical psychologist | School |
| Foka et al., 2021 | Promotive/ Selected: non-clinical | Strengths for the journey | Positive Psychology Intervention | Themed sessions focusing on positive thoughts and emotion, future planning and mindfulness. | Developed on experience of working with refugee youth in camps in Lesvos, with input from service providers. Mixed gender groups, but arranges by age where possible | 6-17 | 1 week, 6 sessions, 12 hours | Arabic and Farsi | Trained leader plus interpreter | Refugee camp |
| Fox et al., 2005 | Indicated: sub-clinical to clinical symptoms | School based program for Southeast Asian Refugee children with depressive symptoms | CBT and skills training | Storytelling, arts, identifying strengths, role-play, psychoeducation and homework. | Developed for Southeast Asian participants with elements of cultural and tradition expression in the program. | NI | 8 weeks, 8 sessions, 8 hours | NI (English and mother tongue) | Bilingual teachers and school nurses | School |
| Garoff et al., 2018 | Universal/ Selected: part of a larger stepped care program | NI | Inspired by Trauma focused CBT | Sessions included psychoeducation, brief ethnographic interview about well-being (identifying important themes), discussion of topics important for individual participants (e.g., sleep, intimate relationships, emotions, pain) and a sociogram coping resource exercise. | Newly developed program targeting unaccompanied youth in Finland. Live translation during implementation. | 1-7 | 10 weeks, 10 sessions, 15 hours | NI (Finnish, Arabic and Dari) | Trained accommodation staff, a mental health professional, and in some cases a translator | Asylum accommodation |
| Gormez et al., 2017 | Indicated: sub-clinical to clinical symptoms | Group based CBT | CBT | All sessions start with a session specific warmup, thereafter psychoeducation, discussions, skills training (problem solving skills, narrative exposure and cognitive restructuring, emotion regulation skills, relaxation techniques) and instructional games. | Needs based tailoring by Arabic speaking leaders. | 8-10 | 8 weeks, 8 session, 7.33-12 hours | Arabic | Teachers | School |
| Kalantari et al., 2012 | Indicated: symptoms of traumatic grief | Writing for Recovery | Written trauma focused CBT | Written exposure and cognitive restructuring. Writing about own thoughts and feeling, about others in similar positions, and what they have learnt from the exercises. | No specific tailoring as writing allows for cultural expression. | NA | 3 days, 6 sessions, 1.5 hours | Farsi | NI | School |
| Ooi et al., 2016 | Indicated: mild to moderate symptoms of PTSD | Teaching Recovery Techniques (no parent sessions) | Trauma focused CBT | Sessions themed around elements of PTSD, group discussions, development of self-coping, exposure, and relaxation strategies. | Sessions were adapted in lengths and number to fit within the Australian school schedule. | 4-10 | 8 weeks, 8 sessions, 8 hours | English | Master and PhD level psychology students | School |
| Pfeiffer & Goldbeck, 2017 | Indicated: mild to moderate symptoms of PTSD | Mein Weg (My Way) Trauma Focused group CBT | Trauma focused CBT | Psychoeducation, relaxation, exposure and gradual desensitization, and cognitive restructuring. | Adapted to suit a child welfare setting and with visual aides to compensate for language skills. | 2-6 | 6 weeks, 6sessions, 6 hours | German | Social workers | Child welfare institutions/refugee housing |
| Quinlan et al, 2016 | Indicated: intervention groups was considered in need of psychosocial support | HEAL Expressive Arts Therapy | Expressive Arts Therapy | Arts and music activities designed to promote resilience, cultural and emotional awareness, empowerments, social competence, and positive relationships. | Based on an existing program for culturally and lingually diverse populations in Australia. No specific cultural tailoring described, but sessions were held in culturally and gender segregated groups. | NI | 10 weeks, at least 1, 1-hour session per week, | NI (English and/or translators) | Registered arts and music therapists | Schools |
| Thabet et al., 2005 | Indicated: moderate to severe symptoms of PTSD, no diagnosis | Crisis Incident Stress Management | Psychological Debriefing | Psychological debriefing. | No information about cultural tailoring was provided but intervention groups were gender and age segregated. | 15-32 | 7 weekly sessions | NI (Arabic) | Psychologists and social workers | Refugee camp summer school |
| Thabet et al., 2005 | Indicated: moderate to severe symptoms of PTSD, no diagnosis | Teacher education | Psychoeducation | Psychoeducation about trauma. | No information about cultural tailoring was provided but intervention groups were gender and age segregated | 15-32 | 4 weekly sessions | NI (Arabic) | Teachers | Refugee camp summer school |
| Tubbs Dolan et al., 2022 | Universal/ Selected | Socio-emotional learning based nonformal remedial support: Tutoring in a Healing Classroom | Social Emotional learning | Socioemotional learning targeting self-worth, sense of belonging, sense of self-control and positive special relationships integrated into 40 min blocks of core curriculum subjects (Maths, Arabic, English and French) with 10 min recreation breaks between subjects. | NI | NI | 16 weeks, 48 sessions, 40 hours (inc. 24 hours recreation) | Arabic, English, or French | Teachers | Classrooms |
| Tubbs Dolan et al., 2022 | Universal/ Selected | Socio-emotional learning based nonformal remedial support: Tutoring in a Healing Classroom plus Mindfulness. | Social Emotional learning plus mindfulness | Socioemotional learning targeting self-worth, sense of belonging, sense of self-control and positive special relationships integrated into 40 min blocks of core curriculum subjects (Maths, Arabic, English and French) with 10 min mindfulness between subjects. | NI | NI | 6 weeks, 48 sessions, 40 hours (inc. 24 hours mindfulness) | Arabic, English, or French | Teachers | Classrooms |
| Ugurlu et al., 2016 | Selected: offered to all Syrian refugee children with war experiences | Trauma therapy informed, Art therapy-based Skills for Psychological Recovery Program. | Trauma informed arts therapy with developmental perspectives | Music, dance, and art therapy activities promoting problem solving skills, emotion regulation, helpful thinking, and healthy social connections. | Delivered in a culturally sensitive manner and in age segregated groups: 7-8, 9-10 and 11-12 years. | NI | 5 days, 3 session per day | Arabic | Art therapists and volunteer translators | NI (in community) |

*Note.* NI = no information reported in study, () data presented in parenthesis is presumed from study context but not explicitly stated in manuscript.
