# Supplementary material for: The efficacy of psychological prevention, and health promotion interventions targeting psychological health, wellbeing or resilience among forced migrant children and youth: a systematic review and meta-analysis
Source: Eur Child Adolesc Psychiatry. 2024 Apr 16;34(1):123–40. doi: 10.1007/s00787-024-02424-8 (PMC11805832; doi:10.1007/s00787-024-02424-8)
Supplement: Supplementary file 10 — Supplementary file10 (DOCX 51 KB) [file 787_2024_2424_MOESM10_ESM.docx]

Supplementary Information 10

**The efficacy of psychological prevention, and health promotion interventions targeting psychological health, wellbeing or resilience among forced migrant children and youth: a systematic review and meta-analysis**

**European Child and Adolescent Psychiatry**

Clover Jack Giles ^1^, Maja Västhagen ^2^, Livia Van Leuven ^2^,

Anna Edenius^3^, Ata Ghaderi ^2^, Pia Enebrink ^2^

^1^ School of Behavioural, Social and Legal Sciences, Örebro University, Örebro, Sweden

^2^ Department of Clinical Neuroscience, Karolinska Institutet, Stockholm, Sweden

^3^ Department of Medicine, Karolinska Institutet, Stockholm, Sweden

*Corresponding author:*

Clover Jack Giles (CJG)

[clover.giles@oru.se](mailto:clover.giles@oru.se)

# Supplementary Information 10: Statistical Results of Individual Studies

***Secondary outcomes***

Results for variables reported in multiple individual studies are presented in tables. Between group results for depression are presented in Table 1. Within group results for depression are presented in Table 2. Results for PTSD and traumatic grief are presented in Table 3. Results for Anxiety are presented in Table 4. Results for Behavioural and Emotional difficulties are presented in Table 5.

Internalizing and externalizing behaviours were reported using the Hopkins Symptom Checklist 37A [1] in one study (Oii et al., 2016). The internalizing subscale measures depression and anxiety and a time X group Generalized Linear Mixed Model indicated no effect of the intervention from pre- to post-test *(F*(1,155) = 0.03, partial *η2* = 0.00. The externalizing subscale measures behaviours related to Conduct disorder, Oppositional defiant disorder, and Substance abuse. A time X group Generalized Linear Mixed Model indicated no effect of the intervention from pre- to post test (*F*(1,155) = 0.25, partial η2 = 0.01).

**Table 1**

*Between Group Results For Depression (Intervention Vs. Comparator, Randomized Control, or Non-Randomized Control)*

| Author and year | RCT | N in analysis | Pre Mean (SD) | Post Mean (SD) | Statistical analysis reported in study | Results reported in study | Effect Direction |
| --- | --- | --- | --- | --- | --- | --- | --- |
| Bolton et al., 2007      IPT-Group      Creative Play      Wait List Control Group | yes | 105  105  104 | 43.5 (10.1)  44.2(11.2)  44.2 (10.8) | 27.8 (17.2)  40.6 (15.7)  37.3 (15.9) | Difference in adjusted mean score change compared to control, *p,* 95% CI | 9.79, SE = 4.15, 95% CI [1.66-17.93];  -2.51, SE= (NI) 95% CI [-11.42-6.39] | *-*  *- ns* |
| Ehntholt et al., 2005      Trauma Focused CBT       NR Wait List Control | no | 15  11 | 12.33 (4.7)  12.0 (5.37) | 11.67 (3.62)  13.0 (6.57) | ANCOVA (interventions vs control) | No significant difference between groups were found. | *+ ns* |
| Foka et al., 2021        Strengths for the Journey       QR Wait List Control | no | 22^a^  31^b^ | 17.09 (7.06)  17.13 (6.86) | 4.4 (4.39)  19.04 (5.96) | ANOVA, partial *η2, p* | *F*(1,31) = 62.14, partial *η2* = 0.67*, p* = <0.001 | *+* |
| Ooi et al., 2016       Teaching Recovery Techniques        Wait List Control Group | yes | 45  37 | 10.96 (5.26)  9.17 (4.61) | 8.68 (5.48)  8.81 (4.8) | Time X group ANOVA, partial *η2, t*-tests, *p,* 95% CI | *F*(1,155) = 5.20, partial *η2* = 0.07, *p =* 0.024, *t*(155), 3.84, *p* =<0.001 | *+* |
| Quinlan et al., 2016      Arts Therapy       Inequivalent passive control | no | 22  20 | 1.53 (0.61)  1.67 (0.53) | NI  NI | Mean difference  Mean difference  *t*-tests, Cohen’s *d, p,* 95% CI | -0.17 (0.71)  0.23 (0.46)  *t*(40) = -0.32, *p* = 0.75, 95% CI [-0.44-0.32], *d* = 0.1 | *+ns* |
| Thabet et al., 2005      Crisis intervention       Psychoeducation       NR Wait List Control | no | 47  22  42 | 12.9 (8.1)  11.9 (7.2)  14.4 (7.8) | 14.3 (9.7)  12.1 (7.6)  13.0 (7.5) | ANOVA (between group), *p* | *F*(2, N=111) = 1.45, *ns* | *-* |
| Tubbs Dolan et al., 2022      Healing Classrooms        Healing Classrooms + Mindfulness       Treatment As Usual Control | yes | 1761  1754  1083 | NI  NI  NI | NI  NI  NI | Unadjusted effect size (unspecified – described in contact with author as similar to Cohen’s *d), SE, p* | ES = 0.0, *SE* = 0.01, *p* = 0.920;  ES = 0.064, *SE* = 0.105, *p* = 0.540 | *+ns*  *+ns* |

*Note*. RCT = Randomized Controlled Trial, NR = Non-randomized, QR = Quasi-randomized, NI = no information, *ns* = non-significant

^a^ 15 at post

^b^ 25 at post

**Table 2**

*Within Group Results for Depression*

| Author and year | In meta-analysis | | N | Pre Mean (SD) | Post Mean (SD) | Statistical analysis reported in study | Results | Effect Direction |
| --- | --- | --- | --- | --- | --- | --- | --- | --- |
| Bolton et al., 2007    IPT-Group     Creative Play     Randomized WL Control | Yes | 105  105  104 | | 43.5 (10.1)  44.2(11.2)  44.2 (10.8) | 27.8 (17.2)  40.6 (15.7)  37.3 (15.9) | Mean difference | 15.7 (17.0)  3.6 (18.2)  6.9 (16.9) | *+*  *+*  *+* |
| Cardeli et al., 2020 | Yes | 31 | | 10.26 (3.19) | 10.19 (2.93) | *t*-tests, Cohen’s *d, p* | *t*(30) = 0.108, *p* = 0.92, *d* = 0.02 | *+ns* |
| Doumit et al., 2020      Whole sample       Subsample ^a^ | Yes | 31  19 | | 6.35 (3.25)  8.50 (2.30) | 4.90 (2.38)  5.44 (2.40) | *t*-tests, *p,* Cohen’s *d,*  *t*-tests, *p,* Cohen’s *d,* | *t*(30) = 2.35, *p* = 0.025, *d* = 0.42  *t*(18) = 3.97, *p* = 0.001, *d* = 0.94 | *+*  *+* |
| Ehntholt et al., 2005        Trauma Focused CBT        NR WL Control | Yes | 15  11 | | 12.33 (4.7)  12.0 (5.37) | 11.67 (3.62)  13.0 (6.57) | *p* of *t*-tests of withing group change | *ns*  *ns* | *+*  *-* |
| Foka et al., 2021        Strengths for the Journey        QR WL Control | Yes | 22^b^  31^c^ | | 17.09 (7.06)  17.13 (6.86) | 4.4 (4.39)  19.04 (5.96) | NI  NI | NI  NI | *+ ns*  *- ns* |
| Fox et al., 2005 | Yes | 58 | | 10.38 (4.6) | 6.15 (3.8) | *t*-tests, *p* | *t*(57) = 4.89, *p* = <0.001 | *+* |
| Ooi et al., 2016       Teaching Recovery Techniques       Randomized WL Control | Yes | 45  37 | | 10.96 (5.26)  9.17 (4.61) | 8.68 (5.48)  8.81 (4.8) | *t*-tests, *p*  *t*-tests, *p* | *t*(155) = 3.84, *p* = <0.001  *t*(155) = 0.47), *p* = 0.643 | *+*  *+ns* |
| Quinlan et al, 2016        Arts Therapy        Inequivalent passive control | Yes | 22  20 | | 1.53 (0.61)  1.67 (0.53) | NI  NI | Mean difference (SD)  Mean difference (SD) | -0.17 (0.71)  0.23 (0.46) | *+ ns*  *- ns* |
| Thabet et al., 2005       Crisis intervention       Psychoeducation        NR WL Control | Yes | 47  22  42 | | 12.9 (8.1)  11.9 (7.2)  14.4 (7.8) | 14.3 (9.7)  12.1 (7.6)  13.0 (7.5) | Wilcoxon test of change, *p*  Wilcoxon test of change, *p*  Wilcoxon test of change, *p* | *z* = 0.89, *ns*  *z* = 0.34, *ns*  *z* = 01.2, *ns* | *-*  *-*  *+* |
| Tubbs Dolan et al., 2022      Healing Classrooms       Healing Classrooms + Mindfulness  Randomized TAU Control | No | 1761  1754  1083 | | NI  NI  NI | NI  NI  NI | NI  NI  Unadjusted change estimates; *b, SE, p, q* | NI  NI  *b* = 10.25, *SE* = 0.09, *p* = 0.006,  *q* = 0.010 | *+* |
| Ugurlu et al., 2016 | Yes | 30 | | 9.97 (1.01) | 6.0 (4.54) | *t*-tests, Hedges *g, p,* 95% CI | *t*(29) = 3.955, *p* = <0.05, *g* = 0.72, 95% CI [0.20-1,24] | *+* |

*Note.* WL = Wait list, NR = Non-randomized, QR = Quasi-randomized, TAU = Treatment as usual, NI = no information, *ns* = non-significant, ^a^ Subsample of participants with moderate to severe symptoms, ^b^ 15 at post, ^c^ 25 at post

**Table 3**

*Between Group and Within Group Results for PTSD and Traumatic Grief (Intervention Vs. Comparator, Randomized Control, or Non-Randomized Control)*

| Author and year | n | Pre Mean (SD) | Post Mean (SD) | Statistical analysis | Results | Effect  Direction |
| --- | --- | --- | --- | --- | --- | --- |
| Cardeli et al., 2020 | 27 | 21.48 (15.2) | 20.0 (11.44) | *t*-tests, Cohen’s *d, p* | *t*(26) =0.6., *p* = 0.55, *d* = 0.11 | +*ns* |
| Ehntholt et al., 2005         Trauma Focused CBT         NR WL Control | 15  11 | 39.8 (8.4)  38.55 (8.37) | 33.8 (9.71)  42.18 (9.38) | *t-*tests, *p*  *t-*tests, *p*  ANCOVA (group) | *t*(14) = 2.934, *p* = 0.011  *t*(10) = -2.003, *p* = 0.073  *F*(1,23) = 10.955, *p* = 0.003 | +  -*ns*  + |
| Garoff et al., 2018 | 10 | 32.9 (9.5) | 36.8 (10.89) | *t*-tests, *p,* 95% CI | *t*(9) = 1.30, *p* = 0,23, 95% CI [-2.88, 10.68] | -*ns* |
| Gormez et al., 2017 | 30 | 23.9 (12.76) | 17.63 (13.64) | t-tests, *p* | *t*(29) = 2.72, *p* = 0.011 | + |
| Kalantari et al., 2012 ^a^         Writing for Recovery         Randomized Passive Control | 29  32 | 56.3 (11.6)  49.9 (13.3) | 44.9 (12.6)  53.9 (14.2) | *p*  *P*  ANCOVA (group), partial *η2, p* | *p* = < 0.001  *ns*  *F*(60) = 12.97, partial *η2* = 0.19, *p =* 0.001 | +  -*ns*  + |
| Ooi et al., 2016       Teaching Recovery Techniques         Randomized WL Control | 39  37 | 23.02 (10.51)  17.92 (11.86) | 15.88 (9.58)  15.68 (8.84) | Reliable change  Reliable change  Time X group ANOVA, partial *η2,* *p* | 21% improved, 74% no change, 5 % deteriorated  11% improved, 86% no change, 5 % deteriorated  *F*(1,154) = 3.09, partial *η2* = 0.04, *p =* 0.081 | +NI  +NI  +*ns* |
| Pfeiffer & Goldbeck, 2017 | 29 | 27.58 (7.88) | 20.67 (6.3) | *t*-tests, Cohen’s *d, p,* 95% CI | *t*(28) = 4.172, *p* = 0.001, 95% CI [3.68, 10.20], *d* = 0.97 | + |
| Thabet et al., 2005         Crisis intervention         Psychoeducation         NR WL Control | 47  22  42 | 29.5 (11.9)  33.0 (7.9)  29.6 (11.4) | 28.3 (13.4)  32.2 (8.7)  31.0 (12.6) | Wilcoxon test of change, *p*  Wilcoxon test of change, *p*  Wilcoxon test of change, *p*  ANOVA (group), *p* | *z* = 0.48, ns  *z* = 0.16, ns  *z* = 0.73, ns  *F*(2, N=111) = 0.54, *ns* | +*ns*  +*ns*  -*ns*  +*ns* |
| *Parent rated PTSD*  Ugurlu et al., 2016 | 25 | 29.8 (10.5) | 15.32 (9.59) | *t*-tests, Hedge’s *g, p,* 95% CI | *t*(24) = 5.45*, p* = <0.05, *g* = 1.00, 95% CI [0.45, 1,52] | + |

*Note.* NR = Non-randomized, WL = Wait list, NI = no information, *ns* = non-significant

^a^ Outcome = Traumatic Grief

**Table 4**

*Within and Between Group Results for Anxiety (Intervention Vs. Comparator, Randomized Control, or Non-Randomized Control)*

| Author and year | n in analysis | Pre Mean (SD) | Post Mean (SD) | Statistical analysis | Results | Effect Direction |
| --- | --- | --- | --- | --- | --- | --- |
| Doumit et al., 2020       Whole sample        Subsample ^a^ | 31  11 | 4.61 (2.95)  7.73 (2.19) | 3.61 (2.51)  5.18 (2.89) | *t*-tests, *p,* Cohen’s *d*  *t*-tests, *p,* Cohen’s *d* | *t*(30) = 2.35, *p* = 0.025, *d* = 0.42  *t*(10) = 3.82, *p* = 0.003, *d* = 1.15 | +  + |
| Ehntholt et al., 2005       Trauma Focused CBT       NR WL Control | 15  11 | 16.87 (7.22)  16.18 (6.57) | 14.67 (7.12)  18.91 (6.04) | *t-*tests, *p*  *t-*tests, *p*  ANCOVA (group) | *t*(14) = 1.581, *p* = 0.136  t(10) = -2.042, *p* = 0.068  *F*(1,23) = 6.495, *p* = 0.018 | +*ns*  *-ns*  *+* |
| Gormez et al., 2017 | 32 | 53.28 (13.78) | 40.38 (20.59) | t-tests, *p* | *t*(31) = 3.73, *p* = 0.001 | + |
| Quinlan et al., 2016      Arts Therapy       Inequivalent passive control | 22  20 | 1.17 (0.35)  1.34 (0.49) | NI  NI | *t*-tests,  Mean difference (SD)  Mean difference (SD)  Between group *t*-test, *p*, 95% CI, Cohen’s *d* | 0 (0.36)  0.07 (0.47)  *t*(40) = -0.53, *p* = 0.60, 95% CI [-0.33,0.19], *d* = 0.17 | NA  NA  -*ns* |
| Ugurlu et al., 2016       Trait anxiety       State anxiety | 25  25 | 36.92 (6.96)  30.52 (8.31) | 30.28 (7.39)  28.84 (7.15) | *t*-tests, *p,* Hedges *g,* 95% CI  *t*-tests, *p* | *t*(24) = 4.366, *p* = <0.05, *g* = 0.80, 95% CI [0.27, 1.32]  *t*(25) == 1.010, p >0.05 | +  +*ns* |

*Note.* CBT = Cognitive Behavioural Therapy, NR = Ron-randomized, WL = Wait List, NI = No information, NA = not applicable, *ns* = non-significant

^a^ Subsample of participants with moderate to severe symptoms,

**Table 5**

*Within and Between Group Results for SDQ*

| Author and year | Rater | N in analysis | Pre Mean (SD) | Post Mean (SD) | Statistical analysis | Results | Effect Direction |
| --- | --- | --- | --- | --- | --- | --- | --- |
| Ehntholt et al., 2005      Trauma Focused CBT | Teacher | 10 | 9.20 (7.76) | 5.40 (4.35) | Wilcoxon signed rank test, *p* | *Z* = -2.207, *p* = 0.027 | +*ns* |
| Garoff et al., 2018 | Caretaker | 12 | 14.42 (5.85) | 14.75 (5.28) | *t*-tests, *p,* 95% CI | *t*(11) = 0.23, *p* = 0.82, 95% CI [-2.80, 3.46] | -*ns* |
| Gormez et al., 2017 | Child | 32 | 18.77 (4.28) | 16.81 (5.41) | t-tests, *p* | *t*(30) = 2.44, *p* = 0.021 | + |
| Ooi et al., 2016      Teaching Recovery Techniques      Randomized Wair List Control | Parent  Parent | 38  32 | 7.34 (3.64)  7.53 (4.24) | 5.83 (2.81)  5.34 (4.03) | Time X group ANOVA, partial *η2, t*-tests, *p,* 95% CI | *F*(1,155) = 0.28, partial *η2* = 0.00, *ns* | -*ns* |
| Quinlan et al., 2016      Arts Therapy  Inequivalent passive control | Teacher  Teacher | 22  20 | 6.53 (6.16)  3.43 (3:20) | -3.97 (5.64) ^a^  -1.52 (2.47) ^a^ | *t*-tests,  Cohen’s *d, p,* 95% CI | *t*(40) = 1.79, *p* = 0.08, 95% CI [-0.31, 5.22],  *d* = 0.57 | +*ns* |

*Note. ns* = non-significant

^a^ = Mean change

**References**

1. Bean, T., et al., *Validation of the multiple language versions of the Hopkins Symptom Checklist-37 for refugee adolescents.* Adolescence, 2007. **42**(165): p. 51-71.
