# Supplementary material for: The efficacy of psychological prevention, and health promotion interventions targeting psychological health, wellbeing or resilience among forced migrant children and youth: a systematic review and meta-analysis
Source: Eur Child Adolesc Psychiatry. 2024 Apr 16;34(1):123–40. doi: 10.1007/s00787-024-02424-8 (PMC11805832; doi:10.1007/s00787-024-02424-8)
Supplement: Supplementary file 11 — Supplementary file11 (DOCX 143 KB) [file 787_2024_2424_MOESM11_ESM.docx]

Supplementary Information 11

**The efficacy of psychological prevention, and health promotion interventions targeting psychological health, wellbeing or resilience among forced migrant children and youth: a systematic review and meta-analysis**

**European Child and Adolescent Psychiatry**

Clover Jack Giles ^1^, Maja Västhagen ^2^, Livia Van Leuven ^2^,

Anna Edenius^3^, Ata Ghaderi ^2^, Pia Enebrink ^2^

^1^ School of Behavioural, Social and Legal Sciences, Örebro University, Örebro, Sweden

^2^ Department of Clinical Neuroscience, Karolinska Institutet, Stockholm, Sweden

^3^ Department of Medicine, Karolinska Institutet, Stockholm, Sweden

*Corresponding author:*

Clover Jack Giles (CJG)

[clover.giles@oru.se](mailto:clover.giles@oru.se)

# Supplementary Information 11: Forest Plots of Analysis Within Group Analysis Depression


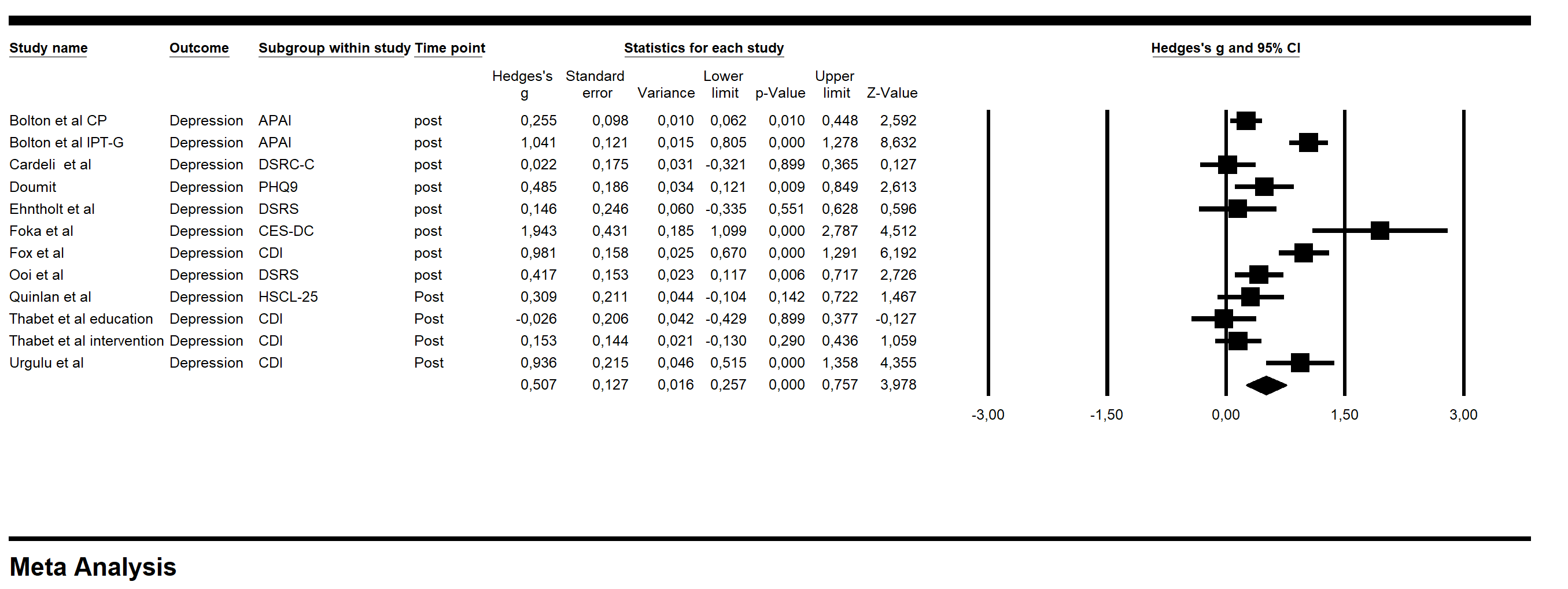


*Note.* CP = Creative Play, IPT-G = Interpersonal Therapy – Group, PS + HCT = Public Schooling plus Tutoring in a Healing Classroom, PS + HCT + M = Public Schooling plus Tutoring in a Healing Classroom plus Mindfulness, APAI = Acholi Psychosocial Assessment Instrument, DSRC-C = Depression Self rating scale for children, PHQ9 = Patient Health Questionnaire 9, DSRS = Birleson Depression Self-Rating Scale – Child version, CES-DC = Center for Epidemiological Studies Depression Scale for Children, CDI = Children’s Depression Inventory, HSCL-25 = Hopkins Symptom Checklist – 25 depression subscale.
