# Supplementary material for: The efficacy of psychological prevention, and health promotion interventions targeting psychological health, wellbeing or resilience among forced migrant children and youth: a systematic review and meta-analysis
Source: Eur Child Adolesc Psychiatry. 2024 Apr 16;34(1):123–40. doi: 10.1007/s00787-024-02424-8 (PMC11805832; doi:10.1007/s00787-024-02424-8)
Supplement: Supplementary file 12 — Supplementary file12 (DOCX 745 KB) [file 787_2024_2424_MOESM12_ESM.docx]

Supplementary Information 12

**The efficacy of psychological prevention, and health promotion interventions targeting psychological health, wellbeing or resilience among forced migrant children and youth: a systematic review and meta-analysis**

**European Child and Adolescent Psychiatry**

Clover Jack Giles ^1^, Maja Västhagen ^2^, Livia Van Leuven ^2^,

Anna Edenius^3^, Ata Ghaderi ^2^, Pia Enebrink ^2^

^1^ School of Behavioural, Social and Legal Sciences, Örebro University, Örebro, Sweden

^2^ Department of Clinical Neuroscience, Karolinska Institutet, Stockholm, Sweden

^3^ Department of Medicine, Karolinska Institutet, Stockholm, Sweden

*Corresponding author:*

Clover Jack Giles (CJG)

[clover.giles@oru.se](mailto:clover.giles@oru.se)

# Supplementary Information 12: Subgroup Analysis of Within-Group Depression

Five subgroup analyses of within group changes in depression were conducted.

**Age.** The forest plot for sub-group analysis of age is presented in Figure 1. The overall effect for age (*M* = < 12 vs. *M* ≥ 12) on depression was significant (*k* = 12, *g* = 0.53*,* 95% CI [0.32, 0.74]*, z* = 4.91, *p* = < .001, *Q*(1) = 10.59), indicating a medium effect size. A large effect size was observed for *M* age < 12 (*k* = 3, *g* = 1.13, 95% CI [0.71,1.56], z = 5.27, *p* = <.001) and small effect size was observed for *M* age ≥ 12 (*k* = 9, *g* = 0.33, 95% CI [0.08, 0.57], z = 2.63, *p* = .009). The analysis indicated that improvements were seen regardless of mean age of participants however, effect sizes were larger for interventions including more pre- or early adolescent participants.

**Figure 1**

*Forest Plot of Sub-Group Analysis of Within-Group Change in Depression by Age*


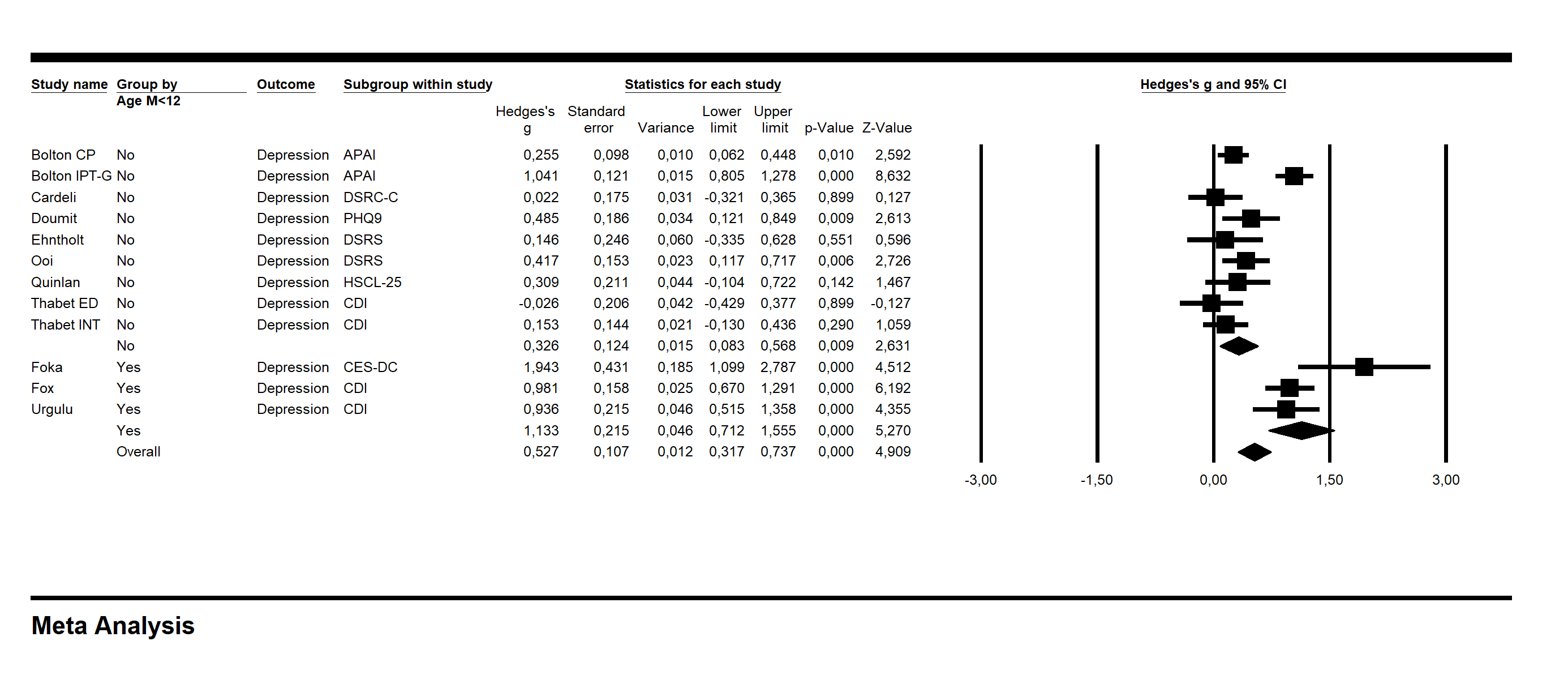


*Note.* CP = Creative Play, IPT-G = Interpersonal Therapy – Group, PS + HCT = Public Schooling plus Tutoring in a Healing Classroom, PS + HCT + M = Public Schooling plus Tutoring in a Healing Classroom plus Mindfulness, APAI = Acholi Psychosocial Assessment Instrument, DSRC-C = Depression Self rating scale for children, PHQ9 = Patient Health Questionnaire 9, DSRS = Birleson Depression Self-Rating Scale – Child version, CES-DC = Center for Epidemiological Studies Depression Scale for Children, CDI = Children’s Depression Inventory, HSCL-25 = Hopkins Symptom Checklist – 25 depression subscale.

**Gender.** The forest plot for sub-group analysis of gender is presented in Figure 2. The overall effect for gender (< 50% girls vs. > 50% girls) on depression was significant (*k* = 12, *g* = 0.45, 95% CI [0.22, 0.68]*, z* = 3.86, *p* = < .001, *Q*(1) = 0.18) indicating a small effect size. A medium effect size was observed for interventions with > 50% girls (*k* = 7, *g* = 0.65, 95% CI [0.28,1.01], z = 3.45, *p* = .001) and small effect size was observed for interventions with < 50% girls (*k* = 5, *g* = 0.33, 95% CI [0.04, 0.61], z = 2.20, *p* = .028). The analysis indicated that improvements were seen regardless of % girls however, effect sizes were larger for interventions including more girls.

**Figure 2**

*Forest Plot of Sub-Group Analysis of Within-Group Change in Depression by Gender*


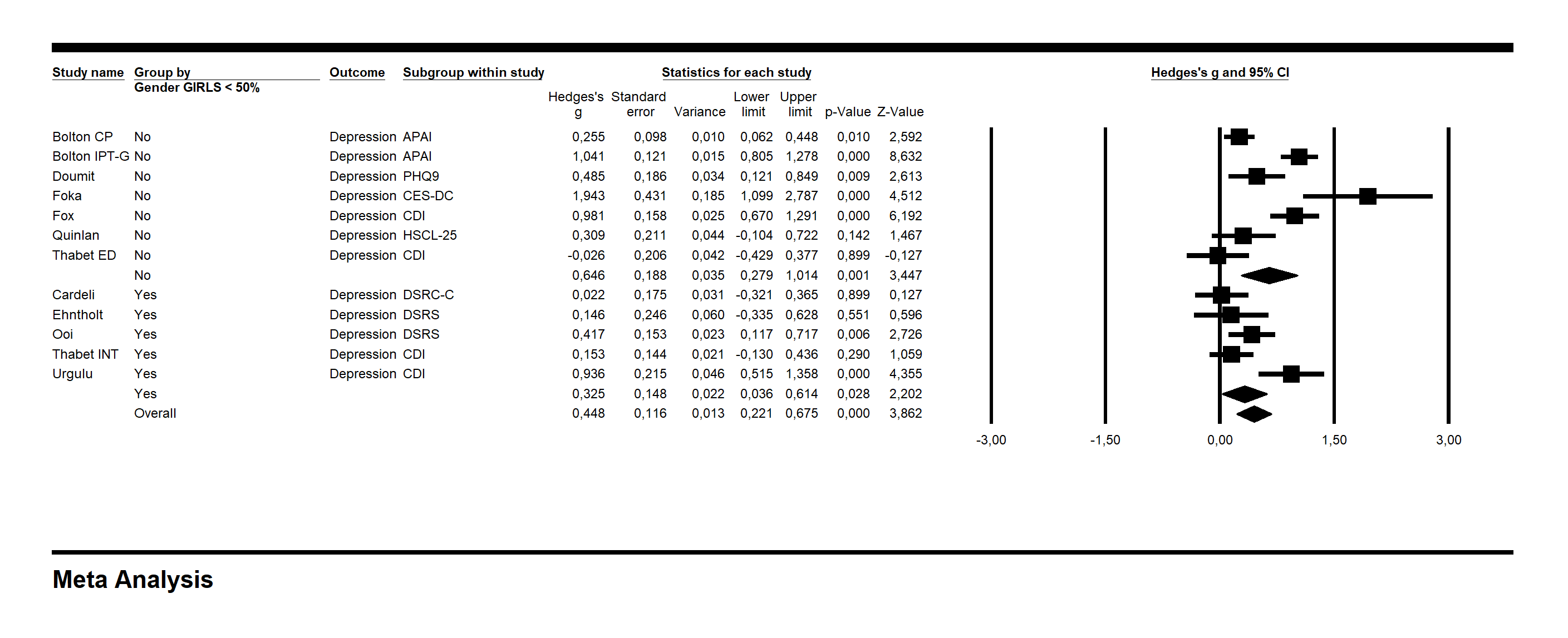


*Note.* CP = Creative Play, IPT-G = Interpersonal Therapy – Group, PS + HCT = Public Schooling plus Tutoring in a Healing Classroom, PS + HCT + M = Public Schooling plus Tutoring in a Healing Classroom plus Mindfulness, APAI = Acholi Psychosocial Assessment Instrument, DSRC-C = Depression Self rating scale for children, PHQ9 = Patient Health Questionnaire 9, DSRS = Birleson Depression Self-Rating Scale – Child version, CES-DC = Center for Epidemiological Studies Depression Scale for Children, CDI = Children’s Depression Inventory, HSCL-25 = Hopkins Symptom Checklist – 25 depression subscale.

**Intervention Context.** The forest plot for sub-group analysis of context is presented in Figure 3. The overall effect for context (intervention implemented in encampments vs. community settings) on depression was significant (*k* = 12, *g* = 0.50*,* 95% CI [0.26, 0.74]*, z* = 4.10, *p* = < .001, *Q*(1) = 0.13), indicating a medium effect size. A medium effect was observed for interventions implemented in camps (*k* = 5, *g* = 0.58, 95% CI [0.10, 1.06], z = 2.36, *p* = .018) and small effects for interventions implemented in community (*k* = 7, *g* = 0.48, 95% CI [0.20, 0.76], z = 3.37, *p* = .001). The analysis indicated that improvements were seen regardless of intervention context.

**Figure 3**

*Forest Plot of Sub-Group Analysis of Within-Group Change in Depression by implementation context*
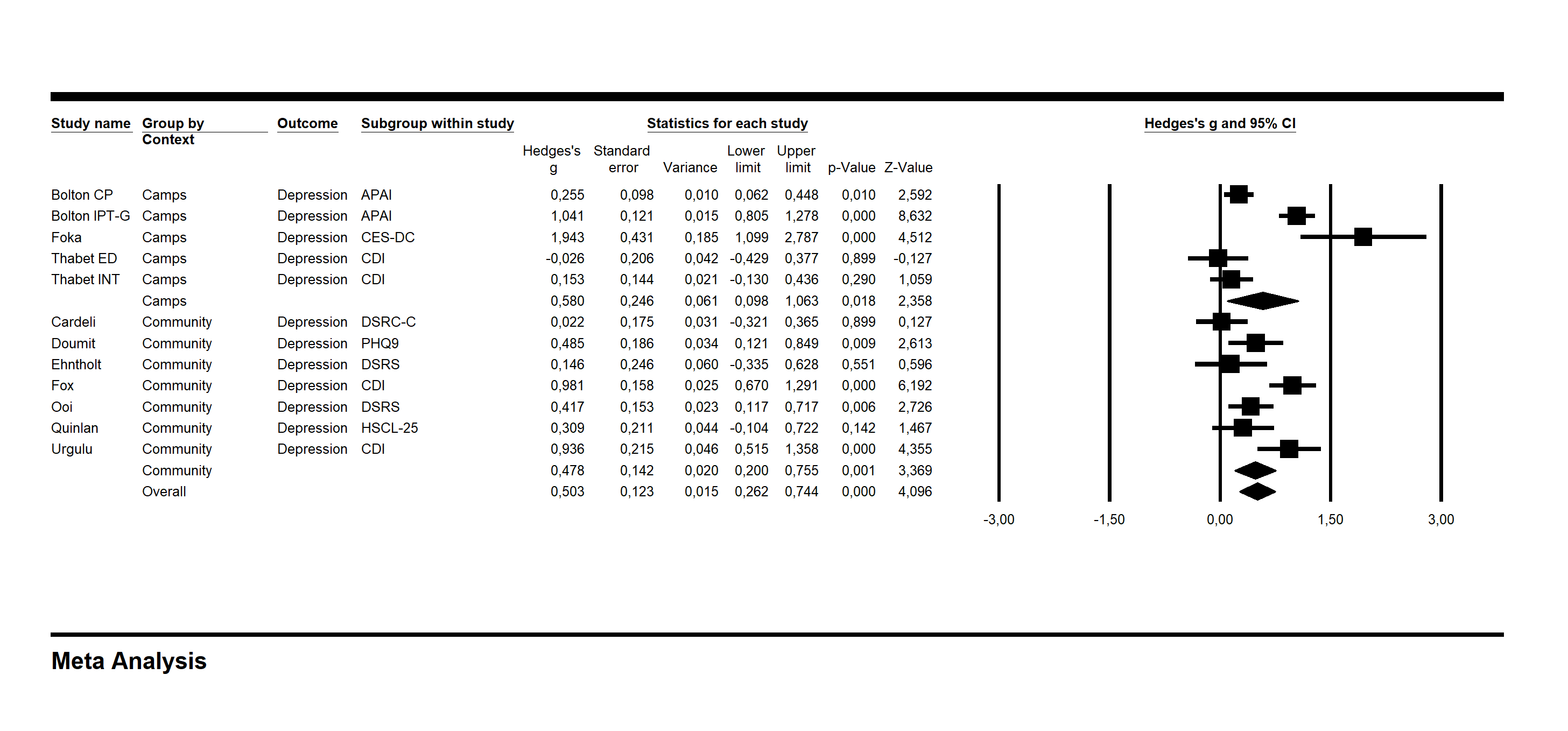


*Note.* CP = Creative Play, IPT-G = Interpersonal Therapy – Group, PS + HCT = Public Schooling plus Tutoring in a Healing Classroom, PS + HCT + M = Public Schooling plus Tutoring in a Healing Classroom plus Mindfulness, APAI = Acholi Psychosocial Assessment Instrument, DSRC-C = Depression Self rating scale for children, PHQ9 = Patient Health Questionnaire 9, DSRS = Birleson Depression Self-Rating Scale – Child version, CES-DC = Center for Epidemiological Studies Depression Scale for Children, CDI = Children’s Depression Inventory, HSCL-25 = Hopkins Symptom Checklist – 25 depression subscale.

**Displacement context.** The forest plot for sub-group analysis of displacement context is presented in Figure 4. The overall effect for participants being internally vs. externally displaced (including refugees and asylum seekers) on depression was significant (*k* = 12, *g* = 0.52, 95% CI [0.26, 0.78]*, z* = 3.90, *p* = < .001, *Q*(1) = 0.58), indicating a medium effect size. A small non-significant effect was observed for internally displaced participants (*k* = 4, *g* = 0.37, 95% CI [-0.10, 0.83], z = 1.55, *p* = .121) and a medium effect for externally displaced participants (*k* = 8, *g* = 0.59, 95% CI [0.27, 0.90], z = 3.63, *p* = <.001). The analysis indicated that improvements were only seen for externally displaced youth.

**Figure 4**

*Forest Plot of Sub-Group Analysis of Within-Group Change in Depression by Internally/Externally Displaced*
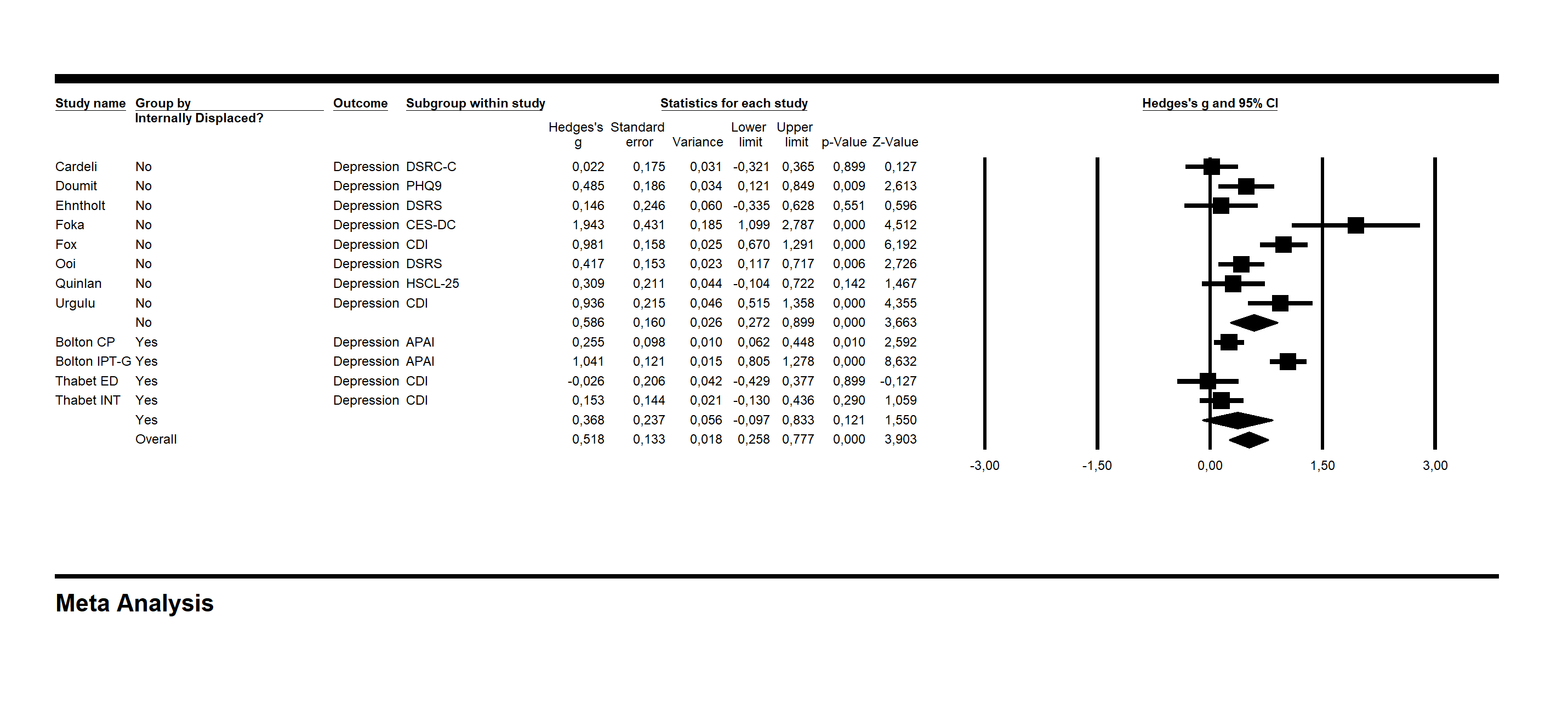


*Note.* CP = Creative Play, IPT-G = Interpersonal Therapy – Group, PS + HCT = Public Schooling plus Tutoring in a Healing Classroom, PS + HCT + M = Public Schooling plus Tutoring in a Healing Classroom plus Mindfulness, APAI = Acholi Psychosocial Assessment Instrument, DSRC-C = Depression Self rating scale for children, PHQ9 = Patient Health Questionnaire 9, DSRS = Birleson Depression Self-Rating Scale – Child version, CES-DC = Center for Epidemiological Studies Depression Scale for Children, CDI = Children’s Depression Inventory, HSCL-25 = Hopkins Symptom Checklist – 25 depression subscale.

**Level of intervention.** The forest plot for sub-group analysis of level of intervention is presented in Figure 4. The overall effect of intervention level (selected vs. indicated) on depression was significant (*k* = 12, g = 0.49, 95% CI [0.22, 0.75]*, z* = 3.65, *p* = < .001, *Q*(1) = 0.93), indicating a small effect size. A medium effect was observed for selected interventions (*k* = 4, *g* = 0.76, 95% CI [0.15, 1.36], z = 2.43, *p* = .015) and a small effect for indicated interventions (*k* = 8, *g* = 0.42, 95% CI [0.14, 0.71], z = 2.90, *p* = .004). The analysis indicated that selected interventions had larger effects on depression than indicated interventions.

**Figure 5**

*Forest Plot of Sub-Group Analysis of Within-Group Change in Depression by Level of Prevention*


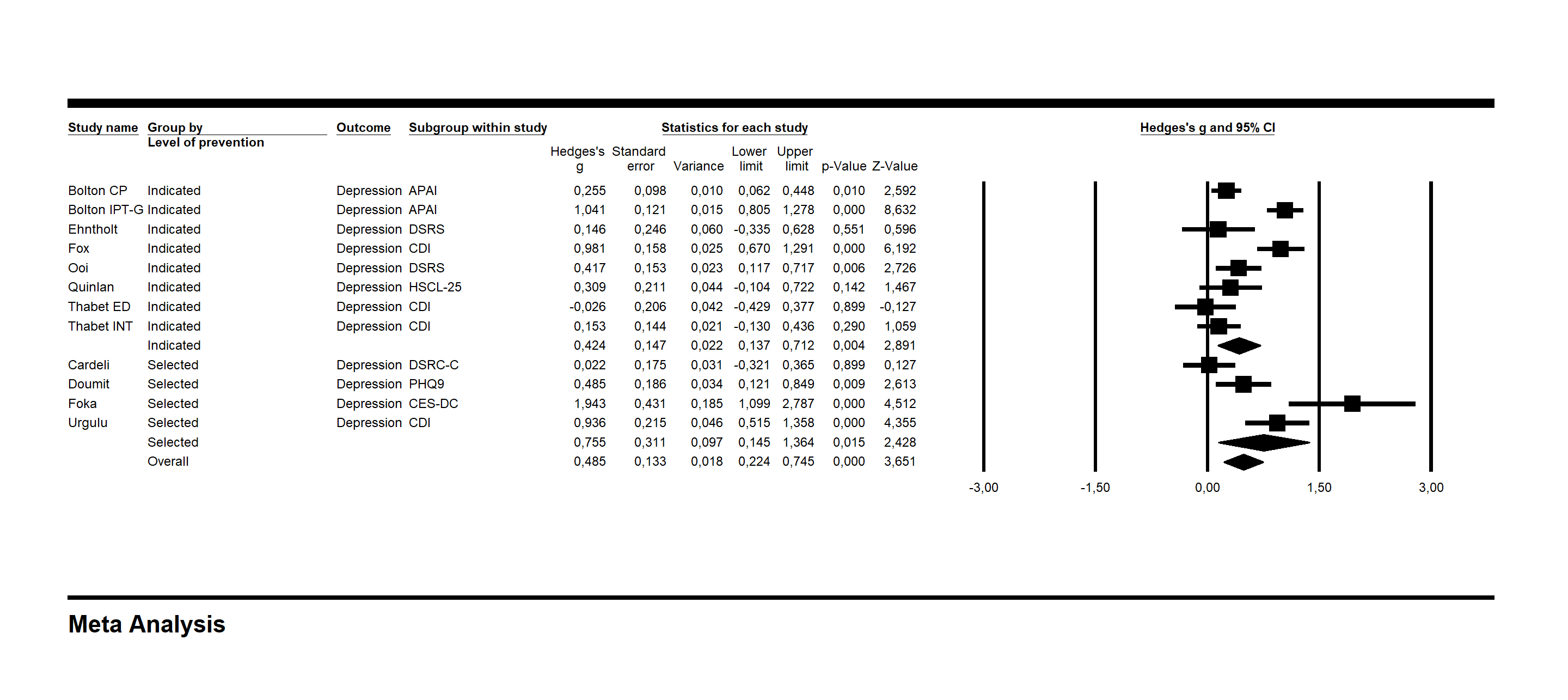


*Note.* CP = Creative Play, IPT-G = Interpersonal Therapy – Group, PS + HCT = Public Schooling plus Tutoring in a Healing Classroom, PS + HCT + M = Public Schooling plus Tutoring in a Healing Classroom plus Mindfulness, APAI = Acholi Psychosocial Assessment Instrument, DSRC-C = Depression Self rating scale for children, PHQ9 = Patient Health Questionnaire 9, DSRS = Birleson Depression Self-Rating Scale – Child version, CES-DC = Center for Epidemiological Studies Depression Scale for Children, CDI = Children’s Depression Inventory, HSCL-25 = Hopkins Symptom Checklist – 25 depression subscale.
